# A selectable, plasmid-based system to generate CRISPR/Cas9 gene edited and knock-in mosquito cell lines

**DOI:** 10.1038/s41598-020-80436-5

**Published:** 2021-01-12

**Authors:** Kathryn Rozen-Gagnon, Soon Yi, Eliana Jacobson, Sasha Novack, Charles M. Rice

**Affiliations:** Laboratory of Virology and Infectious Disease, The Rockefeller University, New York, NY 10065 USA

**Keywords:** Genetic engineering, Non-model organisms, Genetic techniques, Viral vectors

## Abstract

*Aedes (Ae.) aegypti* and *Ae. albopictus* mosquitoes transmit arthropod-borne diseases around the globe, causing ~ 700,000 deaths each year. Genetic mutants are valuable tools to interrogate both fundamental vector biology and mosquito host factors important for viral infection. However, very few genetic mutants have been described in mosquitoes in comparison to model organisms. The relative ease of applying CRISPR/Cas9-based gene editing has transformed genome engineering and has rapidly increased the number of available gene mutants in mosquitoes. Yet, in vivo studies may not be practical for screening large sets of mutants or possible for laboratories that lack insectaries. Thus, it would be useful to adapt CRISPR/Cas9 systems to common mosquito cell lines. In this study, we generated and characterized a mosquito optimized, plasmid-based CRISPR/Cas9 system for use in U4.4 (*Ae. albopictus*) and Aag2 (*Ae. aegypti*) cell lines. We demonstrated highly efficient editing of the *AGO1* locus and isolated U4.4 and Aag2 cell lines with reduced AGO1 expression. Further, we used homology-directed repair to establish knock-in Aag2 cell lines with a 3xFLAG-tag at the N-terminus of endogenous *AGO1.* These experimentally verified plasmids are versatile, cost-effective, and efficiently edit immune competent mosquito cell lines that are widely used in arbovirus studies.

## Introduction

Mosquitoes from the genus *Aedes* are worldwide pests and major vectors of arthropod-borne viruses (arboviruses) that cause global human disease^1–3^. Notable members of this genus include *Ae. aegypti*, which transmits a wide variety of arboviruses, and *Ae. albopictus*, which is prevalent in North America and is an emerging vector for certain arboviruses, such as chikungunya virus^2,4,5^. The ability to perform functional genetic studies in mosquitoes and mosquito cells is crucial to our understanding of pro- and anti-viral mosquito host factors and for potential mosquito control strategies^6–8^. Previous genome engineering in mosquitoes has been achieved using transposons^8–11^ and a variety of engineered nucleases such as transcription activator-like effector nucleases (TALENs)^12,13^, zinc-finger nucleases (ZFNs)^14–17^; and homing endonucleases (HEs)^6,18,19^. However, transposon-mediated transgenesis yields imprecise integrations and it can be laborious to engineer nucleases for each target gene. Difficulties modifying mosquito genomes have been compounded by their large and repetitive nature, which makes assembly and annotation a struggle^20–23^. Therefore, despite their importance to human health, loss-of-function mutants in mosquitoes have significantly lagged behind those available in model insects, such as *Drosophila*.

The adaptation of the bacterial type II clustered regularly interspaced short palindromic repeats (CRISPR) and CRISPR-associated sequence 9 (Cas9) immune system for generalized gene editing has revolutionized genome engineering^24–27^. In CRISPR/Cas9 gene editing, the *Streptococcus pyogenes* Cas9 endonuclease is targeted to genomic DNA by complementary guide RNAs, inducing double-stranded breaks (DSBs; for review of CRISPR/Cas9 see^24^). Genomic loci with DSBs stimulate cellular DNA repair machinery that rejoins DSBs by non-homologous end joining (NHEJ). NHEJ disrupts gene function through small insertions or deletions. Alternatively, cellular homology-directed repair (HDR) can be used to correct the gene or insert changes if a homologous donor template is present. The CRISPR/Cas9 system relies on expression of Cas9, a CRISPR RNA (crRNA) that targets genomic DNA adjacent to a protospacer adjacent motif (PAM; NGG motif) and a trans-activating CRISPR RNA (tracr RNA); crRNA and tracrRNA are often provided together as a single guide RNA (sgRNA). Due to its relative ease of adoption and high efficiency, CRISPR/Cas9-mediated gene editing has generated mutants and knock-ins in a wide variety of cells and organisms^28–31^, including in vivo in mosquitoes^32–36^ (for review see^37^). CRISPR/Cas9-mediated editing is a significant advance in the toolkit for functional genetic studies in mosquitoes. However, not many laboratories have access to insectaries for in vivo experiments, and initial validation of gene function in cells is more practical and cost effective for examining large gene sets. Thus, it is desirable to establish mosquito adapted CRISPR/Cas9 plasmids to generate mutant or knock-in mosquito cell lines; such plasmids have not been reported to-date.

Perhaps due to this lack of mosquito optimized plasmids, there have been relatively few (two) reports of CRISPR/Cas9-edited mosquito cell lines. One study established a clonal cell line (AF5)^38^, which was then used to establish a Dicer-2 defunct AF5 subclone (A319)^39^. The other generated *Nix* gene loss-of-function and knock-in C6/36 cell lines^33^. However, these reports both relied on *Drosophila* CRISPR/Cas9 plasmids^30^ and contain no information on CRISPR/Cas9 editing efficiency. In the current study, we updated CRISPR/Cas9 plasmids that rely on *Drosophila* promoters^29^ with mosquito promoters for use in mosquito cells. We then applied this system to edit widely utilized, immune-competent *Ae. aegypti* (Aag2)^40–42^ and *Ae. albopictus* (U4.4)^42,43^ cell lines. By comparing mosquito adapted CRISPR/Cas9 plasmids to previously used *Drosophila* plasmids, we demonstrated increased editing efficiency of *AGO1*. In mosquitoes and other insects, AGO1 is the main effector of the microRNA (miRNA) pathway^44^. In the miRNA pathway, genome encoded small RNAs (miRNAs) direct AGO1 to target and repress imperfectly complementary transcripts^45^. AGO1 is thus a key regulator of global gene expression during diverse biological processes such as development, blood-feeding and virus infection^46^. AGO1-deficient mosquito cell lines would be useful for interrogating the collective roles of miRNAs in different contexts. We therefore generated *AGO1-*edited U4.4 and Aag2 cell lines with reduced AGO1 protein levels, as well as knock-in Aag2 cell lines that contain a 3xFLAG tag at the N-terminus of endogenous *AGO1*. These well-characterized and efficient mosquito optimized CRISPR/Cas9 plasmids will facilitate functional genetic studies in mosquito cell culture systems.

## Results

### Generation and characterization of mosquito optimized CRISPR/Cas9 plasmids

**Figure 1:**
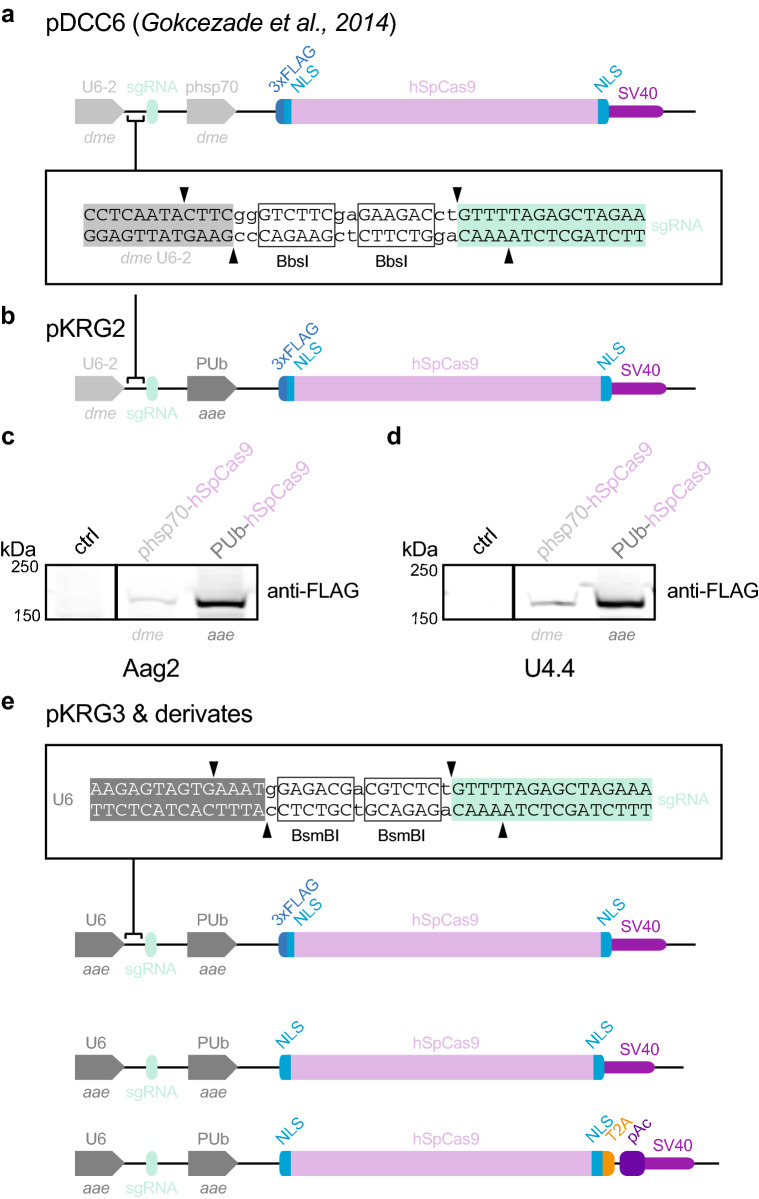
Mosquito optimized CRISPR/Cas9 plasmids. (**a**) pDCC6 plasmid published in Gokcezade et al.^29^. The *dme* U6-2 promoter drives sgRNA transcription and the *dme* phsp70 promoter drives expression of a 3xFLAG-tagged hSpCas9. Guide RNAs are cloned by *BbsI* digest. (**b**) pKRG2 plasmid, generated by replacing the *dme* phsp70 promoter with the *aae* PUb promoter. Cloning of guide RNAs as in (**a**). (**c**) Immunoblot of Aag2 cells treated with transfection reagent (ctrl) or transfected with pDCC6 (3xFLAG-hSpCas9; expressed from the *dme* pshp70 promoter) or with pKRG2 (3xFLAG-hSpCas9; expressed from the PUb promoter). kDa = kilodaltons. Full-length blots in Supplementary Fig. 4. (**d**) As in (**c**) for U4.4 cells. (**e**) To generate pKRG3 mosquito adapted plasmids, the pKRG2 guide RNA cloning site was redesigned to use *BsmBI* (allowing insertion of the *aae* U6 promoter, which is incompatible with the *BbsI* sgRNA cloning site). The RNA Pol II *aae* U6 promoter was then inserted to express sgRNAs. This plasmid was further modified by removing the N-terminal 3xFLAG from hSpCas9 and adding a T2A-pAc to allow puromycin selection to yield several options for mosquito optimized CRISPR/Cas9 editing.

To generate mosquito optimized plasmids for efficient CRISPR/Cas9 gene editing in mosquito cells, we obtained a plasmid used in *Drosophila*, pDCC6^29^. This plasmid relies on two *Drosophila* promoters to express CRISPR/Cas9 components: (1) the RNA Pol III *U6:96Ab*^29,47–51^ (*dme* U6-2) drives transcription of the sgRNA, and (2) the *hsp70Bb*^47,52^ promoter (*dme* phsp70) drives expression of the human codon-optimized *Streptococcus pyogenes* Cas9 (referred to as hSpCas9^26,27,29,53^; Fig. 1a). Because robust hSpCas9 and sgRNA expression is essential for high efficiency editing, we replaced the *Drosophila* promoters in pDCC6 with appropriate *Ae. aegypti* promoters.

We first replaced the *dme* phsp70 with the strong constitutive *Ae. aegypti* polyubiquitin promoter (*aae PUb*^15,32,54^; Fig. 1b). Expression of hSpCas9 in this intermediate plasmid (pKRG2) was then assessed in comparison to the parental pDCC6 plasmid (Fig. 1c,d). We observed increased expression of hSpCas9 in both Aag2 and U4.4 cells using the mosquito PUb promoter. The *dme* phsp70 promoter has long been applied to mosquito cells and, notably, the first stable transformations of mosquito cell lines was performed using this promoter^55^. However, the reported frequency of transformants was quite low, an observation possibly explained by the lower levels of expression we observed from *dme* phsp70 in mosquito cells.

We next updated the U6 promoter to drive sgRNA expression (Fig. 1e). The *Drosophila* U6-2 promoter in the pDCC6 plasmid^29^, which was also used to generate prior CRISPR/Cas9-edited mosquito cells^33,39^, is quite ineffective in mosquito cell lines^56^. We selected the previously described *Ae. aegypti*-derived U6 promoter, *AAEL017774*^57^, which is effective in mosquito cells^56^. These two promoter replacements generate the backbone of the mosquito optimized pKRG3 plasmid (pKRG3-mU6-PUb-3xFLAG-hSpCas9). Because the N-terminal 3xFLAG tag on the hSpCas9 may be undesirable for some applications, we also generated a pKRG3 plasmid with this tag removed (pKRG3-mU6-PUb-hSpCas9).

Finally, we added a selectable marker to pKRG3. Although we optimized transfections (representative transfections of U4.4 and Aag2 shown in Supplementary Fig. 1), no estimates of CRISPR/Cas9-mediated editing efficiency have been reported in mosquito cells. Thus, it may be essential to select transfected cells to increase the likelihood of editing. We generated a plasmid expressing hSpCas9 fused to a selectable marker, a puromycin resistance cassette (pAc). The pAc was inserted after the well-characterized insect virus *Thosea asigna* T2A^58^ ‘2A-like’ ribosome skipping site. This T2A has been applied in *Drosophila* S2 and mosquito (C6/36 and Aag2) cells^30,33,39,59,60^, and was the most efficient skipping site in a comparison of five 2A variations in *Drosophila*^61^. This design ensures co-expression of the hSpCas9 and the pAc selectable marker under one strong, constitutive mosquito promoter (pKRG3-mU6-PUb-hSpCas9-pAc).

**Figure 2:**
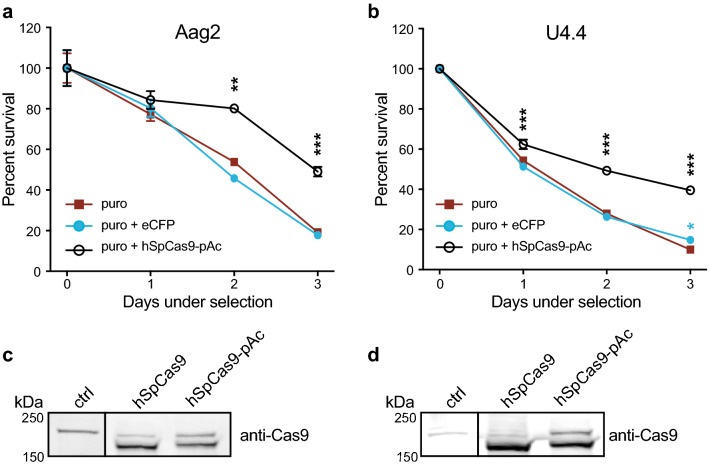
Efficient puromycin selection of mosquito cells transfected with hSpCas9-pAc. (**a**) Aag2 cells were pre-treated with transfection reagent alone (puro), transfected with a control PUb-eCFP (enhanced cyan fluorescent protein) plasmid lacking a puromycin (puro) resistance cassette (puro + eCFP), or transfected with PUb-hSpCas9 linked to a puro resistance cassette by the T2A skipping site (hSpCas9-pAc). Day 2 post-transfection, cells were treated with 2.5 µg/mL puro. *p* < 0.0001 (overall ANOVA comparing different transfections); individual groups were compared using the Dunnett's post hoc test compared to the puro control; **p < 0.001, ***p ≤ 0.0001. (**b**) As in (**a**) for U4.4 cells treated with 10 µg/mL puro. *p < 0.05, ***p ≤ 0.0001. (**c**) Immunoblot of Aag2 cells treated with transfection reagent alone (ctrl) or transfected with hSpCas9 or hSpCas9-pAc (driven by PUb promoter). Expected size of hSpCas9 is ~ 160 kilodaltons (kDa); expected size of unprocessed hSpCas9-pAc is ~ 185 kDa. Full-length blots in Supplementary Fig. 4. (**d**) As in (**c**) for U4.4 cells.

To assess the suitability of puromycin selection in Aag2 and U4.4 cells, we performed puromycin kill curves to titrate the optimal concentration (data not shown). Puromycin treatment killed both cell lines efficiently in the absence of pAc expression, and PUb-driven expression of hSpCas9-pAc significantly increased cell viability in the presence of puromycin (Fig. 2a,b). We additionally confirmed efficient T2A skipping in both mosquito cell lines by examining the size of hSpCas9 by immunoblot (Fig. 2c,d). In both cases, the hSpCas9 was processed correctly and we observed a strong band at the expected size (~ 160 kDa) in both constructs. The slight shift in hSpCas9-pAc is due to additional amino acids in the T2A upstream of the skipped residue, and is consistent with T2A processing in *Drosophila*^59^. A higher nonspecific band is present in all lanes and does not reflect unprocessed hSpCas9-pAc, which would run at ~ 185 kDa. Therefore, PUb-hSpCas9-pAc constructs correctly express and process hSpCas9 and enable rapid selection in U4.4 and Aag2 cells.

### Efficient CRISPR/Cas9 editing of AGO1 in U4.4 cells using mosquito-adapted plasmids

**Figure 3:**
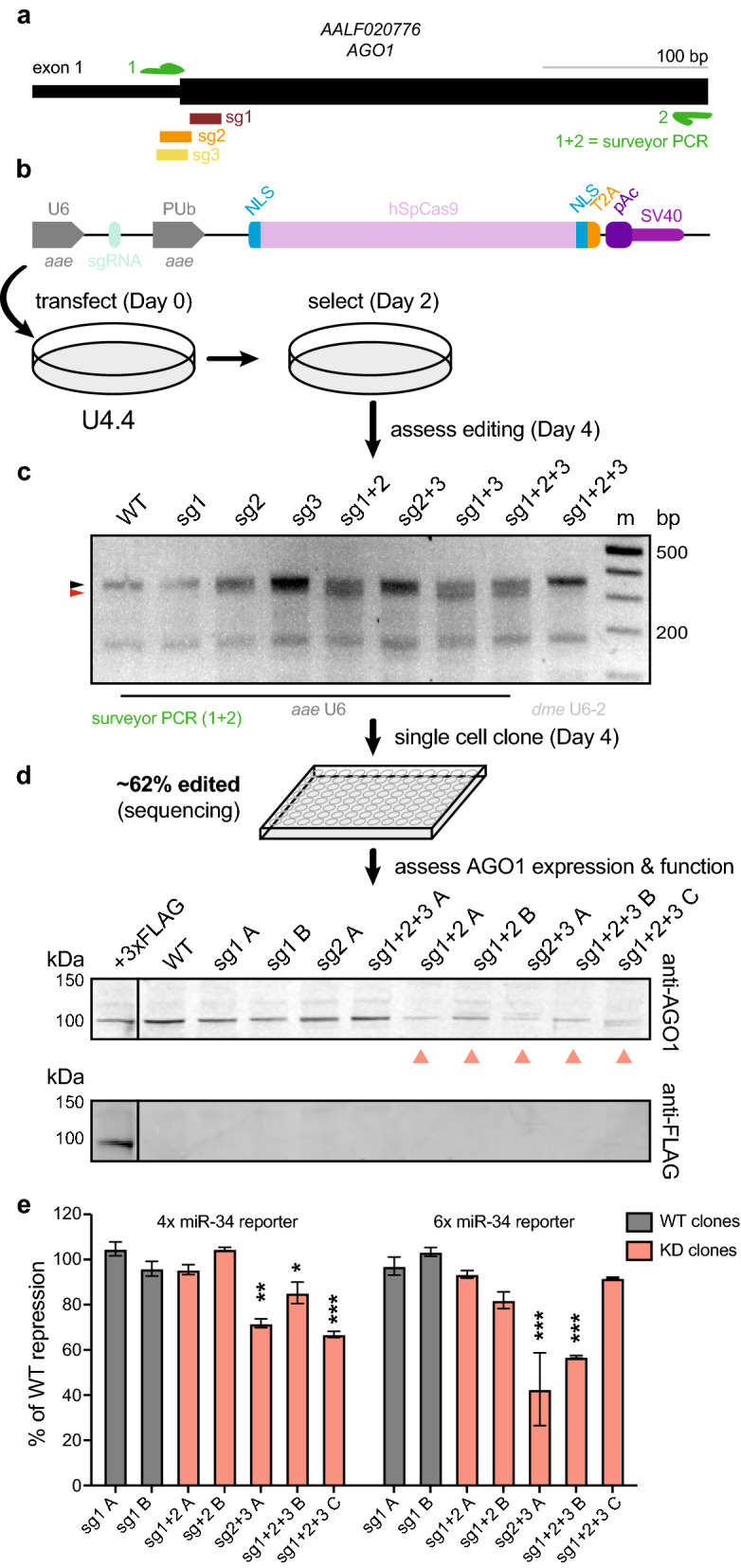
Isolation of AGO1-deficient U4.4 clonal cell lines. (**a**) Three sgRNAs were designed near the translation start site for the *AGO1* locus in U4.4 cells. Note the diagram shown differs from the *Ae. albopictus* annotation (AaloF1.2) and is based on sequencing of U4.4 cells. A PCR was designed for surveyor assay (primers = green arrows). (**b**) U4.4 *AGO1* sgRNAs were cloned into pKRG3, and U4.4 cells were transfected with these plasmids singly or in combination. Workflow to obtain edited cells is shown. (**c**) Editing efficiency was assessed by surveyor assay. Expected size of wild-type (WT) amplicon =  ~ 350 base pairs (bp; black arrow); expected size of digested fragments based on sgRNA cleavage sites =  ~ 330 bp (red arrow) +  ~ 23 bp (not visible), In comparison to the *aae* U6 promoter, no editing is observed with the same guides using the *dme* U6-2 promoter (both plasmids express hSpCas9 using PUb promoter). m = marker. (**d**) Single cells clones were sequenced to assess the percentage of edited clones. Immunoblot of AGO1 (top) showed clones with WT and reduced (salmon arrows) AGO1 protein levels; A, B, C denote clones. The ectopically expressed, 3xFLAG-tagged U4.4 AGO1 was detected by both the anti-AGO1 antibody and the anti-FLAG antibody (bottom). (**e**) Luciferase reporter assay measuring repression of 4 or 6 repeated miR-34 sites (4 ×, 6 × miR-34 reporter). Repression for each clone was measured by normalizing to internal transfection controls (reporter luciferase/control luciferase), then to unrepressed reporters in the same clone. The percent (%) of repression compared to WT clones sg1 A and sg1 B is shown. *p* < 0.0001 (overall ANOVA comparing different clones); individual groups were compared using the Dunnett's post hoc test compared to the WT clone sg1 A; *p < 0.05, **p < 0.001, ***p ≤ 0.0001. All full-length immunoblots in Supplementary Fig. 4; all full-length DNA gels in Supplementary Fig. 5.

As proof-of-principle, we investigated whether the mosquito optimized pKRG3 CRISPR/Cas9 plasmid would enable editing of U4.4 cells at the *AGO1* locus. Guides were designed against the experimentally determined U4.4 genomic sequence near the *AGO1* translational start site by searching for PAM NGG sequences. Because no U4.4 genome has been published, we first verified the starting methionine to target by 5′RACE (this differed from the annotated start site; unpublished results). Three guides were designed in the first exon near the beginning of the coding sequence in order to disrupt *AGO1* (Fig. 3a). Guides were cloned into pKRG3 and these plasmids were transfected singly or in combination into U4.4 cells (Fig. 3b). Post-puromycin selection, we assessed editing efficiency by surveyor assay (Fig. 3c). In this assay, mismatches between annealed wild-type (WT) and edited amplicons lead to cleavage by the surveyor nuclease. Un-transfected WT cells exhibited the expected amplicon at ~ 350 bp (black arrow; and a smaller, nonspecific band). In contrast, U4.4 cells transfected with pKRG3 plasmids revealed a slightly lower band (red arrow) in addition to the WT band. This indicates amplification of both WT and CRISPR/Cas9-edited *AGO1* loci from pKRG3-transfected cells. We observed that editing was not equally efficient for all single guides, although they were all designed in close proximity. Further, combinatorial transfections with two or three guides performed by far the best^47,50,51^. For a head-to-head comparison, we additionally examined editing using the same hSpCas9 expression and puromycin selection conditions with the *dme* U6-2 promoter (pKRG2) instead of the *aae* U6 promoter (pKRG3). When guides were expressed from the *dme* U6-2 promoter we did not observe any editing, even using all three guides in combination. Therefore, in U4.4 cells the pKRG3 plasmid substantially increases editing efficiency compared to plasmids that rely on *dme* U6-2 for sgRNA expression.

We next isolated single U4.4 cell clones from this edited population to establish clonal cell lines and assess their potential edits and AGO1 protein levels (Fig. 3d). Sequencing of clonal lines showed disruptive edits corresponding to sgRNA cleavage sites (~ 65% of clones were edited; example alignment shown in Supplementary Fig. 2a). Immunoblot showed decreased protein levels in several of the isolated clones compared to WT levels (Fig. 3d, top). Consistently, we observed that most clones with lower AGO1 levels were isolated from cell populations transfected with combinations of guides. Detection of an ectopically expressed 3xFLAG-tagged U4.4 AGO1 confirmed detection of mosquito AGO1 using the immunoblot antibody (Fig. 3d, bottom). Interestingly, we were unable to isolate a knock-out clone completely lacking AGO1 protein, despite isolating a variety of different edited lines with reduced AGO1 levels.

To confirm that reduced AGO1 protein levels corresponded with reduced AGO1 function, we performed a microRNA (miRNA) reporter assay. In mosquitoes, small, genome encoded miRNAs direct AGO1 to repress imperfectly complementary transcripts^45^. We assessed whether U4.4 clones with WT or reduced AGO1 expression could use endogenous miR-34 to repress a *Renilla* luciferase reporter gene containing miR-34 target sequences (Fig. 3e). While neither WT clone was functionally impaired, several of the clones deficient in AGO1 exhibited consistently reduced repression. However, we note that AGO1 protein level was not strictly correlated with functional repression. These data indicate that mosquito optimized CRISPR/Cas9 plasmids can be used to edit *AGO1* and to impair AGO1 expression and function in U4.4 cells.

### CRISPR/Cas9 mediates efficient editing and allows knock-in of AGO1 in Aag2 cells

**Figure 4:**
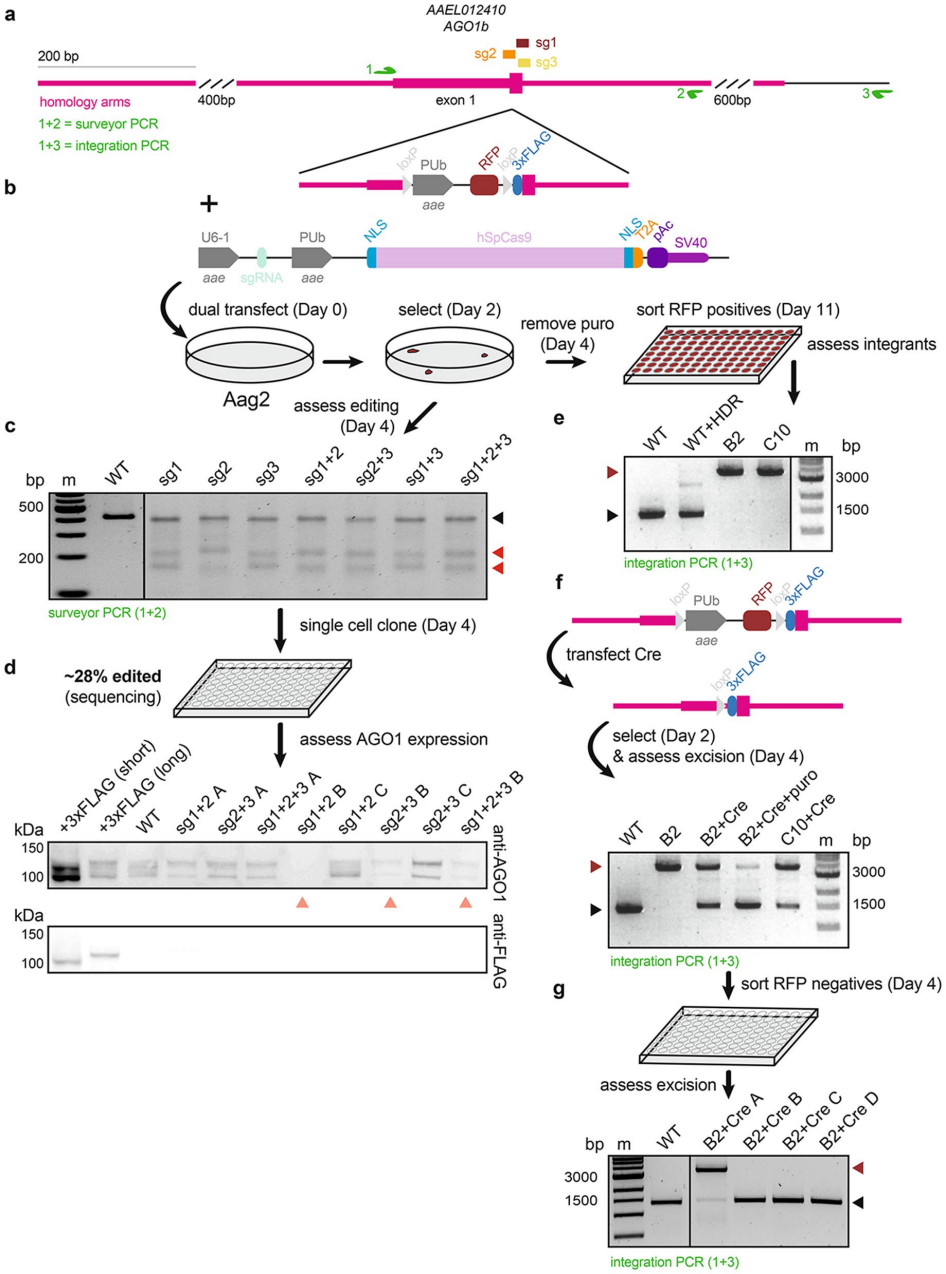
CRISPR/Cas9-mediated editing and knock-in of *AGO1* in Aag2 cells. (**a**) Three sgRNAs (sg) were designed near the *AGO1* translation start site in Aag2 cells. PCRs were designed for surveyor assay (green arrows 1 and 2) and to assess integration (green arrows 1 + and 3). (**b**) Homology-directed repair (HDR) donor template design. The PUb promoter drives expression of red fluorescent protein (RFP); the PUb-RFP cassette is flanked by two loxP sites, followed by a 3xFLAG-tag. Aag2 *AGO1* sgRNAs transfections were performed as in Fig. 3b with the HDR donor template added. Workflows to obtain edited or integrated clones are shown. (**c**) Editing efficiency was assessed by surveyor assay. Expected size of wild-type (WT) amplicon =  ~ 410 base pairs (bp; black arrow); expected size of digested fragments based on sgRNA cleavage sites =  ~ 180 bp +  ~ 230 bp (red arrow); m = marker. (**d**) Single cell clones were sequenced to determine the percentage of edited clones. Immunoblot of AGO1 (top) showed clones with WT and reduced AGO1 protein levels (salmon arrows); A, B, C denote clones. 3xFLAG-tagged Aag2 AGO1 short and long isoforms were detected by both the anti-AGO1 antibody and the anti-FLAG antibody (bottom). (**e**) RFP-positive Aag2 clones from (**b**) were screened for HDR-mediated integration of the donor template. Expected size of amplicons from WT clones =  ~ 1500 bp (black arrow); expected size of amplicons from clones containing the integrated HDR donor template =  ~ 3700 bp (dark red arrow). B2 and C10 clones were knock-in at the *AGO1* locus*.* (**f**) The PUb-RFP cassette was excised from Aag2 *AGO1* knock-in clones (B2 and C10) by transfection of PUb-driven Cre-T2A-pAc and puro selection. WT and un-transfected B2 cells are shown; expected WT and integrated HDR donor template amplicon sizes as in (**e**); expected size of Cre excised amplicon =  ~ 1500 bp (black arrow). Puro treatment increases the proportion of Cre excision (B2 + Cre + puro). (**g**) B2 and C10 Cre excised cell lines were sorted for RFP negative clones to obtain homozygous knock-in 3xFLAG-*AGO1* Aag2 cell lines. Expected amplicon sizes as in (**e**–**f**). All full-length immunoblots in Supplementary Fig. 4; all full-length DNA gels in Supplementary Fig. 5.

We next asked whether mosquito adapted plasmids would also allow editing of *Ae. aegypti* Aag2 cells. In addition to isolating AGO1-deficient cell lines, as in U4.4, we also aimed to generate a knock-in *AGO1* Aag2 cell line by applying CRISPR/Cas9 editing and providing a homology-directed repair (HDR) donor template. Not only are Aag2 cells are widely used in arbovirus studies, thus far, knock-in *Ae. aegypti* cell lines have not been reported. Further, gene annotation is more reliable in *Ae. aegypti* than in *Ae. albopictus*, facilitating the design of HDR donor templates. We thus used the *AGO1* locus to provide proof-of-principle that the CRISPR/Cas9 system can be used to generate *Ae. aegypti* knock-in cell lines. We designed three guide RNAs near the *AGO1* starting methionine, as in U4.4 cells (Fig. 4a). Additionally, we designed an HDR donor template to insert an N-terminal 3xFLAG tag at the endogenous *AGO1* locus (Fig. 4b). Because HDR can be very inefficient, upstream of the 3xFLAG we inserted a red fluorescent protein (RFP) driven by the PUb promoter, flanked by two loxP sites. This allows sorting of RFP-positive cells to isolate potentially rare clones with the PUb-RFP-3xFLAG integrated. The PUb-RFP marker can then be excised via expression of Cre recombinase, leaving only a small scar upstream of the 3xFLAG-tagged *AGO1*. This entire cassette was flanked by ~ 1 kb of homology on either terminus^30^.

Aag2 cells were transfected with pKRG3 plasmids singly or in combination, with the linearized HDR donor template (Fig. 4b). As in U4.4 cells, editing efficiency of bulk Aag2 cells was assessed post-puromycin selection. In contrast to in U4.4 cells, in Aag2 cells we consistently observed high editing efficiency whether guides were expressed singly or in combination (Fig. 4c). Isolation of single cell clones followed by sequencing showed ~ 28% of isolated clones contained edits (example alignment shown in Supplementary Fig. 2b). Immunoblot showed several clones with decreased and one clone with ablated AGO1 expression (clone sg1 + 2 B; Fig. 4d, top). We were able to detect 3xFLAG-tagged short and long Aag2 AGO1 isoforms by ectopic expression or constitutive expression in stably transformed cell lines, confirming reliable detection of mosquito AGO1 (Fig. 4d, bottom). Unfortunately, the single AGO1 knock-out clone grew poorly and could not be expanded to sufficient cell numbers to establish a knock-out cell line. We were able to establish several Aag2 clonal cell lines with lower AGO1 expression, as with U4.4. However, expansion and subsequent culturing of AGO1-deficient Aag2 cell lines was much more difficult than in U4.4. Unlike in U4.4 cells, in Aag2 cells, despite isolating clones with decreased AGO1 protein, none of the three clones were significantly and consistently impaired in AGO1 function (Supplementary Fig. 3). Two of the three clones examined had slightly reduced AGO1 function, but the effect was subtle and generally not significant. It remains unclear why, in particular for Aag2 cells, even clones with large reductions in AGO1 protein levels (e.g. sg1 + 2 D) were only subtly impaired in AGO-mediated target repression.

Although we observed high editing efficiencies and isolated clones with decreased AGO1 expression, no AGO1 knock-in cell lines were isolated by serial limiting dilution (Fig. 4d). Therefore, after transfection and selection, cells were cultured until the input HDR donor template RFP signal diminished. Cells were then sorted to identify clones that were RFP-positive due to knock-in of the donor template (Fig. 4b). Consistent with the lack of knock-in clones obtained by serial limiting dilution, the efficiency of HDR was very low and only ~ 0.1% of cells were RFP-positives. We screened RFP-positive single cell clones by PCR and identified two homozygous clones (B2 and C10) with the correct integration, indicated by a ~ 3700 bp product (compared to the WT amplicon of ~ 1500 bp; Fig. 4e). Our primer design outside of the homology arms ensured that this screening PCR only detects the integrated HDR donor template. (WT + HDR control lane). To excise the loxP-PUb-RFP-loxP cassette, we transfected cells with a plasmid expressing a puromycin-selectable Cre recombinase using the PUb promoter (pKRG4-mPUb-Cre-pAc; Fig. 4f). Upon Cre expression, ~ 50% of the selectable cassette was excised; this percentage could be increased by selecting for Cre-transfected cells with puromycin. To generate the final 3xFLAG-tagged *AGO1* knock-in cell lines, RFP-negatives, which were abundant, were again sorted following Cre transfection and selection (Fig. 4g). PCR of established B2 and C10 subclones indicated homozygous excision. Therefore, CRISPR/Cas9 editing and HDR were applied to commonly used Aag2 cells to knock-in a 3xFLAG tag at the N-terminus of the endogenous *AGO1* locus.

**Figure 5:**
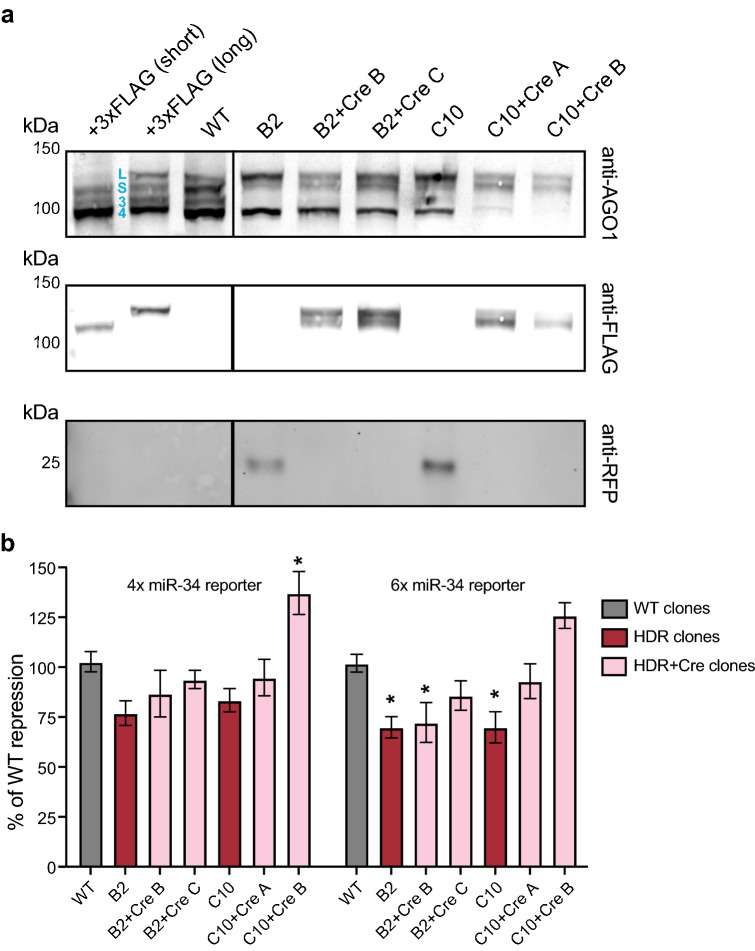
Characterization of *AGO1* knock-in Aag2 cell lines. (**a**) Immunoblot of AGO1 (top) for wild-type (WT) and knock-in Aag2 clones. 3xFLAG-tagged Aag2 AGO1 short (blue S) and long (blue L) isoforms were detected by both the anti-AGO1 antibody and the anti-FLAG antibody (middle). Two shorter isoforms are indicated (blue 3, 4). 3xFLAG-tagged long and short AGO1 isoforms are detected in clones established following Cre excision (+ Cre A, B, C) and are expressed from the endogenous *AGO1* locus. RFP (bottom) is detected in clones with the integrated HDR donor template (B2, C10) but not in clones established following Cre excision. Full-length immunoblots in Supplementary Fig. 4. (**b**) Luciferase reporter assay measuring repression of 4 or 6 repeated miR-34 sites (4 ×, 6 × miR-34 reporter). Normalization was performed as in Fig. 3e. The percent (%) of repression compared to WT is shown. *p* < 0.0001 (overall ANOVA comparing different clones); individual groups were compared using the Dunnett's post hoc test compared to WT cells; *p < 0.05.

We next verified 3xFLAG-tagged AGO1 expression for knocked-in Cre excised Aag2 cell lines (Fig. 5a). We generally observed four AGO1 isoforms expressed in Aag2 cells, although the expression of each isoform often varied between clones (Figs. 4d, 5a). The two largest isoforms were experimentally verified using 5′RACE (unpublished results) and are expressed with 3xFLAG tags as immunoblot controls; both the short (S) and long (L) isoform arise from the same transcriptional start site. The smaller two isoforms (termed 3, 4 in Fig. 5) have not been verified by sequencing but are often decreased with the other two isoforms (Fig. 4d), and therefore likely also represent *bona fide* AGO1 isoforms. Expression levels of the short and long AGO1 isoforms were fairly consistent between clonal lines and WT cells, while expression of the smaller isoforms (3, 4) were more variable (Fig. 5a, top). Interestingly, we found that although the short and long AGO1 isoforms were still expressed in B2 and C10 clones containing the integrated PUb-RFP cassette, they were not 3xFLAG-tagged (Fig. 5a, middle). The lack of 3xFLAG on the AGO1 isoforms expressed in B2 and C10 clones may indicate that the PUb-RFP cassette alters the *AGO1* transcriptional start site. Upon excision, as expected, both AGO1 short and long isoforms were confirmed to contain 3xFLAG tags (+ Cre lines). RFP was only detected in B2 and C10 clones and was successfully excised in the Cre-transfected subclones (Fig. 5a, bottom). These results confirmed that Cre excised, knock-in Aag2 cells express 3xFLAG-tagged AGO1 short and long isoforms from the endogenous *AGO1* locus.

After determining that 3xFLAG-tagged AGO1 isoforms were detected in Cre excised knock-in cell lines, we evaluated the functionality of 3xFLAG-tagged AGO1 in the miRNA reporter assay (Fig. 5b). We only observed defects in AGO1-mediated repression using the 6 × miR-34 reporter, where we saw a moderate decrease in silencing efficiency in particular for the B2 and C10 clones containing the integrated HDR donor template (HDR clones). This defect was largely alleviated upon Cre excision, with the exception of one excised clone (B2 + Cre B). Thus, most of the 3xFLAG-tagged knock-in lines maintain WT functional repression of miR-34 targets. These Aag2 knock-in lines will facilitate a wide variety of studies of endogenous, functional mosquito AGO1.

## Discussion

This study provides the first detailed overview and optimization of CRISPR/Cas9 editing in mosquito cells. We generated a set of versatile, selectable CRISPR/Cas9 plasmids, updated for use in mosquito cells. First, by replacing the *Drosophila* hsp70 promoter with the *Ae. aegypti* PUb promoter, we demonstrated increased hSpCas9 expression in both mosquito cell lines examined. We also verified correct processing of the T2A ribosomal skipping site, enabling constitutive hSpCas9 and pAc expression from the same promoter (Fig. 2). This strategy was previously employed to generate CRISPR edited Aag2 and C6/36 cells using the same hSpCas9-T2A-pAc under the *Drosophila* Actin-5c promoter^30,33,39^. We showed that expression of hSpCas9 or Cre fused to T2A-pAc conferred resistance to puromycin and that puromycin treatment increased the desired cell population (Figs. 2, 4). Second, we replaced the *dme* U6-2 promoter with an *aae* promoter for sgRNA expression. Although we saw high levels of editing of the *AGO1* locus using the *aae* U6 promoter, we could not detect any editing using the *dme* U6-2 promoter used in the prior mosquito cell studies^33,39^. This observation is consistent with previous results that the *dme* U6-2 promoter is not very active in mosquito cells^56^. Thus, the mosquito optimized CRISPR plasmids reported here are a significant improvement upon the *Drosophila-*based plasmids previously used in mosquito cells^33,39^.

Our results with *AGO1* demonstrate that a plasmid-based CRISPR/Cas9 system works well in mosquito cells. Using the updated mosquito CRISPR/Cas9 plasmids, we obtained ~ 28 to 65% edited *AGO1* clones (Figs. 3, 4). Co-delivery and expression of both hSpCas9 and sgRNAs from the same, selectable plasmid may increase editing efficiency. Ultimately, high editing efficiencies reduce the labor involved expanding and screening clones, enabling functional interrogation of larger gene sets. Further, selecting bulk edited cells rather than starting with a clonal cell line for editing^39^ allows isolation of multiple WT and edited clonal cell lines. Surveying multiple WT and edited clones gives an accurate impression of the phenotypic variation obtained for a given gene mutant from cell to cell. For example, we observed reduced ability to repress miR-34 reporters for the majority of U4.4 AGO1-deficient clones but observed variability in the degree of AGO1 impairment.

Interestingly, despite high editing efficiency of the *AGO1* locus in both cell types examined, we were only able to isolate clones with reduced AGO1 protein, not complete knock-outs. The observed reduction in AGO1 protein levels could be due to heterozygous clones or to gene mutations that disrupt, but do not fully ablate, AGO1 expression. Where clonal cell lines were isolated by serial limiting dilution, it also remains possible that some of the AGO1-deficient lines obtained are, in fact, mixed, not clonal populations. Whatever the mechanistic explanation for reduced AGO1 protein levels as opposed to complete ablation of AGO1 expression, this result was extremely consistent in both mosquito cell types examined. We hypothesize that there is selective pressure against complete AGO1 knock-outs, which would be consistent with the impaired growth observed for miRNA-deficient *Drosophila* and mammalian cells^30,62^. Indeed, the only Aag2 cell clone that we identified completely lacking AGO1 could not be expanded. Further, even Aag2 clones with reduced AGO1 levels were difficult to culture, and the clones we were able to expand to sufficient numbers to perform miRNA reporter assays did not generally exhibit significant impairment of AGO1 function. Although we were able to isolate several U4.4 clones that were consistently impaired in AGO1 function, we also noted there was not a strict correlation between AGO1 protein levels and functional impairment in U4.4 cells. Thus, AGO1-deficient clones that grow well may contain specific mutations that decrease protein levels but largely do not impact AGO1 function. It is also possible that some clones upregulate compensatory transcripts related to AGO1, as has been observed for other genetic mutants^63,64^. Our results are perhaps unsurprising, given the important regulatory roles played by the miRNA pathway. In the future, CRISPR/Cas9 editing of *AGO1* in the presence of inducible AGO1 expression may circumvent negative effects on cell growth and allow isolation of true knock-outs. However, the present results clearly indicate that CRISPR/Cas9 editing is efficient in both the mosquito cell lines examined and can be used to disrupt AGO1 expression.

Notably, we were also able to use CRISPR/Cas9 plasmids in combination with an HDR donor template to generate Aag2 *AGO1* knock-in cells. In contrast to in *Ae. albopictus* cells, knock-in *Ae. aegypti* cell lines generated using CRISPR/Cas9 genome engineering have not been reported to-date. Therefore, to our knowledge, these Aag2 lines expressing 3xFLAG-tagged AGO1 are the first examples of CRISPR/Cas9-mediated knock-ins in an *Ae. aegypti* cell line. Consistent with previous studies^65^, we show that the N-terminal 3xFLAG tag does not interfere with AGO1’s function repressing miRNA targets. Thus, these cell lines represent valuable tools to better characterize endogenous mosquito AGO1. Although the efficiency of HDR-mediated integration was low, 0.1% was still sufficiently high to isolate *AGO1* knock-in Aag2 cells. Further, the robust CRISPR/Cas9 system we report will permit future optimization of HDR efficiency in mosquito cells. The ability to knock-in large cassettes and excise them allows further flexibility in tagging endogenous proteins for mechanistic studies or generating conditional knock-outs in mosquito cells.

In all, we generated a versatile, effective, single plasmid system for the generation of CRISPR/Cas9 edited mosquito cell lines. The mosquito adapted plasmids we report are a cost-effective tool to screen and investigate functional phenotypes for a large number of gene mutants. This easily customizable set of plasmids can also be updated to encode different Cas9 variants for other applications, such as blocking gene transcription (CRISPR interference) or increasing gene expression (CRISPR activation). It is our hope that this system will facilitate functional genetic studies using widely accessible, immune-competent cell models for major vectors of arboviruses.

## Methods

### Cell lines

Mosquito U4.4 (*Ae. albopictus*) and Aag2^40^ (*Ae. aegypti*) cell lines were kind gifts from Dr. Dennis Brown and from Dr. Maria Carla Saleh, respectively. Cell lines were grown in Leibovitz's L-15 Medium, no phenol red (Thermo Fisher Scientific), supplemented with 20% (U4.4) or 10% (Aag2) fetal bovine serum (FBS, Hyclone, GE Healthcare), 0.1 mM non-essential amino acids (Thermo Fisher Scientific), and ~ 0.3 g/L tryptose phosphate broth (Sigma-Aldrich) at 28 °C, 0% CO_2_.

### Plasmid generation

All CRISPR/Cas9 plasmids were generated from the pDCC6 plasmid, which encodes the human codon-optimized *Streptococcus pyogenes* Cas9 (hSpCas9^29,53^; a gift from Peter Duchek (Addgene plasmid #59985; http://n2t.net/addgene:59985; RRID:Addgene_59985). All oligos and gBlocks Gene Fragments were purchased from IDT (see Supplementary Table 1 for all oligo and gBlock sequences). All restriction enzymes, calf intestinal phosphatase (CIP), T4 DNA ligase and Gibson Assembly Master Mix were purchased from NEB, and digests and ligations were performed according to the manufacturer’s protocol. Polymerase chain reactions (PCRs) were performed using Phusion DNA polymerase (NEB) according to manufacturer’s protocols. All transformations were performed using in-house DH5alpha chemically competent cells according to standard protocols. Plasmids were isolated from bacteria using the QIAprep Spin Miniprep Kit (Qiagen) and DNA purifications were performed using QIAquick Gel Extraction and QIAquick PCR Purification Kits (Qiagen), all according to the manufacturer’s protocols. All plasmids were sequence-verified and pKRG3 and HDR donor template sequences are available in the Supplemental Information and at Addgene (plasmids 162161–162164). Please see the Supplemental Methods in the Supplemental Information file for detailed cloning procedures.

### General transfections

Aag2 and U4.4 cells were transfected with the appropriate plasmids using Fugene HD Transfection Reagent (Promega) according to the manufacturer’s protocol. Cells were seeded at ~ 50% confluency in 12-well plates and complexes were formed using a ratio of 3:1 transfection reagent to plasmid DNA (500 ng total plasmid/well). In the case of transfection with multiple clones, the total DNA concentration was kept constant and individual plasmids were mixed at equal concentrations.

### Cell survival assays

Cells were seeded at 50% confluency into 96-well plates and transfected with no plasmid, an eCFP control plasmid lacking the pAc (pSL1180-HR-PUbECFP), or a PUb-hSpCas9-pAc plasmid (pKRG2). Day 2 post-transfection, media was replaced with puromycin-containing media (2.5 µg/mL for Aag2 cells and 10 µg/mL for U4.4 cells). At Day 0, 1, 2, and 3 post-puromycin addition, cell survival was analyzed using the CellTiter-Glo Luminescent Cell Viability Assay (Promega) and a FLUOstar Omega Microplate Reader (BMG LABTECH), according to the manufacturer’s instructions. Luciferase values at each day post-puromycin addition were normalized by plasmid paired untreated controls. Survival at each day by plasmid was further normalized to the measured survival for that plasmid at Day 0. Five replicates were collected per condition. Data were analyzed using two-way analysis of variance (ANOVA) with Dunnett’s post hoc test, compared to the transfection control without plasmid (Prism 8).

### Generation of stable cell lines

To generate Aag2 cells stably expressing the long AGO1 isoform with an N-terminal 3xFLAG tag, Aag2 cells were transfected with pKRG4-mPUb-3xFLAG-Aag2-AGO1-long-pAc. Day 2 post-transfection, Aag2 cells were treated with a low concentration of puromycin (1 µg/mL) and maintained until cultures recovered (~ 2 weeks). 3xFLAG-tagged AGO1 expression was confirmed by immunoblot in polyclonal transformants.

### CRISPR/Cas9 transfections

Mosquito cells were transfected with pKRG3-mU6-PUb-hSpCas9-pAc plasmids each containing an AGO1 sgRNA singly, or in combination. Day 2 post transfection, puromycin-containing media was added (2.5 µg/mL, Aag2; 10 µg/mL, U4.4) and cells were selected for an additional 2 days. Post-selection, cell pellets were collected and screened for editing efficiency.

For Aag2 knock-in cells, transfections were performed as above but linearized pSL1180-HR-Aag2-loxP-PUb-RFP-loxP-3xFLAG-AGO1 (*XcmI/AflII*) was co-transfected.

### Surveyor assays

Genomic DNA was isolated from bulk cells transfected with CRISPR/Cas9 plasmids using the epicentre QuickExtract DNA Extraction Solution (Lucigen) according to the manufacturer’s protocol. PCRs were amplified from genomic DNA using primers RU-O-22929 and RU-O-24042 for U4.4 cells (full-length PCR product = 353 bp, digested =  ~ 330 bp +  ~ 23 bp); primers RU-O-22776 and RU-O-22777 were used for Aag2 cells (full-length PCR product = 412 bp, digested =  ~ 179 bp +  ~ 231 bp). PCRs were screened for editing efficiency using the Surveyor Mutation Detection Kit (IDT) according to the manufacturer’s instructions, and treated amplicons were visualized on ~ 1% agarose gels with 100 bp DNA ladder (NEB) and SYBR Gold (Thermo Fisher Scientific).

### Single cell cloning

For bulk CRISPR/Cas9-transfected cells with apparent editing, we isolated single cells to establish clonal edited cell lines. This was done by serial dilution: in brief, cells were resuspended to a concentration of ~ 7 cells/mL and aliquoted into 96-well plates (~ 0.7 cells/well) in 50% fresh media, and 50% conditioned media. Cells were expanded and screened by PCR (primers RU-O-22929 and RU-O-24042) and Sanger sequencing (Macrogen).

To isolate knock-in 3xFLAG tagged *AGO1* Aag2 cell lines, cells were resuspended at 2E6/mL in FACS cell sorting buffer (complete L-15 supplemented with 10 mM HEPES, 5 mM EDTA and 40 ng/mL DAPI). Live, RFP-positive cells were sorted for integration of the donor template. Live, RFP negative cells were sorted post Cre transfection and excision. All sorting was performed using a BD FACSAria II sorter (BD Biosciences) with a 100 µm nozzle and a sheath pressure of ~ 20 lbf/in^2^ into 96-well plates containing 50% fresh media, and 50% conditioned media. Cells were expanded and genomic DNA was isolated and screened for integration or excision by PCR (primers RU-O-26075 and RU-O-26076); amplicons were visualized on ~ 1% agarose gels with 1 kb plus DNA ladder (NEB) and SYBR Gold (Thermo Fisher Scientific). WT DNA generates a band of ~ 1460 bp, DNA with HDR-mediated integration of the donor template generates a band of ~ 3700 bp, and Cre excised DNA generates a band of ~ 1500 bp). The same PCR was used to Sanger-sequence (Genewiz) B2 and C10 knock-in cell lines to confirm the correct sequence post-integration and post-excision.

### Editing efficiency estimates

The percent of edited clones reported was calculated by sequencing isolated clones (primers RU-O-22776 and RU-O-22777, Aag2; primers RU-O-22930 and RU-O-22931, U4.4). The number of clones that had mixed traces or deletions at sgRNA cleavage sites was calculated over the total number of clones sequenced (Aag2 = 26/93, U4.4 = 8/13).

### SDS-PAGE and immunoblot

Potentially edited clone cell pellets were collected alongside positive control cell pellets. For Aag2 cells, positive controls were Aag2 cells transfected with pKRG4-mPUB-3xFLAG-Aag2-AGO1-short-pAc or the stably transformed pKRG4-mPUb-3xFLAG-Aag2-AGO1-long-pAc Aag2 cells. For U4.4, cells were transfected with pKRG4-mPUb-3xFLAG-U44-AGO1-pAc. Pellets were lysed in ice-cold 1X PXL lysis buffer (0.1% SDS, 0.5% sodium deoxycholate, 0.5% NP-40 in 1X PBS) plus protease inhibitor (complete, Mini Protease Inhibitor Cocktail Tablets, EDTA free, Roche) on ice for 10 min and clarified by centrifugation at 15,000 rpm for 20 min at 4 °C. Supernatants were collected and total protein was determined by BCA assay (Pierce BCA Protein Assay Kit, Thermo Scientific) using a FLUOstar Omega Microplate Reader (BMG LABTECH). 10–15 µg protein/sample was diluted in 1X LDS Sample Buffer and 50 mM DTT (Sigma-Aldrich), then incubated for 10 min at 70 °C. Samples were loaded on 4–12% Bis–Tris gels and run in 1X MOPS SDS Running Buffer in the Mini Gel Tank according to the manufacturer’s protocol alongside Precision Plus Protein Dual Color Standards (Bio-Rad). Following electrophoresis, protein was transferred (Blot Module Set, Thermo Fisher Scientific) onto 0.2 µm nitrocellulose (Amersham Protran Premium, GE Healthcare) according to manufacturer’s protocol. Blots were developed using fluorescent detection (LI-COR) with the following primary antibodies and final concentrations: anti-*Drosophila* AGO1 ab5070 (1 µg/mL; Abcam), anti-*Ae. aegypti* AGO1 (1 µg/mL; generated in-house), anti-Cas9 ab204448 (1 µg/mL; Abcam), and anti-FLAG (0.5 µg/mL; monoclonal M2, Sigma-Aldrich). Following development membranes were imaged (Odyssey CLX, LI-COR).

### Luciferase reporter assays

To assay miRNA-mediated silencing in U4.4 clones, reporters were cloned corresponding to 4 × or 6 × miR-34-5p ideal target sites (8mer target followed by 4 mismatches, RU-O-24800 and RU-O-24801). Oligos were annealed and inserted into the *NotI/XhoI*-digested psiCHECK2 plasmid (Promega). 1E5 cells/well were plated in 48-well plates and transfected with empty, 4 ×, or 6 × ideal reporters. Day 2 post-transfection, cells were harvested and analyzed using the Dual-Luciferase Reporter Assay System (Promega) and a FLUOstar Omega Microplate Reader (BMG LABTECH), according to the manufacturer’s instructions. The *Renilla* luciferase (RLuc) signal in each well was normalized to that well’s firefly luciferase (FLuc) signal to measure repression. Then the RLuc/Fluc ratio was normalized to the ratio obtained with the empty reporter in each cell line (which is the unrepressed ratio for that U4.4 clone). To compare normalized ratios to U4.4 clones with WT AGO1 expression, we then divided each clone’s normalized ratio per reporter by the average ratio for that reporter obtained with our two WT clones to obtain a measurement of silencing efficiency. Transfections were performed in triplicate and data were analyzed using two-way analysis of variance (ANOVA) with Dunnett’s post hoc test, compared to the WT clone sg1 A (Prism 8).

## Supplementary Information

Supplementary Information.

## Data Availability

Unpublished, experimentally validated AGO1 transcript/protein isoforms were deposited in GenBank under accessions: MW035627-MW03529. The cell lines generated and datasets analyzed during the current study are available from the corresponding author on reasonable request. Plasmid sequences are provided in the Supplementary Information file and pKRG3 and *AGO1* HDR donor plasmids have been deposited to Addgene (plasmids 162161–162164); plasmids are also available upon request.
