# Author Correction: A selectable, plasmid-based system to generate CRISPR/Cas9 gene edited and knock-in mosquito cell lines

**DOI:** 10.1038/s41598-021-87189-9

**Published:** 2021-04-01

**Authors:** Kathryn Rozen-Gagnon, Soon Yi, Eliana Jacobson, Sasha Novack, Charles M. Rice

**Affiliations:** Laboratory of Virology and Infectious Disease, The Rockefeller University, New York, NY 10065 USA

Correction to: *Scientific Reports* 10.1038/s41598-020-80436-5, published online 12 January 2021

The Supplementary Information published with this Article contains an error in the restriction enzyme noted for sgRNA cloning on the plasmid sequences. The correct Supplementary Information file is provided below.

## Supplementary Information

Supplementary Information.

