# Supplementary material for: A selectable, plasmid-based system to generate CRISPR/Cas9 gene edited and knock-in mosquito cell lines
Source: Sci Rep. 2021 Jan 12;11:736. doi: 10.1038/s41598-020-80436-5 (PMC7804293; doi:10.1038/s41598-020-80436-5)
Supplement: Supplementary file 1 — Supplementary Information. [file 41598_2020_80436_MOESM1_ESM.pdf]

Supplementary Information for:

A selectable, plasmid-based system to generate CRISPR/Cas9 gene edited and knock-in mosquito cell lines

Kathryn Rozen-Gagnon[1*], Soon Yi[1], Eliana Jacobson[1], Sasha Novack[1], and Charles M. Rice[1]

[1]Laboratory of Virology and Infectious Disease, The Rockefeller University, New York, NY 10065, USA

*Corresponding author: Kathryn Rozen-Gagnon. Phone (212) 327-7009. Fax (212) 327-7048. krozen@rockefeller.edu

Supplementary Methods (all references are numbered as in the main text)

CRISPR/Cas9 plasmid generation

To replace the *dme* phsp70 promoter (*hsp70Bb*[47,52]) in pDCC6 with the *Ae. aegypti* polyubiquitin promoter (*aae PUb*[15,32,54]), an *AfeI* site was introduced by site-directed mutagenesis using the QuikChange II XL kit (Agilent) and oligos RU-O-22971 and RU-O-22972. The region containing the *Afe1* site was then cloned into a clean pDCC6 background by digestion of the parental pDCC6 and pDCC6-AfeI with flanking sites *SapI/AvrII* followed by ligation, transformation, and DNA isolation. *Aae* Pub with flanking *AfeI/AvrII* sites was amplified using RU-O-22977 and RU-O-22978 and Phusion polymerase (NEB) from the plasmid pSL1180-HR-PUbECFP[15,32] (a gift from Leslie Vosshall; Addgene plasmid # 47917; http://n2t.net/addgene:47917; RRID:Addgene_47917). The resulting plasmid, pKRG2 (pKRG2-dU6-PUb-3xFLAG-hSpCas9), contains the *dme* U6-2 promoter (*Drosophila* Pol III promoter *U6:96Ab*[47,48]) and the *aae* PUb promoter driving expression of hSpCas9.

To replace the *dme* U6-2 promoter with the *Aae. aegypti* U6 promoter (*aae* U6; *AAEL017774*[57]), we had to alter the sgRNA cloning sites due to an internal *BbsI* site in the *aae* U6. We designed primers to add an overhang corresponding to the *aae* U6 to a modified sgRNA cloning site that relies on *BsmBI* to the pKRG2 sgRNA tracrRNA (trans-activating CRISPR RNA) scaffold and terminator sequence, with a downstream *Afe*I site (RU-O-22974 and RU-O-22975). The scaffold PCR and a gBlocks Gene Fragment containing the *aae* U6 sequence and an upstream *SacI* site were assembled by Gibson assembly. The assembled DNA was PCR amplified using primers RU-O-22975 and RU-O-22976. The *aae* U6 insert and pKRG2 were digested with *SacI/AfeI*, ligated, and DNA was isolated to obtain pKRG3-mU6-PUb-3xFLAG-hSpCas9, which contains the *aae* U6 promoter driving sgRNA expression and the *aae* PUb promoter driving expression of hSpCas9.

We additionally generated a version of this plasmid with the 3xFLAG at the beginning of the Cas9 removed. To remove the 3xFLAG, we introduced a *NcoI* site by site-directed mutagenesis using oligos RU-O-23100 and RU-O-23101. We then cloned this mutagenized insert into a clean pKRG3 background by restriction enzyme digest with *BglII/XhoI*. The pKRG3-NcoI plasmid was then digested with *NcoI* to remove the 3xFLAG and re-ligated to generate pKRG3-mU6-PUb-hSpCas9. We made another variation with the puromycin resistance cassette (pAc) added. We amplified the end of Cas9, the intervening T2A sequence[59], and pAc from pAc-sgRNA-Cas9[30] (a gift from Ji-Long Liu; Addgene plasmid #49330; http://n2t.net/addgene:49330; RRID:Addgene_49330) using primers RU-O-23782 and RU-O-23783. We then digested pKRG3 with *Eag1/BsrGI* and generated pKRG3-mU6-PUb-hSpCas9-pAc by Gibson assembly. For comparative purposes, we also removed the 3xFLAG and added the pAc to hSpCas9 in pKRG3 by the same method, generating pKRG2-dU6-PUb-hSpCas9-pAc.

3xFLAG-tagged AGO1 plasmid generation

To generate overexpression plasmids as positive controls for mosquito AGO1 immunoblotting, the pKRG3 plasmid was further modified. Aag2 N-terminal 3xFLAG-tagged short and long AGO1 isoforms and the U4.4 N-terminal 3xFLAG-tagged AGO1 with pKRG3 plasmid overhangs were PCR-amplified from an in-house plasmid containing experimentally validated AGO1 sequences in each cell line (unpublished data). The hSpCas9 sequence was removed from pKRG3-mU6-PUb-hSpCas9-pAc by digestion with *NcoI/BsrGI* and AGO1 sequences were inserted by Gibson assembly. Alternatively, to generate an empty pKRG3 plasmid, the ends of the *NcoI/BsrGI*-digested plasmid were filled with T4 DNA polymerase (NEB) according to the manufacturer's protocol and blunt ends were re-ligated. Finally, the *aae* U6 sequence was removed from pKRG3 Ago-containing or empty plasmids by digestion with *SapI/AfeII*; the ends were filled with T4 DNA polymerase and blunt ends were ligated. This generated an empty, all-purpose pKRG4-mPUb-pAc plasmid, as well as pKRG4-mPUb-3xFLAG-Aag2-AGO1-short-pAc, pKRG4-mPUb-3xFLAG-Aag2-AGO1-long-pAc, and pKRG4-mPUb-3xFLAG-U44-AGO1-pAc. To express Cre recombinase for excision of the fluorescent reporter between the loxP sites, Cre recombinase was amplified from pME66 (a gift from S. Sarbanes) using primers adding *SacI/AvrII* sites; the Cre insert and pKRG4-mPUb-pAc were digested with *SacI/AvrII* to generate pKRG4-mPUb-Cre-pAc.

CRISPR guide RNA design and cloning

We designed CRISPR RNAs (crRNAs) corresponding to *AGO1*. For Aag2 cells, we used the *Ae. aegypti* AaegL3 genome assembly, AaegL3.3 annotations, *AAEL012410* (at the time of the design this was the most updated

assembly; since that time the AaegL5 genome assembly has been released[23]; the *AGO1* gene ID remains the same and the updated AaegL5.2 gene annotation contains the correct *AGO1* transcriptional start site). For U4.4 cells, we used the *Ae. albopictus* AaloF1 assembly, AaloF1.2 annotations, *AALF020776.* We first confirmed the genomic sequence around the experimentally determined translational start site in each cell line (unpublished data from 5'RACE and cDNA sequencing). Aag2 and U4.4 cell genomic DNA was isolated using the DNeasy Blood & Tissue Kit (Qiagen) according to the manufacturer's protocol. Aag2 genomic DNA was amplified using primers RU-O-22776 and RU-O-22777, designed using the *Ae. aegypti* AaegL3 assembly, which has the correct annotated starting methionine. U4.4 genomic DNA was amplified using primers RU-O-22929 and RU-O-22931, designed using the *Ae. albopictus* AaloF1 assembly, where we could only identify a downstream methionine in 5'RACE experiments. Three guide oligos containing the *BsmBI* overhangs in pKRG3 plasmids were designed for each cell line based on protospacer adjacent motif (PAM) NGG sequences in close proximity to the starting methionine (RU-O-23427 to RU-O-23434, *Ae. aegypti*; RU-O-23456 to RU-O-23463, *Ae. albopictus*). The parent pKRG3-mU6-PUb-hSpCas9-pAc plasmid was digested with *BsmBI* and annealed oligos were ligated to generate 6 pKRG3 plasmids, one for each guide, according to protocols from Kistler et al., 2015 and Cornell's Stem Cell and Transgenic Core Facility (https://transgenics.vertebrategenomics.cornell.edu/genome-editing.html). These co-express the crRNA plus the tracrRNA as a single guide RNA (sgRNA), and hSpCas9.

Cloning of homology-directed repair (HDR) donor template

The pSL1180-HR-PUbECFP plasmid was used as the backbone for cloning an HDR donor template. A 2kb homology arm fragment around the translational start site of *AGO1* in Aag2 cells was amplified from Aag2 genomic DNA using oligos RU-O-24703 and RU-O-24704, to add homology with the pSL1180-HR-PUbECFP plasmid. We ordered a gBlocks Gene Fragment containing an inserted 3xFLAG-tag between the first methionine and the second amino acid of AGO1, with silent mutations to ablate the sgRNA PAM sites. The gBlock extended past *PpuMI/EagI* sites in the homology arm. Next, the homology arm was digested with *PpuMI/EagI* to drop out the central ~160 nt, generating 2 ~1kb homology fragments overlapping the gBlock. pSL1180-HR-PUbECFP was digested with *NotI/EcoRI*, dropping out the PUB-eCFP, and the fragments were assembled to generate the intermediate plasmid pSL1180-HR-Aag2-3xFLAG-AGO1. Next, pSL1180-HR-Aag2-3xFLAG-AGO1 was modified to add the loxp-PUb-RFP-loxP cassette, with overlaps corresponding to the upstream homology arm and downstream 3xFLAG-AGO1 sequence. We generated four PCRs: PCR1) 5'HA-loxP primers = RU-O-25019 and RU-O-25020, template = pSL1180-HR-Aag2-3xFLAG-AGO1 pSL1180-HR-Aag2-3xFLAG-AGO1); PCR2: loxP-PUB-RFP (primers= RU-O-25021 and RU-O-25022, template = pKRG3), PUb-RFP-loxP (primers = RU-O-25023 and RU-O-25024, template= pTRIPZ; Dharmacon), loxP-3xFLAG-3'HA (primers = RU-O-25027 and RU-O-25028, template = pSL1180-HR-Aag2-3xFLAG-AGO1). pSL1180-HR-Aag2-3xFLAG-AGO1 was digested with *KpnI/PpuMI* and the fragments were assembled by Gibson assembly to generate pSL1180-HR-Aag2-loxP-PUb-RFP-loxP-3xFLAG-AGO1.

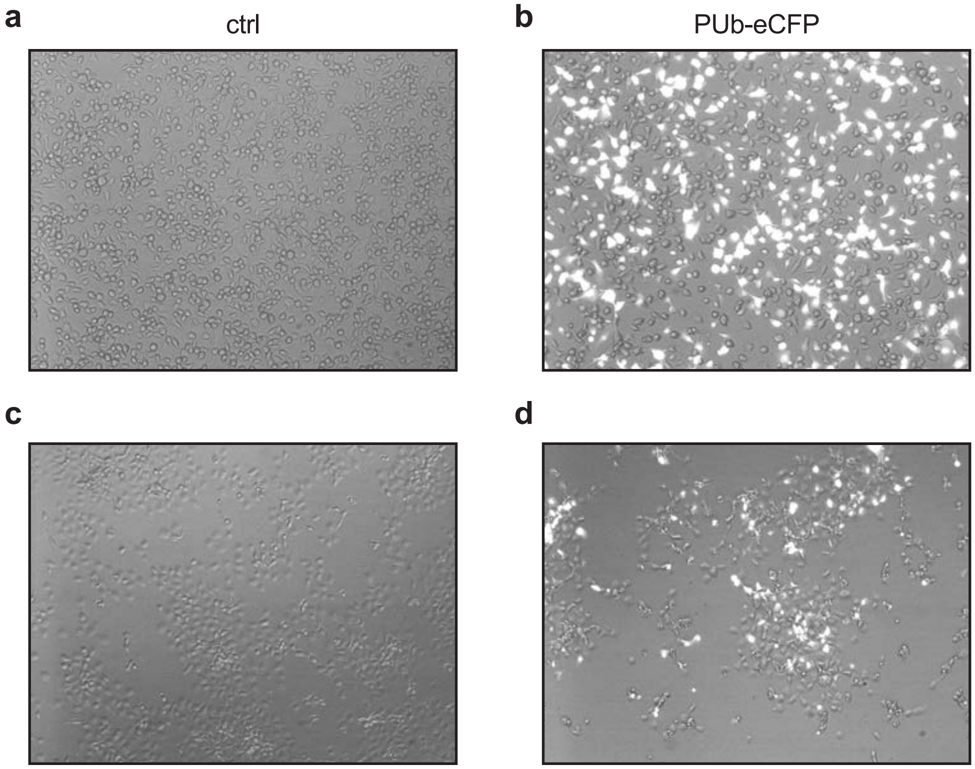

Supplementary Figure 1. Efficient transfection of mosquito cells.

(a) Representative merged brightfield and enhanced cyan flourescent protein (eCFP) image for control (ctrl) U4.4 cells treated with transfection reagent alone.

(b) As in (a), for U4.4 cells transfected with PUb-eCFP.

(c) As in (a), for Aag2 cells.

(d) As in (b), for Aag2 cells.

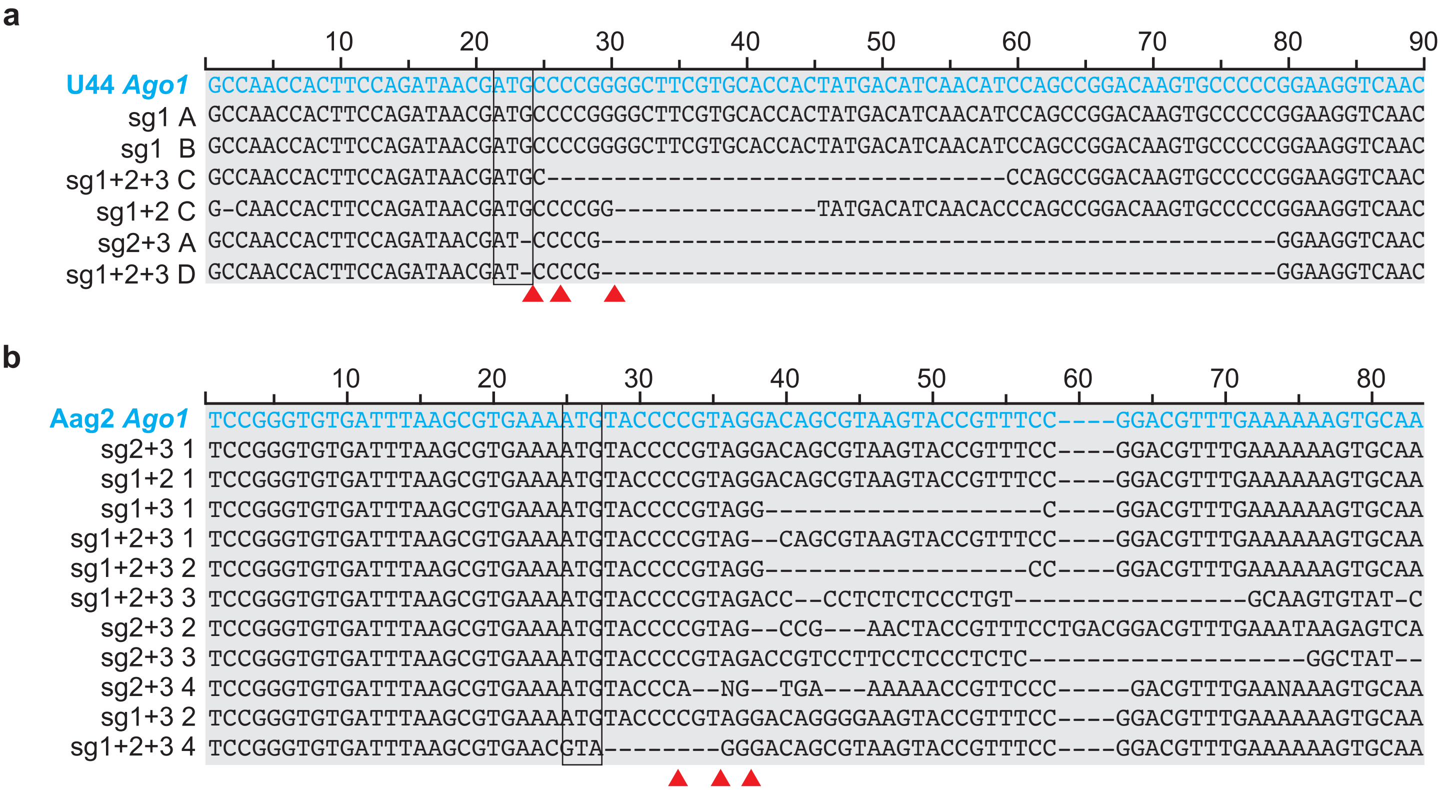

Supplementary Figure 2. *AGO1* sequences of single cell clones isolated after CRISPR/Cas9 transfection.

(a) Representative alignment of *AGO1* sequences from established U4.4 single cell clones. Clones were isolated and sequenced post-transfection with pKRG3 CRISPR/Cas9 plasmids containing guides targeting *AGO1*. sgRNA cleavage sites = red arrows; starting methionine = black box; reference sequence shown in blue.

(b) As in (a), for Aag2 cells.

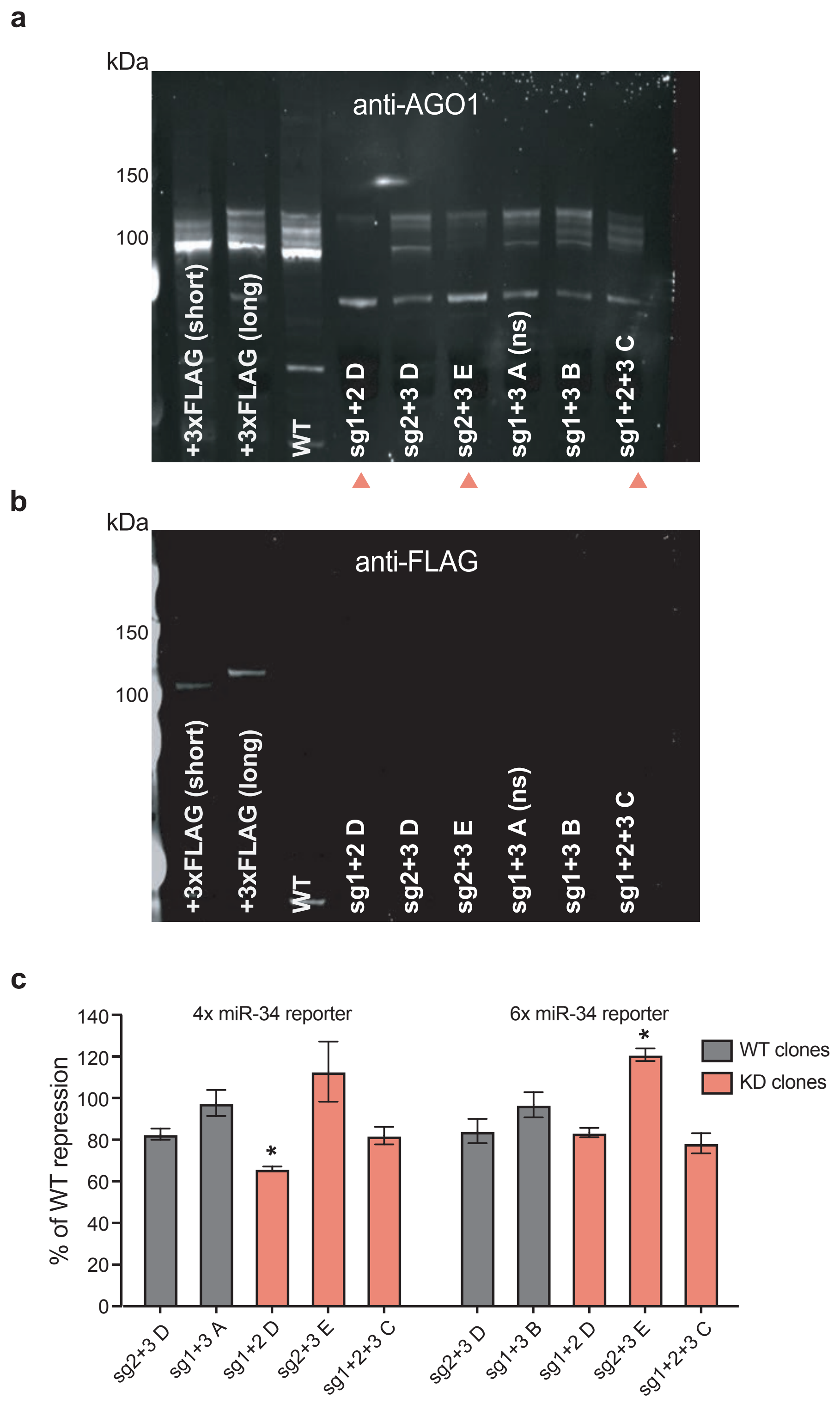

Supplementary Figure 3. AGO1 protein levels and function in Aag2 clones.

(a) Immunoblot of AGO1 showed Aag2 clones with wild-type (WT) and reduced (salmon arrows) AGO1 protein levels; A-E denote clones obtained from each sgRNA singly or in combination; ns = not shown (in reporter assay).

(b) 3xFLAG-tagged Aag2 AGO1 short and long isoforms were expressed and were detected by both the anti-AGO1 antibody in (a) and the anti-FLAG antibody.

(c) Luciferase reporter assay measuring miR-34 mediated repression of 4 or 6 repeated miR-34 sites (4x, 6x miR-34 reporter). Normalization was performed as in Fig. 3e. The percent (%) of repression compared to WT clones sg2+3 D and sg1+3 A is shown. $P < 0.0001$ (overall ANOVA comparing different clones); individual groups were compared using the Dunnett's *post hoc* test compared to the WT clone sg1+3 B; $^*P < 0.05$.

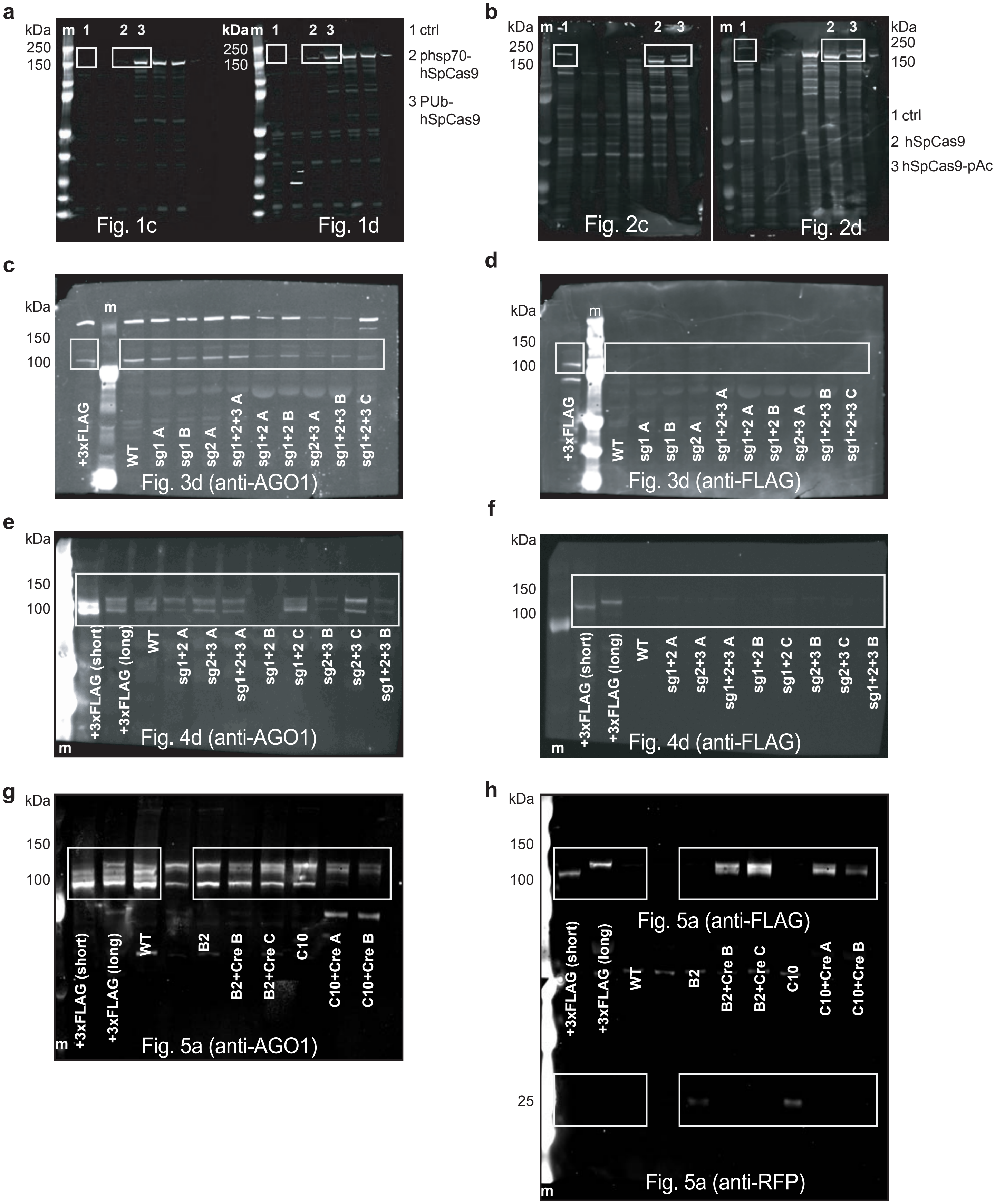

Supplementary Figure 4. Full-length blots.

(a-h) Full-length blots for all Figures. Regions included in Figures are indicated with boxes; m = marker, kDa = kilodaltons.

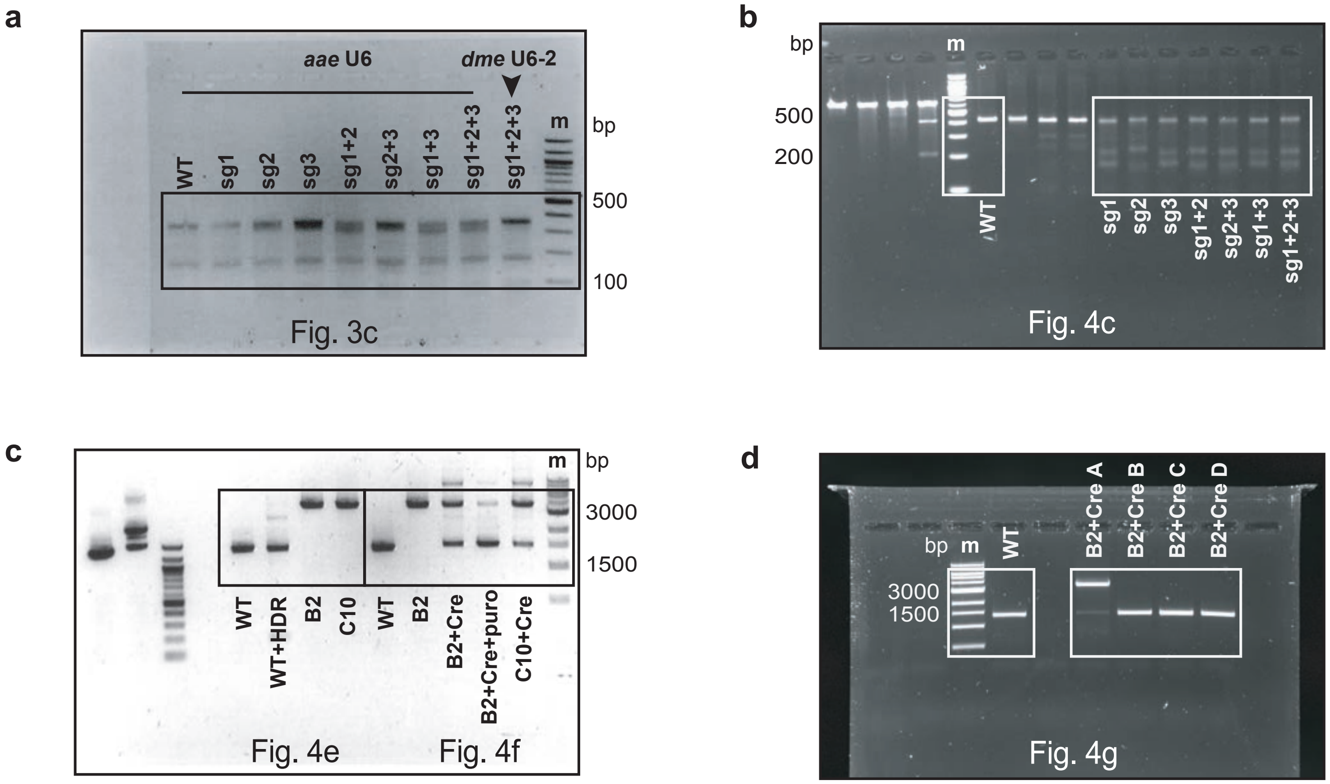

Supplementary Figure 5. Full-length gels.

(a-d) Full-length gels for all Figures. Regions included in Figures are indicated with boxes; m = marker, bp = base pairs.

Supplementary Tables

Supplementary Table 1. Sequences of all oligos used in this study.

Oligo ID	Sequence	Purpose
pKRG cloning		
RU-O-22971	CTGCAGAATTGGCGCAAGCGCTAAAAACGGACT	introduce *AfeI* pDCC6 forward; PAGE-purified
RU-O-22972	AGTCCGTTTTTAGCGCTTGCGCCAATTCTGCAG	introduce *AfeI* pDCC6 reverse; PAGE-purified
RU-O-22977	GACAGCGCTTGCGCCAATTCTGCAGACAAATGGCTATCTTTACATGTAGCTTGTGC<br>ATTG	PUb promoter PCR forward
RU-O-22978	GTCCCTAGGTGTATACCTCCGGAAGCGCGCACTCGAGATTCGAACAAGCTTATCGA<br>GCTTGGTGTTGAAATCTCTGTTGAGC	PUb promoter PCR reverse
RU-O-22974	GACGAAGACTATATAAGAGCAGAGGCAAGAGTAGTGAAATGGAGACGACGTCTCTG<br>TTTTAGAGCTAGAAATAGC	tracr RNA scaffold PCR forward
RU-O-22975	GCAGAATTGGCGCAAGCGCTGTC	tracr RNA scaffold & assemble *aae* U6/scaffold PCR reverse
RU-O-22976	GACGAAGAGCGCCCAATACGCAA	assemble aae U6/scaffold PCR forward
gBLOCK *aae* U6	GAAGAGCGCCCAATACGCAAACCGCCTCTCCCCGCGGGTTGGCCGATTCATTAATG<br>CAGGCAACTCGTGAAAGGTAGGCGGATCAGCGAATGAAATCGCCCATCGAGTTGAT<br>ACGTCCATCCATCGCTAGAACCGCGTTCGCTGTAGAAGACTATATAAGAGCAGAGG<br>CAAGAGTAGTGAAATGGAGACG	U6 geneblock
RU-O-23101	GATATTGATTACAAAGACGATGACGATACCATGGCCCCAAAGAAG	introduce *NcoI* remove 3xFLAG forward; PAGE-purified
RU-O-23100	CTTCTTTGGGGCCATGGTATCGTCATCGTCTTTGTAATCAATATC	introduce *NcoI* remove 3xFLAG reverse; PAGE-purified
RU-O-25485	GACCCTAGGATGGGCCCAAAGAAG	Cre into pKRG4 forward
RU-O-25486	CGGTAGAGCTCATCGCCATCTTCCAG	Cre into pKRG4 reverse
sgRNA oligos		
RU-O-23427	AAATGGTACTTACGCTGTCCTACG	pKRG Aag2 Ago1 sgRNA 1 forward
RU-O-23428	AAACCGTAGGACAGCGTAAGTACC	pKRG Aag2 Ago1 sgRNA 1 reverse
RU-O-23430	AAATGAGCGTGAAAATGTACCCCGT	pKRG Aag2 Ago1 sgRNA 2 forward
RU-O-23431	AAACACGGGGTACATTTTCACGCTC	pKRG Aag2 Ago1 sgRNA 2 reverse
RU-O-23433	AAATGACGGTACTTACGCTGTCCTA	pKRG Aag2 Ago1 sgRNA 3 forward
RU-O-23434	AAACTAGGACAGCGTAAGTACCGTC	pKRG Aag2 Ago1 sgRNA 3 reverse
RU-O-23456	AAATGTAGTGGTGCACGAAGCCCCG	pKRG U4.4 Ago1 sgRNA 1 forward
RU-O-23457	AAACCGGGGCTTCGTGCACCACTAC	pKRG U4.4 Ago1 sgRNA 1 reverse
RU-O-23459	AAATGTTCCAGATAACGATGCCCCG	pKRG U4.4 Ago1 sgRNA 2 forward
RU-O-23460	AAACCGGGGCATCGTTATCTGGAAC	pKRG U4.4 Ago1 sgRNA 2 reverse
RU-O-23462	AAATGACTTCCAGATAACGATGCCC	pKRG U4.4 Ago1 sgRNA 3 forward
RU-O-23463	AAACGGGCATCGTTATCTGGAAGTC	pKRG U4.4 Ago1 sgRNA 3 reverse
HDR donor template oligos		
RU-O-24703	GACCATGATTACGAATTCGACATGTAGGACATGTGGGG	Aag2 HA PCR forward
RU-O-24704	CCAGCTGCAGGCGGCCGCCACCGGATTTGCTTTTCGTC	Aag2 HA PCR reverse
gBLOCK Aag2 HDR donor template	CGAAAAGTGCAAAAATTCGGCCGTAATTAGTTCGCTTCCGTTTTTCCGAGTGCCTC<br>CGATCGTTAGCGGACCGTTCCGGGTGTGATTTAAGCGTGAAAATGGATTACAAGGA<br>TCACGATGGAGATTACAAGGATCACGATATCGATTACAAGGATGATGATAAGTATC<br>CGGTGGGTCAACGTAAGTACCGTTTCCGGACGTTTGAAAAAGTGCAATTCACAAG<br>AGAAGAAAAAAAAAGTGTTGACGAGGGGACCTTCCTTCCTCCCTC	
RU-O-25019	GGAGCGTCCGTAAAATGAAA	*Kpn1* HA overlap forward
RU-O-25020	ATAACTTCGTATAGCATACATTATACGAAGTTATTTTCACGCTTAAATC	5'HA add loxP reverse
RU-O-25021	GCTATACGAAGTTATTATCTTTACATGTAG	loxP-PUb forward
RU-O-25022	GATCAGCTCGCTCATGCGGCCGCGGTAGAGCTCCGAATTCAC	pUB-RFP reverse
RU-O-25023	ATGAGCGAGCTGATC	RFP forward
RU-O-25024	CATTATACGAAGTTATTAATTAATTATCTGTGCCCCAG	RFP-loxP reverse
RU-O-25027	ATAACTTCGTATAATGTATGCTATACGAAGTTATATGGATTACAAGGATC	loxP-HA forward
RU-O-25028	AGAGACGAGAGGGAGGAAGG	HA *PpuMI* overlap reverse
Sequencing, surveyor & integration PCRs		
RU-O-26075	ACTTGTCTCACTCGCCATCATACG	Aag2 HDR PCR F
RU-O-26076	CGATCAGATGCGCACGAAAACT	Aag2 HDR PCR R
RU-O-22776	ACTTGTCTCACTCGCCATCA	Aag2 surveyor/sequencing forward
RU-O-22777	GTCCGTACACAGGAAAAGCC	Aag2 surveyor/sequencing reverse
RU-O-22929	GCCAACCACTTCCAGATAACG	U4.4 surveyor forward
RU-O-24042	AGGATGGCATCGTACGGAAT	U4.4 surveyor reverse
RU-O-22930	TCTACCGGTGTTCACCTGTC	U4.4 sequencing forward
RU-O-22931	TGATCTCCCGGTTGACCTTC	U4.4 sequencing reverse
Reporter cloning oligos		
RU-O-24800	TCGAGCAACCAGCTAGGCCACTGCCACCGGCAACCAGCTAGGCCACTGCCACCGGC<br>AACCAGCTAGGCCACTGCCACCGGCAACCAGCTAGGCCACTGCCAGC	Aag2 miR-34-5p 4x ideal psiCHECK2 forward
RU-O-24801	GGCCGCTGGCAGTGGCCTAGCTGGTTGCCGGTGGCAGTGGCCTAGCTGGTTGCCGG<br>TGGCAGTGGCCTAGCTGGTTGCCGGTGGCAGTGGCCTAGCTGGTTGC	Aag2 miR-34-5p 4x ideal psiCHECK2 reverse
RU-O-24794	TCGAGCAACCAGCTAACCACACTGCCAGC	Aag2 miR-34-5p perfect psiCHECK2 forward
RU-O-24795	GGCCGCTGGCAGTGTGGTTAGCTGGTTGC	Aag2 miR-34-5p perfect psiCHECK2 reverse

HA = homology arm; HDR = homology-directed repair; tracr = trans-activating CRISPR; sgRNA = single-guide RNA.
All oligos were ordered in standard desalted format from IDT, unless indicated otherwise.

Plasmid Sequences

pKRG3-mU6-PUb-3xFLAG-hSpCas9

aae U6
BbsI sites for sgRNA cloning
sgRNA tracrRNA and U6 terminator
aae PUb promoter
3xFLAG
NLS
hSpCas9

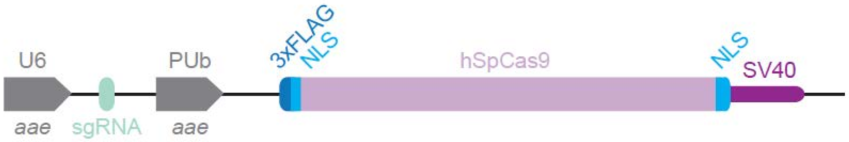

GCGCCCAATACGCAAACCGCCTCTCCCCGCGGGTTGGCCGATTCATTAATGCAGGCAACTCGTGAAAGGTAGGCGGATCAGCGAATGAA
ATCGCCCATCGAGTTGATACGTCCATCCATCGCTAGAACCGCGTTCGCTGTAGAAGACTATATAAGAGCAGAGGCAAGAGTAGTGAAAT
gGAGACGaCGTCTCtGTTTTAGAGCTAGAAATAGCAAGTTAAAATAAGGCTAGTCCGTTATCAACTTGAAAAAGTGGCACCGAGTCGGT
GCTTTTTTGTTTTAGAGCTAGAAATAGCAAGTTAAAATAAGGCTAGTCCGTTTTTAGCGCTTGCGCCAATTCTGCAGACAAATGGCTAT
CTTTACATGTAGCTTGTGCATTGAATCCAATTATAATTTGCCTTGGCACCAGCTGAGCCAGACAAGAAAGAAAGCTTCCCAGAAGTATA
TCGATTTAGAAGGGTTGACGTCACTTGCTGACTGCACTAATACAGCAAATGATGCAATTAGAATGATTCAAGTGAAATTCCCAAATTAC
TGATTTTTCTCTGGATTTGGTTATCAGATTACATTCGAAGCTAAGATTAGCTACCGAAATTGTCGATCAAATCAGGAAATCCTTTCTCT
ATCGAAAAAGGCATTCGCACATCTTCCTCTCTATGCCATATACACGAAGGGTAGGTACATTGACGTCTTTGCCAGAAGTTGAACTGCAT
CGTTCAAGGTACAGAATGAACGACTAACAGACACAAGCACGTTTTGCTGTCCATTCAGACACAGGGATGGTACCCATATTCGATCGATA
TAGAGCCATCCAACCGAACAGAGGTATATGTATGAATGTATTGCTGAAATTTTCTAGAAGTACAACCACCACTACGACAGTGTCTATAA
AACGCCCCTGCAAAGGCGAAACCAGCTCAATCGAATACGTTTCCTAGTGGAGTGAACATTACGCGGCCCAAGTAAGCAGTGCCAGTGCA
AGTGAAGTGAAGTCTCTAGTGAAAAAGAGTGATCCAATTAGCCAGAGGAGAAAATTTCAGAGTGAACAAAGCTTTATTCAAAGGACAAT
TACTATTAAATTGGTGAAAGTGCATTTCGGTGAAGGGAATCTTCTAGTGAAGGTAGGTAAATTAATTGATGAAATTATAGCTATGAGCG
AAAACTAGTTTGGTGAATGATTCCTTTGTCTTTGAATGAGCAAACTATTTTCCAAGATGGCGACTATTGAGCTTTGAGTGATTAGTGAA
AATTTGCAACGCAGTTTCATCATCATTGATAAAACCCAATTGTGATTCACAGCGATAATCATATTTCGTTGAATCATCGCTACTAATTG
AATTAAATTTCTAGAATAATAAGAATAACGTATTTGCTCCGTCACATATCTAAAATAAATATTTTGATGGTTAATTACCCATTAAGGTA
ATATTAACACATATCGAGAAAAACCTTGAGGAAATCGTGAAAACTTGAAGATACGCAATTTCAAAACTACGTAGTTCAAAGTCGAAAAC
AAGTTAATTTTTCACTTAAAAGTAGGGCGTTGTTGTGACGTCATCACCTTCAAGTGTATATTTTTCACTTGGCCTGCGACTGCAAACGC
AGACAAAGCAAAACAAGTTTAAAACCTGTCGTGTCGTGCTCGAAGCCAAAGGCAATGAATCAATATCAAATGAGAGTTTGCATTTCACA
ACCAATTACTCAAGCGTTTCCTCGTTTCTTTTTCTGCTCAACAGAGATTTCAACACCAAGCTCGATAAGCTTGTTCGAATCTCGAGTGC
GCGCTTCCGGAGGTATACACCTAGGCGGTACCACTGCAGTGAATTCGGAGCTCTACCGGTGCCACCATGGACTATAAGGACCACGACGG
AGACTACAAGGATCATGATATTGATTACAAAGACGATGACGATAAGATGGCCCCAAAGAAGAAGCGGAAGGTCGGTATCCACGGAGTCC
CAGCAGCCGACAAGAAGTACAGCATCGGCCTGGACATCGGCACCAACTCTGTGGGCTGGGCCGTGATCACCGACGAGTACAAGGTGCCC
AGCAAGAAATTCAAGGTGCTGGGCAACACCGACCGGCACAGCATCAAGAAGAACCTGATCGGAGCCCTGCTGTTCGACAGCGGCGAAAC
AGCCGAGGCCACCCGGCTGAAGAGAACCGCCAGAAGAAGATACACCAGACGGAAGAACCGGATCTGCTATCTGCAAGAGATCTTCAGCA
ACGAGATGGCCAAGGTGGACGACAGCTTCTTCCACAGACTGGAAGAGTCCTTCCTGGTGGAAGAGGATAAGAAGCACGAGCGGCACCCC
ATCTTCGGCAACATCGTGGACGAGGTGGCCTACCACGAGAAGTACCCCACCATCTACCACCTGAGAAAGAAACTGGTGGACAGCACCGA
CAAGGCCGACCTGCGGCTGATCTATCTGGCCCTGGCCCACATGATCAAGTTCCGGGGCCACTTCCTGATCGAGGGCGACCTGAACCCCG
ACAACAGCGACGTGGACAAGCTGTTCATCCAGCTGGTGCAGACCTACAACCAGCTGTTCGAGGAAAACCCCATCAACGCCAGCGGCGTG
GACGCCAAGGCCATCCTGTCTGCCAGACTGAGCAAGAGCAGACGGCTGGAAAATCTGATCGCCCAGCTGCCCGGCGAGAAGAAGAATGG
CCTGTTCGGAAACCTGATTGCCCTGAGCCTGGGCCTGACCCCCAACTTCAAGAGCAACTTCGACCTGGCCGAGGATGCCAAACTGCAGC
TGAGCAAGGACACCTACGACGACGACCTGGACAACCTGCTGGCCCAGATCGGCGACCAGTACGCCGACCTGTTTCTGGCCGCCAAGAAC
CTGTCCGACGCCATCCTGCTGAGCGACATCCTGAGAGTGAACACCGAGATCACCAAGGCCCCCCTGAGCGCCTCTATGATCAAGAGATA
CGACGAGCACCACCAGGACCTGACCCTGCTGAAAGCTCTCGTGCGGCAGCAGCTGCCTGAGAAGTACAAAGAGATTTTCTTCGACCAGA
GCAAGAACGGCTACGCCGGCTACATTGACGGCGGAGCCAGCCAGGAAGAGTTCTACAAGTTCATCAAGCCCATCCTGGAAAAGATGGAC
GGCACCGAGGAACTGCTCGTGAAGCTGAACAGAGAGGACCTGCTGCGGAAGCAGCGGACCTTCGACAACGGCAGCATCCCCCACCAGAT
CCACCTGGGAGAGCTGCACGCCATTCTGCGGCGGCAGGAAGATTTTTACCCATTCCTGAAGGACAACCGGGAAAAGATCGAGAAGATCC
TGACCTTCCGCATCCCCTACTACGTGGGCCCTCTGGCCAGGGGAAACAGCAGATTCGCCTGGATGACCAGAAAGAGCGAGGAAACCATC
ACCCCCTGGAACTTCGAGGAAGTGGTGGACAAGGGCGCTTCCGCCCAGAGCTTCATCGAGCGGATGACCAACTTCGATAAGAACCTGCC
CAACGAGAAGGTGCTGCCCAAGCACAGCCTGCTGTACGAGTACTTCACCGTGTATAACGAGCTGACCAAAGTGAAATACGTGACCGAGG
GAATGAGAAAGCCCGCCTTCCTGAGCGGCGAGCAGAAAAAGGCCATCGTGGACCTGCTGTTCAAGACCAACCGGAAAGTGACCGTGAAG
CAGCTGAAAGAGGACTACTTCAAGAAAATCGAGTGCTTCGACTCCGTGGAAATCTCCGGCGTGGAAGATCGGTTCAACGCCTCCCTGGG
CACATACCACGATCTGCTGAAAATTATCAAGGACAAGGACTTCCTGGACAATGAGGAAAACGAGGACATTCTGGAAGATATCGTGCTGA
CCCTGACACTGTTTGAGGACAGAGAGATGATCGAGGAACGGCTGAAAACCTATGCCCACCTGTTCGACGACAAAGTGATGAAGCAGCTG
AAGCGGCGGAGATACACCGGCTGGGGCAGGCTGAGCCGGAAGCTGATCAACGGCATCCGGGACAAGCAGTCCGGCAAGACAATCCTGGA
TTTCCTGAAGTCCGACGGCTTCGCCAACAGAAACTTCATGCAGCTGATCCACGACGACAGCCTGACCTTTAAAGAGGACATCCAGAAAG
CCCAGGTGTCCGGCCAGGGCGATAGCCTGCACGAGCACATTGCCAATCTGGCCGGCAGCCCCGCCATTAAGAAGGGCATCCTGCAGACA
GTGAAGGTGGTGGACGAGCTCGTGAAAGTGATGGGCCGGCACAAGCCCGAGAACATCGTGATCGAAATGGCCAGAGAGAACCAGACCAC
CCAGAAGGGACAGAAGAACAGCCGCGAGAGAATGAAGCGGATCGAAGAGGGCATCAAAGAGCTGGGCAGCCAGATCCTGAAAGAACACC
CCGTGGAAAACACCCAGCTGCAGAACGAGAAGCTGTACCTGTACTACCTGCAGAATGGGCGGGATATGTACGTGGACCAGGAACTGGAC
ATCAACCGGCTGTCCGACTACGATGTGGACCATATCGTGCCTCAGAGCTTTCTGAAGGACGACTCCATCGACAACAAGGTGCTGACCAG
AAGCGACAAGAACCGGGGCAAGAGCGACAACGTGCCCTCCGAAGAGGTCGTGAAGAAGATGAAGAACTACTGGCGGCAGCTGCTGAACG
CCAAGCTGATTACCCAGAGAAAGTTCGACAATCTGACCAAGGCCGAGAGAGGCGGCCTGAGCGAACTGGATAAGGCCGGCTTCATCAAG
AGACAGCTGGTGGAAACCCGGCAGATCACAAAGCACGTGGCACAGATCCTGGACTCCCGGATGAACACTAAGTACGACGAGAATGACAA
GCTGATCCGGGAAGTGAAAGTGATCACCCTGAAGTCCAAGCTGGTGTCCGATTTCCGGAAGGATTTCCAGTTTTACAAAGTGCGCGAGA

TCAACAACTACCACCACGCCCACGACGCCTACCTGAACGCCGTCGTGGGAACCGCCCTGATCAAAAAGTACCCTAAGCTGGAAAGCGAG
TTCGTGTACGGCGACTACAAGGTGTACGACGTGCGGAAGATGATCGCCAAGAGCGAGCAGGAAATCGGCAAGGCTACCGCCAAGTACTT
CTTCTACAGCAACATCATGAACTTTTTCAAGACCGAGATTACCCTGGCCAACGGCGAGATCCGGAAGCGGCCTCTGATCGAGACAAACG
GCGAAACCGGGGAGATCGTGTGGGATAAGGGCCGGGATTTTGCCACCGTGCGGAAAGTGCTGAGCATGCCCCAAGTGAATATCGTGAAA
AAGACCGAGGTGCAGACAGGCGGCTTCAGCAAAGAGTCTATCCTGCCCAAGAGGAACAGCGATAAGCTGATCGCCAGAAAGAAGGACTG
GGACCCTAAGAAGTACGGCGGCTTCGACAGCCCCACCGTGGCCTATTCTGTGCTGGTGGTGGCCAAAGTGGAAAAGGGCAAGTCCAAGA
AACTGAAGAGTGTGAAAGAGCTGCTGGGGATCACCATCATGGAAAGAAGCAGCTTCGAGAAGAATCCCATCGACTTTCTGGAAGCCAAG
GGCTACAAAGAAGTGAAAAAGGACCTGATCATCAAGCTGCCTAAGTACTCCCTGTTCGAGCTGGAAAACGGCCGGAAGAGAATGCTGGC
CTCTGCCGGCGAACTGCAGAAGGGAAACGAACTGGCCCTGCCCTCCAAATATGTGAACTTCCTGTACCTGGCCAGCCACTATGAGAAGC
TGAAGGGCTCCCCCGAGGATAATGAGCAGAAACAGCTGTTTGTGGAACAGCACAAGCACTACCTGGACGAGATCATCGAGCAGATCAGC
GAGTTCTCCAAGAGAGTGATCCTGGCCGACGCTAATCTGGACAAAGTGCTGTCCGCCTACAACAAGCACCGGGATAAGCCCATCAGAGA
GCAGGCCaAGAATATCATCCACCTGTTTACCCTGACCAATCTGGGAGCCCCTGCCGCCTTCAAGTACTTTGACACCACCATCGACCGGA
AGAGGTACACCAGCACCAAAGAGGTGCTGGACGCCACCCTGATCCACCAGAGCATCACCGGCCTGTACGAGACACGGATCGACCTGTCT
CAGCTGGGAGGCGACAGCCCCAAGAAGAAGAGAAAGGTGGAGGCCAGCTAATAGGACCCAGCTTTCTTGTACAAAGTGGTGACGTAAGC
TAGCAGGATCTTTGTGAAGGAACCTTACTTCTGTGGTGTGACATAATTGGACAAACTACCTACAGAGATTTAAAGCTCTAAGGTAAATA
TAAAATTTTTAAGTGTATAATGTGTTAAACTACTGATTCTAATTGTTTGTGTATTTTAGATTCCAACCTATGGAACTGATGAATGGGAG
CAGTGGTGGAATGCCTTTAATGAGGAAAACCTGTTTTGCTCAGAAGAAATGCCATCTAGTGATGATGAGGCTACTGCTGACTCTCAACA
TTCTACTCCTCCAAAAAAGAAGAGAAAGGTAGttGACCCCAAGGACTTTCCTTCAGAATTGCTAAGTTTTTTGAGTCATGCTGTGTTTA
GTAATAGAACTCTTGCTTGCTTTGCTATTTACACCACAAAGGAAAAAGCTGCACTGCTATACAAGAAAATTATGGAAAAATATTCTGTA
ACCTTTATAAGTAGGCATAACAGTTATAATCATAACATACTGTTTTTTCTTACTCCACACAGGCATAGAGTGTCTGCTATTAATAACTA
TGCTCAAAAATTGTGTACCTTTAGCTTTTTAATTTGTAAAGGGGTTAATAAGGAATATTTGATGTATAGTGCCTTGACTAGAGATCATA
ATCAGCCATACCACATTTGTAGAGGTTTTACTTGCTTTAAAAAACCTCCCACACCTCCCCCTGAACCTGAAACATAAAATGAATGCAAT
TGTTGTTGTTAACTTGTTTATTGCAGCTTATAATGGTTACAAATAAAGCAATAGCATCACAAATTTCACAAATAAAGCATTTTTTTCAC
TGCATTCTAGTTGTGGTTTGTCCAAACTCATCAATGTATCTTATCATGTCTGGATCCCGTTTAAACTACGCGTAATTCAAACAGGGTTC
TGGCGTCGTTCTCGTACTGTTTTCCCCAGGCCAGTGCTTTAGCGTTATTGAAAAAGGAAGAGTATGAGTATTCAACATTTCCGTGTCGC
CCTTATTCCCTTTTTTGCGGCATTTTGCCTTCCTGTTTTTGCTCACCCAGAAACGCTGGTGAAAGTAAAAGATGCTGAAGATCAGTTGG
GTGCACGAGTGGGTTACATCGAACTGGATCTCAACAGCGGTAAGATCCTTGAGAGTTTTCGCCCCGAAGAACGTTTTCCAATGATGAGC
ACTTTTAAAGTTCTGCTATGTGGCGCGGTATTATCCCGTATTGACGCCGGGCAAGAGCAACTCGGTCGCCGCATACACTATTCTCAGAA
TGACTTGGTTGAGTACTCACCAGTCACAGAAAAGCATCTTACGGATGGCATGACAGTAAGAGAATTATGCAGTGCTGCCATAACCATGA
GTGATAACACTGCGGCCAACTTACTTCTGACAACGATCGGAGGACCGAAGGAGCTAACCGCTTTTTTGCACAACATGGGGGATCATGTA
ACTCGCCTTGATCGTTGGGAACCGGAGCTGAATGAAGCCATACCAAACGACGAGCGTGACACCACGATGCCTGTAGCAATGGCAACAAC
GTTGCGCAAACTATTAACTGGCGAACTACTTACTCTAGCTTCCCGGCAACAATTAATAGACTGGATGGAGGCGGATAAAGTTGCAGGAC
CACTTCTGCGCTCGGCCCTTCCGGCTGGCTGGTTTATTGCTGATAAATCTGGAGCCGGTGAGCGTGGGTCTCGCGGTATCATTGCAGCA
CTGGGGCCAGATGGTAAGCCCTCCCGTATCGTAGTTATCTACACGACGGGGAGTCAGGCAACTATGGATGAACGAAATAGACAGATCGC
TGAGATAGGTGCCTCACTGATTAAGCATTGGTAACTGTCAGACCAAGTTTACTCATATATACTTTAGATTGATTTAAAACTTCATTTTT
AATTTAAAAGGATCTAGGTGAAGATCCTTTTTGATAATCTCATGACCAAAATCCCTTAACGTGAGTTTTCGTTCCACTGAGCGTCAGAC
CCCGTAGAAAAGATCAAAGGATCTTCTTGAGATCCTTTTTTTCTGCGCGTAATCTGCTGCTTGCAAACAAAAAAACCACCGCTACCAGC
GGTGGTTTGTTTGCCGGATCAAGAGCTACCAACTCTTTTTCCGAAGGTAACTGGCTTCAGCAGAGCGCAGATACCAAATACTGTTCTTC
TAGTGTAGCCGTAGTTAGGCCACCACTTCAAGAACTCTGTAGCACCGCCTACATACCTCGCTCTGCTAATCCTGTTACCAGTGGCTGCT
GCCAGTGGCGATAAGTCGTGTCTTACCGGGTTGGACTCAAGACGATAGTTACCGGATAAGGCGCAGCGGTCGGGCTGAACGGGGGGTTC
GTGCACACAGCCCAGCTTGGAGCGAACGACCTACACCGAACTGAGATACCTACAGCGTGAGCTATGAGAAAGCGCCACGCTTCCCGAAG
GGAGAAAGGCGGACAGGTATCCGGTAAGCGGCAGGGTCGGAACAGGAGAGCGCACGAGGGAGCTTCCAGGGGGAAACGCCTGGTATCTT
TATAGTCCTGTCGGGTTTCGCCACCTCTGACTTGAGCGTCGATTTTTGTGATGCTCGTCAGGGGGGCGGAGCCTATGGAAAAACGCCAG
CAACGCGGCCTTTTTACGGTTCCTGGCCTTTTGCTGGCCTTTTGCTCACATGTTCTTTCCTGCGTTATCCCCTGATTCTGTGGATAACC
GTATTACCGCCTTTGAGTGAGCTGATACCGCTCGCCGCAGCCGAACGACCGAGCGCAGCGAGTCAGTGAGCGAGGAGAAGA

pKRG3-mU6-PUb-hSpCas9

aae U6
<u>*BbsI* sites</u> for sgRNA cloning
sgRNA tracrRNA and <u>U6 terminator</u>
aae PUb promoter
NLS
hSpCas9

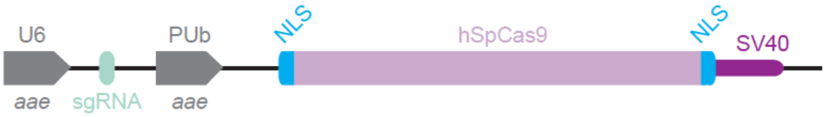

GCGCCCAATACGCAAACCGCCTCTCCCCGCGGGTTGGCCGATTCATTAATGCAGGCAACTCGTGAAAGGTAGGCGGATCAGCGAATGAA
ATCGCCCATCGAGTTGATACGTCCATCCATCGCTAGAACCGCGTTCGCTGTAGAAGACTATATAAGAGCAGAGGCAAGAGTAGTGAAAT
gGAGACGaCGTCTCtGTTTTAGAGCTAGAAATAGCAAGTTAAAATAAGGCTAGTCCGTTATCAACTTGAAAAAGTGGCACCGAGTCGGT
GCTTTTTTGTTTTAGAGCTAGAAATAGCAAGTTAAAATAAGGCTAGTCCGTTTTTAGCGCTTGCGCCAATTCTGCAGACAAATGGCTAT
CTTTACATGTAGCTTGTGCATTGAATCCAATTATAATTTGCCTTGGCACCAGCTGAGCCAGACAAGAAAGAAAGCTTCCCAGAAGTATA
TCGATTTAGAAGGGTTGACGTCACTTGCTGACTGCACTAATACAGCAAATGATGCAATTAGAATGATTCAAGTGAAATTCCCAAATTAC
TGATTTTTCTCTGGATTTGGTTATCAGATTACATTCGAAGCTAAGATTAGCTACCGAAATTGTCGATCAAATCAGGAAATCCTTTCTCT
ATCGAAAAAGGCATTCGCACATCTTCCTCTCTATGCCATATACACGAAGGGTAGGTACATTGACGTCTTTGCCAGAAGTTGAACTGCAT
CGTTCAAGGTACAGAATGAACGACTAACAGACACAAGCACGTTTTGCTGTCCATTCAGACACAGGGATGGTACCCATATTCGATCGATA
TAGAGCCATCCAACCGAACAGAGGTATATGTATGAATGTATTGCTGAAATTTTCTAGAAGTACAACCACCACTACGACAGTGTCTATAA
AACGCCCCTGCAAAGGCGAAACCAGCTCAATCGAATACGTTTCCTAGTGGAGTGAACATTACGCGGCCCAAGTAAGCAGTGCCAGTGCA
AGTGAAGTGAAGTCTCTAGTGAAAAAGAGTGATCCAATTAGCCAGAGGAGAAAATTTCAGAGTGAACAAAGCTTTATTCAAAGGACAAT
TACTATTAAATTGGTGAAAGTGCATTTCGGTGAAGGGAATCTTCTAGTGAAGGTAGGTAAATTAATTGATGAAATTATAGCTATGAGCG
AAAACTAGTTTGGTGAATGATTCCTTTGTCTTTGAATGAGCAAACTATTTTCCAAGATGGCGACTATTGAGCTTTGAGTGATTAGTGAA
AATTTGCAACGCAGTTTCATCATCATTGATAAAACCCAATTGTGATTCACAGCGATAATCATATTTCGTTGAATCATCGCTACTAATTG
AATTAAATTTCTAGAATAATAAGAATAACGTATTTGCTCCGTCACATATCTAAAATAAATATTTTGATGGTTAATTACCCATTAAGGTA
ATATTAACACATATCGAGAAAAACCTTGAGGAAATCGTGAAAACTTGAAGATACGCAATTTCAAAACTACGTAGTTCAAAGTCGAAAAC
AAGTTAATTTTTCACTTAAAAGTAGGGCGTTGTTGTGACGTCATCACCTTCAAGTGTATATTTTTCACTTGGCCTGCGACTGCAAACGC
AGACAAAGCAAAACAAGTTTAAAACCTGTCGTGTCGTGCTCGAAGCCAAAGGCAATGAATCAATATCAAATGAGAGTTTGCATTTCACA
ACCAATTACTCAAGCGTTTCCTCGTTTCTTTTTCTGCTCAACAGAGATTTCAACACCAAGCTCGATAAGCTTGTTCGAATCTCGAGTGC
GCGCTTCCGGAGGTATACACCTAGGCGGTACCACTGCAGTGAATTCGGAGCTCTACCGGTGCCACCATGGCCCCAAAGAAGAAGCGGAA
GGTCGGTATCCACGGAGTCCCAGCAGCCGACAAGAAGTACAGCATCGGCCTGGACATCGGCACCAACTCTGTGGGCTGGGCCGTGATCA
CCGACGAGTACAAGGTGCCCAGCAAGAAATTCAAGGTGCTGGGCAACACCGACCGGCACAGCATCAAGAAGAACCTGATCGGAGCCCTG
CTGTTCGACAGCGGCGAAACAGCCGAGGCCACCCGGCTGAAGAGAACCGCCAGAAGAAGATACACCAGACGGAAGAACCGGATCTGCTA
TCTGCAAGAGATCTTCAGCAACGAGATGGCCAAGGTGGACGACAGCTTCTTCCACAGACTGGAAGAGTCCTTCCTGGTGGAAGAGGATA
AGAAGCACGAGCGGCACCCCATCTTCGGCAACATCGTGGACGAGGTGGCCTACCACGAGAAGTACCCCACCATCTACCACCTGAGAAAG
AAACTGGTGGACAGCACCGACAAGGCCGACCTGCGGCTGATCTATCTGGCCCTGGCCCACATGATCAAGTTCCGGGGCCACTTCCTGAT
CGAGGGCGACCTGAACCCCGACAACAGCGACGTGGACAAGCTGTTCATCCAGCTGGTGCAGACCTACAACCAGCTGTTCGAGGAAAACC
CCATCAACGCCAGCGGCGTGGACGCCAAGGCCATCCTGTCTGCCAGACTGAGCAAGAGCAGACGGCTGGAAAATCTGATCGCCCAGCTG
CCCGGCGAGAAGAAGAATGGCCTGTTCGGAAACCTGATTGCCCTGAGCCTGGGCCTGACCCCCAACTTCAAGAGCAACTTCGACCTGGC
CGAGGATGCCAAACTGCAGCTGAGCAAGGACACCTACGACGACGACCTGGACAACCTGCTGGCCCAGATCGGCGACCAGTACGCCGACC
TGTTTCTGGCCGCCAAGAACCTGTCCGACGCCATCCTGCTGAGCGACATCCTGAGAGTGAACACCGAGATCACCAAGGCCCCCCTGAGC
GCCTCTATGATCAAGAGATACGACGAGCACCACCAGGACCTGACCCTGCTGAAAGCTCTCGTGCGGCAGCAGCTGCCTGAGAAGTACAA
AGAGATTTTCTTCGACCAGAGCAAGAACGGCTACGCCGGCTACATTGACGGCGGAGCCAGCCAGGAAGAGTTCTACAAGTTCATCAAGC
CCATCCTGGAAAAGATGGACGGCACCGAGGAACTGCTCGTGAAGCTGAACAGAGAGGACCTGCTGCGGAAGCAGCGGACCTTCGACAAC
GGCAGCATCCCCCACCAGATCCACCTGGGAGAGCTGCACGCCATTCTGCGGCGGCAGGAAGATTTTTACCCATTCCTGAAGGACAACCG
GGAAAAGATCGAGAAGATCCTGACCTTCCGCATCCCCTACTACGTGGGCCCTCTGGCCAGGGGAAACAGCAGATTCGCCTGGATGACCA
GAAAGAGCGAGGAAACCATCACCCCCTGGAACTTCGAGGAAGTGGTGGACAAGGGCGCTTCCGCCCAGAGCTTCATCGAGCGGATGACC
AACTTCGATAAGAACCTGCCCAACGAGAAGGTGCTGCCCAAGCACAGCCTGCTGTACGAGTACTTCACCGTGTATAACGAGCTGACCAA
AGTGAAATACGTGACCGAGGGAATGAGAAAGCCCGCCTTCCTGAGCGGCGAGCAGAAAAAGGCCATCGTGGACCTGCTGTTCAAGACCA
ACCGGAAAGTGACCGTGAAGCAGCTGAAAGAGGACTACTTCAAGAAAATCGAGTGCTTCGACTCCGTGGAAATCTCCGGCGTGGAAGAT
CGGTTCAACGCCTCCCTGGGCACATACCACGATCTGCTGAAAATTATCAAGGACAAGGACTTCCTGGACAATGAGGAAAACGAGGACAT
TCTGGAAGATATCGTGCTGACCCTGACACTGTTTGAGGACAGAGAGATGATCGAGGAACGGCTGAAAACCTATGCCCACCTGTTCGACG
ACAAAGTGATGAAGCAGCTGAAGCGGCGGAGATACACCGGCTGGGGCAGGCTGAGCCGGAAGCTGATCAACGGCATCCGGGACAAGCAG
TCCGGCAAGACAATCCTGGATTTCCTGAAGTCCGACGGCTTCGCCAACAGAAACTTCATGCAGCTGATCCACGACGACAGCCTGACCTT
TAAAGAGGACATCCAGAAAGCCCAGGTGTCCGGCCAGGGCGATAGCCTGCACGAGCACATTGCCAATCTGGCCGGCAGCCCCGCCATTA
AGAAGGGCATCCTGCAGACAGTGAAGGTGGTGGACGAGCTCGTGAAAGTGATGGGCCGGCACAAGCCCGAGAACATCGTGATCGAAATG
GCCAGAGAGAACCAGACCACCCAGAAGGGACAGAAGAACAGCCGCGAGAGAATGAAGCGGATCGAAGAGGGCATCAAAGAGCTGGGCAG
CCAGATCCTGAAAGAACACCCCGTGGAAAACACCCAGCTGCAGAACGAGAAGCTGTACCTGTACTACCTGCAGAATGGCCGGGATATGT
ACGTGGACCAGGAACTGGACATCAACCGGCTGTCCGACTACGATGTGGACCATATCGTGCCTCAGAGCTTTCTGAAGGACGACTCCATC
GACAACAAGGTGCTGACCAGAAGCGACAAGAACCGGGGCAAGAGCGACAACGTGCCCTCCGAAGAGGTCGTGAAGAAGATGAAGAACTA
CTGGCGGCAGCTGCTGAACGCCAAGCTGATTACCCAGAGAAAGTTCGACAATCTGACCAAGGCCGAGAGAGGCGGCCTGAGCGAACTGG
ATAAGGCCGGCTTCATCAAGAGACAGCTGGTGGAAACCCGGCAGATCACAAAGCACGTGGCACAGATCCTGGACTCCCGGATGAACACT
AAGTACGACGAGAATGACAAGCTGATCCGGGAAGTGAAAGTGATCACCCTGAAGTCCAAGCTGGTGTCCGATTTCCGGAAGGATTTCCA
GTTTTACAAAGTGCGCGAGATCAACAACTACCACCACGCCCACGACGCCTACCTGAACGCCGTCGTGGGAACCGCCCTGATCAAAAAGT
ACCCTAAGCTGGAAAGCGAGTTCGTGTACGGCGACTACAAGGTGTACGACGTGCGGAAGATGATCGCCAAGAGCGAGCAGGAAATCGGC

AAGGCTACCGCCAAGTACTTCTTCTACAGCAACATCATGAACTTTTTCAAGACCGAGATTACCCTGGCCAACGGCGAGATCCGGAAGCG
GCCTCTGATCGAGACAAACGGCGAAACCGGGGAGATCGTGTGGGATAAGGGCCGGGATTTTGCCACCGTGCGGAAAGTGCTGAGCATGC
CCCAAGTGAATATCGTGAAAAAGACCGAGGTGCAGACAGGCGGCTTCAGCAAAGAGTCTATCCTGCCCAAGAGGAACAGCGATAAGCTG
ATCGCCAGAAAGAAGGACTGGGACCCTAAGAAGTACGGCGGCTTCGACAGCCCCACCGTGGCCTATTCTGTGCTGGTGGTGGCCAAAGT
GGAAAAGGGCAAGTCCAAGAAACTGAAGAGTGTGAAAGAGCTGCTGGGGATCACCATCATGGAAAGAAGCAGCTTCGAGAAGAATCCCA
TCGACTTTCTGGAAGCCAAGGGCTACAAAGAAGTGAAAAAGGACCTGATCATCAAGCTGCCTAAGTACTCCCTGTTCGAGCTGGAAAAC
GGCCGGAAGAGAATGCTGGCCTCTGCCGGCGAACTGCAGAAGGGAAACGAACTGGCCCTGCCCTCCAAATATGTGAACTTCCTGTACCT
GGCCAGCCACTATGAGAAGCTGAAGGGCTCCCCCGAGGATAATGAGCAGAAACAGCTGTTTGTGGAACAGCACAAGCACTACCTGGACG
AGATCATCGAGCAGATCAGCGAGTTCTCCAAGAGAGTGATCCTGGCCGACGCTAATCTGGACAAAGTGCTGTCCGCCTACAACAAGCAC
CGGGATAAGCCCATCAGAGAGCAGGCCaAGAATATCATCCACCTGTTTACCCTGACCAATCTGGGAGCCCCTGCCGCCTTCAAGTACTT
TGACACCACCATCGACCGGAAGAGGTACACCAGCACCAAAGAGGTGCTGGACGCCACCCTGATCCACCAGAGCATCACCGGCCTGTACG
AGACACGGATCGACCTGTCTCAGCTGGGAGGCGACAGCCCCAAGAAGAAGAGAAAGGTGGAGGCCAGCTAATAGGACCCAGCTTTCTTG
TACAAAGTGGTGACGTAAGCTAGCAGGATCTTTGTGAAGGAACCTTACTTCTGTGGTGTGACATAATTGGACAAACTACCTACAGAGAT
TTAAAGCTCTAAGGTAAATATAAAATTTTTAAGTGTATAATGTGTTAAACTACTGATTCTAATTGTTTGTGTATTTTAGATTCCAACCT
ATGGAACTGATGAATGGGAGCAGTGGTGGAATGCCTTTAATGAGGAAAACCTGTTTTGCTCAGAAGAAATGCCATCTAGTGATGATGAG
GCTACTGCTGACTCTCAACATTCTACTCCTCCAAAAAAGAAGAGAAAGGTAGttGACCCCAAGGACTTTCCTTCAGAATTGCTAAGTTT
TTTGAGTCATGCTGTGTTTAGTAATAGAACTCTTGCTTGCTTTGCTATTTACACCACAAAGGAAAAAGCTGCACTGCTATACAAGAAAA
TTATGGAAAAATATTCTGTAACCTTTATAAGTAGGCATAACAGTTATAATCATAACATACTGTTTTTTCTTACTCCACACAGGCATAGA
GTGTCTGCTATTAATAACTATGCTCAAAAATTGTGTACCTTTAGCTTTTTAATTTGTAAAGGGGTTAATAAGGAATATTTGATGTATAG
TGCCTTGACTAGAGATCATAATCAGCCATACCACATTTGTAGAGGTTTTACTTGCTTTAAAAAACCTCCCACACCTCCCCCTGAACCTG
AAACATAAAATGAATGCAATTGTTGTTGTTAACTTGTTTATTGCAGCTTATAATGGTTACAAATAAAGCAATAGCATCACAAATTTCAC
AAATAAAGCATTTTTTTCACTGCATTCTAGTTGTGGTTTGTCCAAACTCATCAATGTATCTTATCATGTCTGGATCCCGTTTAAACTAC
GCGTAATTCAAACAGGGTTCTGGCGTCGTTCTCGTACTGTTTTCCCCAGGCCAGTGCTTTAGCGTTATTGAAAAAGGAAGAGTATGAGT
ATTCAACATTTCCGTGTCGCCCTTATTCCCTTTTTTGCGGCATTTTGCCTTCCTGTTTTTGCTCACCCAGAAACGCTGGTGAAAGTAAA
AGATGCTGAAGATCAGTTGGGTGCACGAGTGGGTTACATCGAACTGGATCTCAACAGCGGTAAGATCCTTGAGAGTTTTCGCCCCGAAG
AACGTTTTCCAATGATGAGCACTTTTAAAGTTCTGCTATGTGGCGCGGTATTATCCCGTATTGACGCCGGGCAAGAGCAACTCGGTCGC
CGCATACACTATTCTCAGAATGACTTGGTTGAGTACTCACCAGTCACAGAAAAGCATCTTACGGATGGCATGACAGTAAGAGAATTATG
CAGTGCTGCCATAACCATGAGTGATAACACTGCGGCCAACTTACTTCTGACAACGATCGGAGGACCGAAGGAGCTAACCGCTTTTTTGC
ACAACATGGGGGATCATGTAACTCGCCTTGATCGTTGGGAACCGGAGCTGAATGAAGCCATACCAAACGACGAGCGTGACACCACGATG
CCTGTAGCAATGGCAACAACGTTGCGCAAACTATTAACTGGCGAACTACTTACTCTAGCTTCCCGGCAACAATTAATAGACTGGATGGA
GGCGGATAAAGTTGCAGGACCACTTCTGCGCTCGGCCCTTCCGGCTGGCTGGTTTATTGCTGATAAATCTGGAGCCGGTGAGCGTGGGT
CTCGCGGTATCATTGCAGCACTGGGGCCAGATGGTAAGCCCTCCCGTATCGTAGTTATCTACACGACGGGGAGTCAGGCAACTATGGAT
GAACGAAATAGACAGATCGCTGAGATAGGTGCCTCACTGATTAAGCATTGGTAACTGTCAGACCAAGTTTACTCATATATACTTTAGAT
TGATTTAAAACTTCATTTTTAATTTAAAAGGATCTAGGTGAAGATCCTTTTTGATAATCTCATGACCAAAATCCCTTAACGTGAGTTTT
CGTTCCACTGAGCGTCAGACCCCGTAGAAAAGATCAAAGGATCTTCTTGAGATCCTTTTTTTCTGCGCGTAATCTGCTGCTTGCAAACA
AAAAAACCACCGCTACCAGCGGTGGTTTGTTTGCCGGATCAAGAGCTACCAACTCTTTTTCCGAAGGTAACTGGCTTCAGCAGAGCGCA
GATACCAAATACTGTTCTTCTAGTGTAGCCGTAGTTAGGCCACCACTTCAAGAACTCTGTAGCACCGCCTACATACCTCGCTCTGCTAA
TCCTGTTACCAGTGGCTGCTGCCAGTGGCGATAAGTCGTGTCTTACCGGGTTGGACTCAAGACGATAGTTACCGGATAAGGCGCAGCGG
TCGGGCTGAACGGGGGGTTCGTGCACACAGCCCAGCTTGGAGCGAACGACCTACACCGAACTGAGATACCTACAGCGTGAGCTATGAGA
AAGCGCCACGCTTCCCGAAGGGAGAAAGGCGGACAGGTATCCGGTAAGCGGCAGGGTCGGAACAGGAGAGCGCACGAGGGAGCTTCCAG
GGGGAAACGCCTGGTATCTTTATAGTCCTGTCGGGTTTCGCCACCTCTGACTTGAGCGTCGATTTTTGTGATGCTCGTCAGGGGGGCGG
AGCCTATGGAAAAACGCCAGCAACGCGGCCTTTTTACGGTTCCTGGCCTTTTGCTGGCCTTTTGCTCACATGTTCTTTCCTGCGTTACC
CCTGATTCTGTGGATAACCGTATTACCGCCTTTGAGTGAGCTGATACCGCTCGCCGCAGCCGAACGACCGAGCGCAGCGAGTCAGTGAG
CGAGGAGAAGA

pKRG3-mU6-PUb-hSpCas9-pAc

aae U6
BbsI sites for sgRNA cloning
sgRNA tracrRNA and U6 terminator
aae PUb promoter
NLS
hSpCas9
T2A
pAc

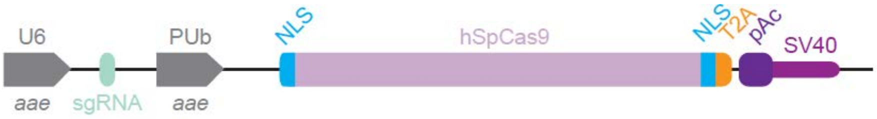

GCGCCCAATACGCAAACCGCCTCTCCCCGCGGGTTGGCCGATTCATTAATGCAGGCAACTCGTGAAAGGTAGGCGGATCAGCGAATGAA
ATCGCCCATCGAGTTGATACGTCCATCCATCGCTAGAACCGCGTTCGCTGTAGAAGACTATATAAGAGCAGAGGCAAGAGTAGTGAAAT
gGAGACGaCGTCTCtGTTTTAGAGCTAGAAATAGCAAGTTAAAATAAGGCTAGTCCGTTATCAACTTGAAAAAGTGGCACCGAGTCGGT
GCTTTTTTGTTTTAGAGCTAGAAATAGCAAGTTAAAATAAGGCTAGTCCGTTTTTAGCGCTTGCGCCAATTCTGCAGACAAATGGCTAT
CTTTACATGTAGCTTGTGCATTGAATCCAATTATAATTTGCCTTGGCACCAGCTGAGCCAGACAAGAAAGAAAGCTTCCCAGAAGTATA
TCGATTTAGAAGGGTTGACGTCACTTGCTGACTGCACTAATACAGCAAATGATGCAATTAGAATGATTCAAGTGAAATTCCCAAATTAC
TGATTTTTCTCTGGATTTGGTTATCAGATTACATTCGAAGCTAAGATTAGCTACCGAAATTGTCGATCAAATCAGGAAATCCTTTCTCT
ATCGAAAAAGGCATTCGCACATCTTCCTCTCTATGCCATATACACGAAGGGTAGGTACATTGACGTCTTTGCCAGAAGTTGAACTGCAT
CGTTCAAGGTACAGAATGAACGACTAACAGACACAAGCACGTTTTGCTGTCCATTCAGACACAGGGATGGTACCCATATTCGATCGATA
TAGAGCCATCCAACCGAACAGAGGTATATGTATGAATGTATTGCTGAAATTTTCTAGAAGTACAACCACCACTACGACAGTGTCTATAA
AACGCCCCTGCAAAGGCGAAACCAGCTCAATCGAATACGTTTCCTAGTGGAGTGAACATTACGCGGCCCAAGTAAGCAGTGCCAGTGCA
AGTGAAGTGAAGTCTCTAGTGAAAAAGAGTGATCCAATTAGCCAGAGGAGAAAATTTCAGAGTGAACAAAGCTTTATTCAAAGGACAAT
TACTATTAAATTGGTGAAAGTGCATTTCGGTGAAGGGAATCTTCTAGTGAAGGTAGGTAAATTAATTGATGAAATTATAGCTATGAGCG
AAAACTAGTTTGGTGAATGATTCCTTTGTCTTTGAATGAGCAAACTATTTTCCAAGATGGCGACTATTGAGCTTTGAGTGATTAGTGAA
AATTTGCAACGCAGTTTCATCATCATTGATAAAACCCAATTGTGATTCACAGCGATAATCATATTTCGTTGAATCATCGCTACTAATTG
AATTAAATTTCTAGAATAATAAGAATAACGTATTTGCTCCGTCACATATCTAAAATAAATATTTTGATGGTTAATTACCCATTAAGGTA
ATATTAACACATATCGAGAAAAACCTTGAGGAAATCGTGAAAACTTGAAGATACGCAATTTCAAAACTACGTAGTTCAAAGTCGAAAAC
AAGTTAATTTTTCACTTAAAAGTAGGGCGTTGTTGTGACGTCATCACCTTCAAGTGTATATTTTTCACTTGGCCTGCGACTGCAAACGC
AGACAAAGCAAAACAAGTTTAAAACCTGTCGTGTCGTGCTCGAAGCCAAAGGCAATGAATCAATATCAAATGAGAGTTTGCATTTCACA
ACCAATTACTCAAGCGTTTCCTCGTTTCTTTTTCTGCTCAACAGAGATTTCAACACCAAGCTCGATAAGCTTGTTCGAATCTCGAGTGC
GCGCTTCCGGAGGTATACACCTAGGCGGTACCACTGCAGTGAATTCGGAGCTCTACCGGTGCCACCATGGCCCCAAAGAAGAAGCGGAA
GGTCGGTATCCACGGAGTCCCAGCAGCCGACAAGAAGTACAGCATCGGCCTGGACATCGGCACCAACTCTGTGGGCTGGGCCGTGATCA
CCGACGAGTACAAGGTGCCCAGCAAGAAATTCAAGGTGCTGGGCAACACCGACCGGCACAGCATCAAGAAGAACCTGATCGGAGCCCTG
CTGTTCGACAGCGGCGAAACAGCCGAGGCCACCCGGCTGAAGAGAACCGCCAGAAGAAGATACACCAGACGGAAGAACCGGATCTGCTA
TCTGCAAGAGATCTTCAGCAACGAGATGGCCAAGGTGGACGACAGCTTCTTCCACAGACTGGAAGAGTCCTTCCTGGTGGAAGAGGATA
AGAAGCACGAGCGGCACCCCATCTTCGGCAACATCGTGGACGAGGTGGCCTACCACGAGAAGTACCCCACCATCTACCACCTGAGAAAG
AAACTGGTGGACAGCACCGACAAGGCCGACCTGCGGCTGATCTATCTGGCCCTGGCCCACATGATCAAGTTCCGGGGCCACTTCCTGAT
CGAGGGCGACCTGAACCCCGACAACAGCGACGTGGACAAGCTGTTCATCCAGCTGGTGCAGACCTACAACCAGCTGTTCGAGGAAAACC
CCATCAACGCCAGCGGCGTGGACGCCAAGGCCATCCTGTCTGCCAGACTGAGCAAGAGCAGACGGCTGGAAAATCTGATCGCCCAGCTG
CCCGGCGAGAAGAAGAATGGCCTGTTCGGAAACCTGATTGCCCTGAGCCTGGGCCTGACCCCCAACTTCAAGAGCAACTTCGACCTGGC
CGAGGATGCCAAACTGCAGCTGAGCAAGGACACCTACGACGACGACCTGGACAACCTGCTGGCCCAGATCGGCGACCAGTACGCCGACC
TGTTTCTGGCCGCCAAGAACCTGTCCGACGCCATCCTGCTGAGCGACATCCTGAGAGTGAACACCGAGATCACCAAGGCCCCCCTGAGC
GCCTCTATGATCAAGAGATACGACGAGCACCACCAGGACCTGACCCTGCTGAAAGCTCTCGTGCGGCAGCAGCTGCCTGAGAAGTACAA
AGAGATTTTCTTCGACCAGAGCAAGAACGGCTACGCCGGCTACATTGACGGCGGAGCCAGCCAGGAAGAGTTCTACAAGTTCATCAAGC
CCATCCTGGAAAAGATGGACGGCACCGAGGAACTGCTCGTGAAGCTGAACAGAGAGGACCTGCTGCGGAAGCAGCGGACCTTCGACAAC
GGCAGCATCCCCCACCAGATCCACCTGGGAGAGCTGCACGCCATTCTGCGGCGGCAGGAAGATTTTTACCCATTCCTGAAGGACAACCG
GGAAAAGATCGAGAAGATCCTGACCTTCCGCATCCCCTACTACGTGGGCCCTCTGGCCAGGGGAAACAGCAGATTCGCCTGGATGACCA
GAAAGAGCGAGGAAACCATCACCCCCTGGAACTTCGAGGAAGTGGTGGACAAGGGCGCTTCCGCCCAGAGCTTCATCGAGCGGATGACC
AACTTCGATAAGAACCTGCCCAACGAGAAGGTGCTGCCCAAGCACAGCCTGCTGTACGAGTACTTCACCGTGTATAACGAGCTGACCAA
AGTGAAATACGTGACCGAGGGAATGAGAAAGCCCGCCTTCCTGAGCGGCGAGCAGAAAAAGGCCATCGTGGACCTGCTGTTCAAGACCA
ACCGGAAAGTGACCGTGAAGCAGCTGAAAGAGGACTACTTCAAGAAAATCGAGTGCTTCGACTCCGTGGAAATCTCCGGCGTGGAAGAT
CGGTTCAACGCCTCCCTGGGCACATACCACGATCTGCTGAAAATTATCAAGGACAAGGACTTCCTGGACAATGAGGAAAACGAGGACAT
TCTGGAAGATATCGTGCTGACCCTGACACTGTTTGAGGACAGAGAGATGATCGAGGAACGGCTGAAAACCTATGCCCACCTGTTCGACG
ACAAAGTGATGAAGCAGCTGAAGCGGCGGAGATACACCGGCTGGGGCAGGCTGAGCCGGAAGCTGATCAACGGCATCCGGGACAAGCAG
TCCGGCAAGACAATCCTGGATTTCCTGAAGTCCGACGGCTTCGCCAACAGAAACTTCATGCAGCTGATCCACGACGACAGCCTGACCTT
TAAAGAGGACATCCAGAAAGCCCAGGTGTCCGGCCAGGGCGATAGCCTGCACGAGCACATTGCCAATCTGGCCGGCAGCCCCGCCATTA
AGAAGGGCATCCTGCAGACAGTGAAGGTGGTGGACGAGCTCGTGAAAGTGATGGGCCGGCACAAGCCCGAGAACATCGTGATCGAAATG
GCCAGAGAGAACCAGACCACCCAGAAGGGACAGAAGAACAGCCGCGAGAGAATGAAGCGGATCGAAGAGGGCATCAAAGAGCTGGGCAG
CCAGATCCTGAAAGAACACCCCGTGGAAAACACCCAGCTGCAGAACGAGAAGCTGTACCTGTACTACCTGCAGAATGGGCGGGATATGT
ACGTGGACCAGGAACTGGACATCAACCGGCTGTCCGACTACGATGTGGACCATATCGTGCCTCAGAGCTTTCTGAAGGACGACTCCATC
GACAACAAGGTGCTGACCAGAAGCGACAAGAACCGGGGCAAGAGCGACAACGTGCCCTCCGAAGAGGTCGTGAAGAAGATGAAGAACTA
CTGGCGGCAGCTGCTGAACGCCAAGCTGATTACCCAGAGAAAGTTCGACAATCTGACCAAGGCCGAGAGAGGCGGCCTGAGCGAACTGG
ATAAGGCCGGCTTCATCAAGAGACAGCTGGTGGAAACCCGGCAGATCACAAAGCACGTGGCACAGATCCTGGACTCCCGGATGAACACT
AAGTACGACGAGAATGACAAGCTGATCCGGGAAGTGAAAGTGATCACCCTGAAGTCCAAGCTGGTGTCCGATTTCCGGAAGGATTTCCA

GTTTTACAAAGTGCGCGAGATCAACAACTACCACCACGCCCACGACGCCTACCTGAACGCCGTCGTGGGAACCGCCCTGATCAAAAAGT
ACCCTAAGCTGGAAAGCGAGTTCGTGTACGGCGACTACAAGGTGTACGACGTGCGGAAGATGATCGCCAAGAGCGAGCAGGAAATCGGC
AAGGCTACCGCCAAGTACTTCTTCTACAGCAACATCATGAACTTTTTCAAGACCGAGATTACCCTGGCCAACGGCGAGATCCGGAAGCG
GCCTCTGATCGAGACAAACGGCGAAACCGGGGAGATCGTGTGGGATAAGGGCCGGGATTTTGCCACCGTGCGGAAAGTGCTGAGCATGC
CCCAAGTGAATATCGTGAAAAAGACCGAGGTGCAGACAGGCGGCTTCAGCAAAGAGTCTATCCTGCCCAAGAGGAACAGCGATAAGCTG
ATCGCCAGAAAGAAGGACTGGGACCCTAAGAAGTACGGCGGCTTCGACAGCCCCACCGTGGCCTATTCTGTGCTGGTGGTGGCCAAAGT
GGAAAAGGGCAAGTCCAAGAAACTGAAGAGTGTGAAAGAGCTGCTGGGGATCACCATCATGGAAAGAAGCAGCTTCGAGAAGAATCCCA
TCGACTTTCTGGAAGCCAAGGGCTACAAAGAAGTGAAAAAGGACCTGATCATCAAGCTGCCTAAGTACTCCCTGTTCGAGCTGGAAAAC
GGCCGGAAGAGAATGCTGGCCTCTGCCGGCGAACTGCAGAAGGGAAACGAACTGGCCCTGCCCTCCAAATATGTGAACTTCCTGTACCT
GGCCAGCCACTATGAGAAGCTGAAGGGCTCCCCCGAGGATAATGAGCAGAAACAGCTGTTTGTGGAACAGCACAAGCACTACCTGGACG
AGATCATCGAGCAGATCAGCGAGTTCTCCAAGAGAGTGATCCTGGCCGACGCTAATCTGGACAAAGTGCTGTCCGCCTACAACAAGCAC
CGGGATAAGCCCATCAGAGAGCAGGCCaAGAATATCATCCACCTGTTTACCCTGACCAATCTGGGAGCCCCTGCCGCCTTCAAGTACTT
TGACACCACCATCGACCGGAAGAGGTACACCAGCACCAAAGAGGTGCTGGACGCCACCCTGATCCACCAGAGCATCACCGGCCTGTACG
AGACACGGATCGACCTGTCTCAGCTGGGAGGCGACAAAAGGCCGGCGGCCACGAAAAAGGCCGGCCAGGCAAAAAAGAAAAAGGGATCA
GGCAAGCTTGAGGGCAGAGGAAGTCTTCTAACATGCGGTGACGTGGAGGAGAATCCCGGCCCTGCTAGCGGTAGCGGCAGCGGTAGCAT
GACCGAGTACAAGCCCACGGTGCGCCTCGCCACCCGCGACGACGTCCCCCGGGCCGTACGCACCCTCGCCGCCGCGTTCGCCGACTACC
CCGCCACGCGCCACACCGTCGACCCGGACCGCCACATCGAGCGGGTCACCGAGCTGCAAGAACTCTTCCTCACGCGCGTCGGGCTCGAC
ATCGGCAAGGTGTGGGTCGCGGACGACGGCGCCGCGGTGGCGGTCTGGACCACGCCGGAGAGCGTCGAAGCGGGGGCGGTGTTCGCCGA
GATCGGCCCGCGCATGGCCGAGTTGAGCGGTTCCCGGCTGGCCGCGCAGCAACAGATGGAAGGCCTCCTGGCGCCGCACCGGCCCAAGG
AGCCCGCGTGGTTCCTGGCCACCGTCGGCGTCTCGCCCGACCACCAGGGCAAGGGTCTGGGCAGCGCCGTCGTGCTCCCCGGAGTGGAG
GCGGCCGAGCGCGCCGGGGTGCCCGCCTTCCTGGAGACCTCCGCGCCCCGCAACCTCCCCTTCTACGAGCGGCTCGGCTTCACCGTCAC
CGCCGACGTCGAGGTGCCCGAAGGACCGCGCACCTGGTGCATGACCCGCAAGCCCGGTGCCTAATAGGACCCAGCTTTCTTGTACAAAG
TGGTGACGTAAGCTAGCAGGATCTTTGTGAAGGAACCTTACTTCTGTGGTGTGACATAATTGGACAAACTACCTACAGAGATTTAAAGC
TCTAAGGTAAATATAAAATTTTTAAGTGTATAATGTGTTAAACTACTGATTCTAATTGTTTGTGTATTTTAGATTCCAACCTATGGAAC
TGATGAATGGGAGCAGTGGTGGAATGCCTTTAATGAGGAAAACCTGTTTTGCTCAGAAGAAATGCCATCTAGTGATGATGAGGCTACTG
CTGACTCTCAACATTCTACTCCTCCAAAAAAGAAGAGAAAGGTAGttGACCCCAAGGACTTTCCTTCAGAATTGCTAAGTTTTTTGAGT
CATGCTGTGTTTAGTAATAGAACTCTTGCTTGCTTTGCTATTTACACCACAAAGGAAAAAGCTGCACTGCTATACAAGAAAATTATGGA
AAAATATTCTGTAACCTTTATAAGTAGGCATAACAGTTATAATCATAACATACTGTTTTTTCTTACTCCACACAGGCATAGAGTGTCTG
CTATTAATAACTATGCTCAAAAATTGTGTACCTTTAGCTTTTTAATTTGTAAAGGGGTTAATAAGGAATATTTGATGTATAGTGCCTTG
ACTAGAGATCATAATCAGCCATACCACATTTGTAGAGGTTTTACTTGCTTTAAAAAACCTCCCACACCTCCCCCTGAACCTGAAACATA
AAATGAATGCAATTGTTGTTGTTAACTTGTTTATTGCAGCTTATAATGGTTACAAATAAAGCAATAGCATCACAAATTTCACAAATAAA
GCATTTTTTTCACTGCATTCTAGTTGTGGTTTGTCCAAACTCATCAATGTATCTTATCATGTCTGGATCCCGTTTAAACTACGCGTAAT
TCAAACAGGGTTCTGGCGTCGTTCTCGTACTGTTTTCCCCAGGCCAGTGCTTTAGCGTTATTGAAAAAGGAAGAGTATGAGTATTCAAC
ATTTCCGTGTCGCCCTTATTCCCTTTTTTGCGGCATTTTGCCTTCCTGTTTTTGCTCACCCAGAAACGCTGGTGAAAGTAAAAGATGCT
GAAGATCAGTTGGGTGCACGAGTGGGTTACATCGAACTGGATCTCAACAGCGGTAAGATCCTTGAGAGTTTTCGCCCCGAAGAACGTTT
TCCAATGATGAGCACTTTTAAAGTTCTGCTATGTGGCGCGGTATTATCCCGTATTGACGCCGGGCAAGAGCAACTCGGTCGCCGCATAC
ACTATTCTCAGAATGACTTGGTTGAGTACTCACCAGTCACAGAAAAGCATCTTACGGATGGCATGACAGTAAGAGAATTATGCAGTGCT
GCCATAACCATGAGTGATAACACTGCGGCCAACTTACTTCTGACAACGATCGGAGGACCGAAGGAGCTAACCGCTTTTTTGCACAACAT
GGGGGATCATGTAACTCGCCTTGATCGTTGGGAACCGGAGCTGAATGAAGCCATACCAAACGACGAGCGTGACACCACGATGCCTGTAG
CAATGGCAACAACGTTGCGCAAACTATTAACTGGCGAACTACTTACTCTAGCTTCCCGGCAACAATTAATAGACTGGATGGAGGCGGAT
AAAGTTGCAGGACCACTTCTGCGCTCGGCCCTTCCGGCTGGCTGGTTTATTGCTGATAAATCTGGAGCCGGTGAGCGTGGGTCTCGCGG
TATCATTGCAGCACTGGGGCCAGATGGTAAGCCCTCCCGTATCGTAGTTATCTACACGACGGGGAGTCAGGCAACTATGGATGAACGAA
ATAGACAGATCGCTGAGATAGGTGCCTCACTGATTAAGCATTGGTAACTGTCAGACCAAGTTTACTCATATATACTTTAGATTGATTTA
AAACTTCATTTTTAATTTAAAAGGATCTAGGTGAAGATCCTTTTTGATAATCTCATGACCAAAATCCCTTAACGTGAGTTTTCGTTCCA
CTGAGCGTCAGACCCCGTAGAAAAGATCAAAGGATCTTCTTGAGATCCTTTTTTTCTGCGCGTAATCTGCTGCTTGCAAACAAAAAAAC
CACCGCTACCAGCGGTGGTTTGTTTGCCGGATCAAGAGCTACCAACTCTTTTTCCGAAGGTAACTGGCTTCAGCAGAGCGCAGATACCA
AATACTGTTCTTCTAGTGTAGCCGTAGTTAGGCCACCACTTCAAGAACTCTGTAGCACCGCCTACATACCTCGCTCTGCTAATCCTGTT
ACCAGTGGCTGCTGCCAGTGGCGATAAGTCGTGTCTTACCGGGTTGGACTCAAGACGATAGTTACCGGATAAGGCGCAGCGGTCGGGCT
GAACGGGGGGTTCGTGCACACAGCCCAGCTTGGAGCGAACGACCTACACCGAACTGAGATACCTACAGCGTGAGCTATGAGAAAGCGCC
ACGCTTCCCGAAGGGAGAAAGGCGGACAGGTATCCGGTAAGCGGCAGGGTCGGAACAGGAGAGCGCACGAGGGAGCTTCCAGGGGGAAA
CGCCTGGTATCTTTATAGTCCTGTCGGGTTTCGCCACCTCTGACTTGAGCGTCGATTTTTGTGATGCTCGTCAGGGGGGCGGAGCCTAT
GGAAAAACGCCAGCAACGCGGCCTTTTTACGGTTCCTGGCCTTTTGCTGGCCTTTTGCTCACATGTTCTTTCCTGCGTTATCCCCTGAT
TCTGTGGATAACCGTATTACCGCCTTTGAGTGAGCTGATACCGCTCGCCGCAGCCGAACGACCGAGCGCAGCGAGTCAGTGAGCGAGGA
GAAGA

pSL1180-HR-Aag2-loxP-PUb-RFP-loxP-3xFLAG-AGO1

AGO1 homology with silent mutations at sgRNA cleavage sites
loxP sites
aae PUb promoter
RFP
3xFLAG

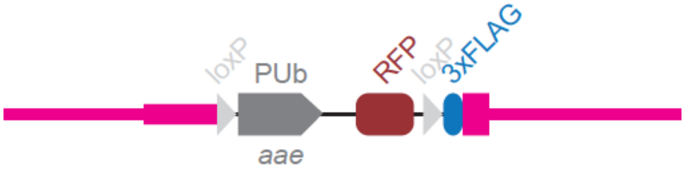

GCGCCCAATACGCAAACCGCCTCTCCCCGCGCGTTGGCCGATTCATTAATGCAGCTGGCACGACAGGTTTCCCGACTGGAAAGCGGGCA
GTGAGCGCAACGCAATTAATGTGAGTTAGCTCACTCATTAGGCACCCCAGGCTTTACACTTTATGCTTCCGGCTCGTATGTTGTGTGGA
ATTGTGAGCGGATAACAATTTCACACAGGAAACAGCTATGACCATGATTACGAATTCGACATGTAGGACATGTGGGGTAGTTCCAGAAA
TCCAGATAAACGATGGACATTTGAATTATCCCCAAACCTCCATCATGTTCTGTTACTTGATATCAACTCAAGGAACCCCGCACTAAAAT
TGAATTTCATGGCTACTATGATAACCGTGTTAAACCGTTTTCTAAAAGTTTCTGTCGCATGACTCGTTACAAATGACGTTACAAATCTA
TAATTTCTATAAATCCCTTCAAAATTGACTCTTGGAAATTTATCTGTATTAAAATTATATAATTATTTTAAAAGCTGGTAAATCACCAA
CATTTTGACATCCATTTCATCCAGAATCGTTCTATTGTTTATGCGGTGAATGCCGAAAAAAATATACCTTCAATGATTCATTGCCGATG
TTTCAACTGAACTCAAACTCAGAAATATGAAATTTCACGCATTTAGATAGATGACTACTCTTTTTTCAGAGTTTTAGTTCACAGCAAAG
ATAAAAGGTAATTCAAATGAGAGCTGCTGTAGAAAGTTTCTCGCTCAATGTATGATCTCGCCTTAAAGCCATTGGTAACCAAGTGTGTA
GCTTGTTGTTACTACGCAAACGAATGGATTTACACGTTACTTAAAAATGCTACAATGAAGATTCTCTATCTCCCAGTGGCGATAGTTAT
ATTTTGGTCCATTCATTTGCTTAAAAACAACGAGCTACACATCAATGGCTTTTAGATCGAGATCACAATTTATGCGAGATTTCCTCAAG
TAATTTATTGCCAAGAAATTTCGTGAATAATAATAATTATAATGATTATTCTGTTTCATGAGCGCATCCATTTTGGAGCGTCCGTAAAA
TGAAACAAAACGGTAAACCGCAACCTGGCTGTTTACTTGCAGCGGTTTTTCGGTACCTAAACTTGCGTACAAGTCGAACTCGTCTAAAA
CATAACGCCTACCCAGGCTGCTGGTAGCGAAACGTTGGGGGGCGATGAAGAAAACTTGTCTCACTCGCCATCATACGAGAGTTTCGCGA
CGAGTTCAGCAGACGTGCGTGTTTAGCGCGACGACGAAAAGTGCAAAAATTCGGCCGTAATTAGTTCGCTTCCGTTTTTCCGAGTGCCT
CCGATCGTTAGCGGACCGTTCCGGGTGTGATTTAAGCGTGAAAATAACTTCGTATAATGTATGCTATACGAAGTTATTATCTTTACATG
TAGCTTGTGCATTGAATCCAATTATAATTTGCCTTGGCACCAGCTGAGCCAGACAAGAAAGAAAGCTTCCCAGAAGTATATCGATTTAG
AAGGGTTGACGTCACTTGCTGACTGCACTAATACAGCAAATGATGCAATTAGAATGATTCAAGTGAAATTCCCAAATTACTGATTTTTC
TCTGGATTTGGTTATCAGATTACATTCGAAGCTAAGATTAGCTACCGAAATTGTCGATCAAATCAGGAAATCCTTTCTCTATCGAAAAA
GGCATTCGCACATCTTCCTCTCTATGCCATATACACGAAGGGTAGGTACATTGACGTCTTTGCCAGAAGTTGAACTGCATCGTTCAAGG
TACAGAATGAACGACTAACAGACACAAGCACGTTTTGCTGTCCATTCAGACACAGGGATGGTACCCATATTCGATCGATATAGAGCCAT
CCAACCGAACAGAGGTATATGTATGAATGTATTGCTGAAATTTTCTAGAAGTACAACCACCACTACGACAGTGTCTATAAAACGCCCCT
GCAAAGGCGAAACCAGCTCAATCGAATACGTTTCCTAGTGGAGTGAACATTACGCGGCCCAAGTAAGCAGTGCCAGTGCAAGTGAAGTG
AAGTCTCTAGTGAAAAAGAGTGATCCAATTAGCCAGAGGAGAAAATTTCAGAGTGAACAAAGCTTTATTCAAAGGACAATTACTATTAA
ATTGGTGAAAGTGCATTTCGGTGAAGGGAATCTTCTAGTGAAGGTAGGTAAATTAATTGATGAAATTATAGCTATGAGCGAAAACTAGT
TTGGTGAATGATTCCTTTGTCTTTGAATGAGCAAACTATTTTCCAAGATGGCGACTATTGAGCTTTGAGTGATTAGTGAAAATTTGCAA
CGCAGTTTCATCATCATTGATAAAACCCAATTGTGATTCACAGCGATAATCATATTTCGTTGAATCATCGCTACTAATTGAATTAAATT
TCTAGAATAATAAGAATAACGTATTTGCTCCGTCACATATCTAAAATAAATATTTTGATGGTTAATTACCCATTAAGGTAATATTAACA
CATATCGAGAAAAACCTTGAGGAAATCGTGAAAACTTGAAGATACGCAATTTCAAAACTACGTAGTTCAAAGTCGAAAACAAGTTAATT
TTTCACTTAAAAGTAGGGCGTTGTTGTGACGTCATCACCTTCAAGTGTATATTTTTCACTTGGCCTGCGACTGCAAACGCAGACAAAGC
AAAACAAGTTTAAAACCTGTCGTGTCGTGCTCGAAGCCAAAGGCAATGAATCAATATCAAATGAGAGTTTGCATTTCACAACCAATTAC
TCAAGCGTTTCCTCGTTTCTTTTTCTGCTCAACAGAGATTTCAACACCAAGCTCGATAAGCTTGTTCGAATCTCGAGTGCGCGCTTCCG
GAGGTATACACCTAGGCGGTACCACTGCAGTGAATTCGGAGCTCTACCGCGGCCGCATGAGCGAGCTGATCAAGGAGAACATGCACATG
AAGCTGTACATGGAGGGCACCGTGAACAACCACCACTTCAAGTGCACATCCGAGGGCGAAGGCAAGCCCTACGAGGGCACCCAGACCAT
GAAGATCAAGGTGGTCGAGGGCGGCCCTCTCCCCTTCGCCTTCGACATCCTGGCTACCAGCTTCATGTACGGCAGCAAAGCCTTCATCA
ACCACACCCAGGGCATCCCCGACTTCTTTAAGCAGTCCTTCCCTGAGGGCTTCACATGGGAGAGAATCACCACATACGAAGACGGGGGC
GTGCTGACCGCTACCCAGGACACCAGCTTCCAGAACGGCTGCATCATCTACAACGTCAAGATCAACGGGGTGAACTTCCCATCCAACGG
CCCTGTGATGCAGAAGAAAACACGCGGCTGGGAGGCCAACACCGAGATGCTGTACCCCGCTGACGGCGGCCTGAGAGGCCACAGCCAGA
TGGCCCTGAAGCTCGTGGGCGGGGGCTACCTGCACTGCTCCTTCAAGACCACATACAGATCCAAGAAACCCGCTAAGAACCTCAAGATG
CCCGGCTTCCACTTCGTGGACCACAGACTGGAAAGAATCAAGGAGGCCGACAAAGAGACCTACGTCGAGCAGCACGAGATGGCTGTGGC
CAAGTACTGCGACCTCCCTAGCAAACTGGGGCACAGATAATTAATTAATAACTTCGTATAATGTATGCTATACGAAGTTATATGGATTA
CAAGGATCACGATGGAGATTACAAGGATCACGATATCGATTACAAGGATGATGATAAGTATCCGGTGGGTCAACGTAAGTACCGTTTCC
GGACGTTTGAAAAAAGTGCAATTCACAAGAGAAGAAAAAAAAAaGTGTTGgCGAGGGGACCTTCCTTCCTCCCTCTCGTCTCTCTCGCT
GTGTAAATATTTCTTCCCCCATGTGTGTAATTTTCCTGGCCTGCTGCTCTGTTTGGTGCTTCTCTGGACGTTTTTGTGCGTGTGcGTTG
GTGTGATGGGGGGCTTTTCCTGTGTACGGACGAAGAACTTTGTGTCGGTGTGCGTGTGAGATGAGGCCCTCGCCAGAATGAAAATAAAC
CTCATCAACAATGAAAAGTGTCACCACTTTATGGAATAAAAAACGTTTTTATGCAGAAGAGCATGTGAATTATGTATCACTTGGCGATG
GAATTGGGAACATTGATGCTTCCGGACCGATGGAATAATGGAAAACCAGATGTTTCTGCACGTTCTTTGGCACCTTGGCAATGTGTACT
AATCAACACCTCCCTCGAAAAATATACTTGGTGATCAGAATGTTTGTGTTTGTGAATAGTAGGTAGTTTCCGTGTGTGGGTGTGTGTCT
TTGCTAATCATCCTGCTCCAGAAAAAAAGGAAAACCACCAATTTTAATCATCATTTTCTGCGGCTTCGGAGACTGATAAGAGGAATACA
ACTGTTTGCAAACAGTGGATTGAAAACAATGGATAGAAGTTTTATTGGTTCACTTCAGTTCTAAACAGCGGATCCATCTGAAAAGTTAT
ATGAAATTGTACCCCCTTTTCAGTGCTGGGAAATTATTGCCTTTTGTGTCGCTCTTTGCTCACAACTACATACGGAGATGCAGACAGAG
CTCGCATTATGTTGGTGGTGCTCTGTCGCTGGCCATCCGAGAATCGGAAACATCGTGCGGATGGAACATGGAAGAAGAGGACGTTGTAG
GGTTTACCATTATGTATATGCGTTCAGGCGACATCAGACGAGGTGGGTCTTCTCGAAATGATGGGTGGTTGACTGAGTAGCACCTGCTG
TTCATGTGCGGAAATGGAATAACGTGGAGATGCAATGTTCTTCAACGCTAGAAGGATTCGGGGAGACGAAAAGCAAATCCGGTGGCGGC
CGCCTGCAGCTGGCGCCATCGATACGCGTACGTCGCGACCGCGGACATGTACAGAGCTCGAGAAGTACTAGTGGCCACGTGGGCCGTGC

ACCTTAAGCTTGGCACTGGCCGTCGTTTTACAACGTCGTGACTGGGAAAACCCTGGCGTTACCCAACTTAATCGCCTTGCAGCACATCC
CCCTTTCGCCAGCTGGCGTAATAGCGAAGAGGCCCGCACCGATCGCCCTTCCCAACAGTTGCGCAGCCTGAATGGCGAATGGCGCCTGA
TGCGGTATTTTCTCCTTACGCATCTGTGCGGTATTTCACACCGCATACGTCAAAGCAACCATAGTACGCGCCCTGTAGCGGCGCATTAA
GCGCGGCGGGTGTGGTGGTTACGCGCAGCGTGACCGCTACACTTGCCAGCGCCCTAGCGCCCGCTCCTTTCGCTTTCTTCCCTTCCTTT
CTCGCCACGTTCGCCGGCTTTCCCCGTCAAGCTCTAAATCGGGGGCTCCCTTTAGGGTTCCGATTTAGTGCTTTACGGCACCTCGACCC
CAAAAAACTTGATTTGGGTGATGGTTCACGTAGTGGGCCATCGCCCTGATAGACGGTTTTTCGCCCTTTGACGTTGGAGTCCACGTTCT
TTAATAGTGGACTCTTGTTCCAAACTGGAACAACACTCAACCCTATCTCGGGCTATTCTTTTGATTTATAAGGGATTTTGCCGATTTCG
GCCTATTGGTTAAAAAATGAGCTGATTTAACAAAAATTTAACGCGAATTTTAACAAAATATTAACGTTTACAATTTTATGGTGCACTCT
CAGTACAATCTGCTCTGATGCCGCATAGTTAAGCCAGCCCCGACACCCGCCAACACCCGCTGACGCGCCCTGACGGGCTTGTCTGCTCC
CGGCATCCGCTTACAGACAAGCTGTGACCGTCTCCGGGAGCTGCATGTGTCAGAGGTTTTCACCGTCATCACCGAAACGCGCGAGACGA
AAGGGCCTCGTGATACGCCTATTTTTATAGGTTAATGTCATGATAATAATGGTTTCTTAGACGTCAGGTGGCACTTTTCGGGGAAATGT
GCGCGGAACCCCTATTTGTTTATTTTTCTAAATACATTCAAATATGTATCCGCTCATGAGACAATAACCCTGATAAATGCTTCAATAAT
ATTGAAAAAGGAAGAGTATGAGTATTCAACATTTCCGTGTCGCCCTTATTCCCTTTTTTGCGGCATTTTGCCTTCCTGTTTTTGCTCAC
CCAGAAACGCTGGTGAAAGTAAAAGATGCTGAAGATCAGTTGGGTGCACGAGTGGGTTACATCGAACTGGATCTCAACAGCGGTAAGAT
CCTTGAGAGTTTTCGCCCCGAAGAACGTTTTCCAATGATGAGCACTTTTAAAGTTCTGCTATGTGGCGCGGTATTATCCCGTATTGACG
CCGGGCAAGAGCAACTCGGTCGCCGCATACACTATTCTCAGAATGACTTGGTTGAGTACTCACCAGTCACAGAAAAGCATCTTACGGAT
GGCATGACAGTAAGAGAATTATGCAGTGCTGCCATAACCATGAGTGATAACACTGCGGCCAACTTACTTCTGACAACGATCGGAGGACC
GAAGGAGCTAACCGCTTTTTTGCACAACATGGGGGATCATGTAACTCGCCTTGATCGTTGGGAACCGGAGCTGAATGAAGCCATACCAA
ACGACGAGCGTGACACCACGATGCCTGTAGCAATGGCAACAACGTTGCGCAAACTATTAACTGGCGAACTACTTACTCTAGCTTCCCGG
CAACAATTAATAGACTGGATGGAGGCGGATAAAGTTGCAGGACCACTTCTGCGCTCGGCCCTTCCGGCTGGCTGGTTTATTGCTGATAA
ATCTGGAGCCGGTGAGCGTGGGTCTCGCGGTATCATTGCAGCACTGGGGCCAGATGGTAAGCCCTCCCGTATCGTAGTTATCTACACGA
CGGGGAGTCAGGCAACTATGGATGAACGAAATAGACAGATCGCTGAGATAGGTGCCTCACTGATTAAGCATTGGTAACTGTCAGACCAA
GTTTACTCATATATACTTTAGATTGATTTAAAACTTCATTTTTAATTTAAAAGGATCTAGGTGAAGATCCTTTTTGATAATCTCATGAC
CAAAATCCCTTAACGTGAGTTTTCGTTCCACTGAGCGTCAGACCCCGTAGAAAAGATCAAAGGATCTTCTTGAGATCCTTTTTTTCTGC
GCGTAATCTGCTGCTTGCAAACAAAAAAACCACCGCTACCAGCGGTGGTTTGTTTGCCGGATCAAGAGCTACCAACTCTTTTTCCGAAG
GTAACTGGCTTCAGCAGAGCGCAGATACCAAATACTGTTCTTCTAGTGTAGCCGTAGTTAGGCCACCACTTCAAGAACTCTGTAGCACC
GCCTACATACCTCGCTCTGCTAATCCTGTTACCAGTGGCTGCTGCCAGTGGCGATAAGTCGTGTCTTACCGGGTTGGACTCAAGACGAT
AGTTACCGGATAAGGCGCAGCGGTCGGGCTGAACGGGGGGTTCGTGCACACAGCCCAGCTTGGAGCGAACGACCTACACCGAACTGAGA
TACCTACAGCGTGAGCTATGAGAAAGCGCCACGCTTCCCGAAGGGAGAAAGGCGGACAGGTATCCGGTAAGCGGCAGGGTCGGAACAGG
AGAGCGCACGAGGGAGCTTCCAGGGGGAAACGCCTGGTATCTTTATAGTCCTGTCGGGTTTCGCCACCTCTGACTTGAGCGTCGATTTT
TGTGATGCTCGTCAGGGGGGCGGAGCCTATGGAAAAACGCCAGCAACGCGGCCTTTTTACGGTTCCTGGCCTTTTGCTGGCCTTTTGCT
CACATGTTCTTTCCTGCGTTATCCCCTGATTCTGTGGATAACCGTATTACCGCCTTTGAGTGAGCTGATACCGCTCGCCGCAGCCGAAC
GACCGAGCGCAGCGAGTCAGTGAGCGAGGAAGCGGAAGA
