# Supplementary material for: Author Correction: A selectable, plasmid-based system to generate CRISPR/Cas9 gene edited and knock-in mosquito cell lines
Source: Sci Rep. 2021 Apr 1;11:7636. doi: 10.1038/s41598-021-87189-9 (PMC8016948; doi:10.1038/s41598-021-87189-9)
Supplement: Supplementary file 1 — Supplementary Information. [file 41598_2021_87189_MOESM1_ESM.pdf]

Supplementary Information for:

A selectable, plasmid-based system to generate CRISPR/Cas9 gene edited and knock-in mosquito cell lines

Kathryn Rozen-Gagnon[1*], Soon Yi[1], Eliana Jacobson[1], Sasha Novack[1], and Charles M. Rice[1]

[1]Laboratory of Virology and Infectious Disease, The Rockefeller University, New York, NY 10065, USA

*Corresponding author: Kathryn Rozen-Gagnon. Phone (212) 327-7009. Fax (212) 327-7048. krozen@rockefeller.edu

Supplementary Methods (all references are numbered as in the main text)

CRISPR/Cas9 plasmid generation

To replace the *dme* phsp70 promoter (*hsp70Bb*[47,52]) in pDCC6 with the *Ae. aegypti* polyubiquitin promoter (*aae PUb*[15,32,54]), an *AfeI* site was introduced by site-directed mutagenesis using the QuikChange II XL kit (Agilent) and oligos RU-O-22971 and RU-O-22972. The region containing the *Afe1* site was then cloned into a clean pDCC6 background by digestion of the parental pDCC6 and pDCC6-AfeI with flanking sites *SapI/AvrII* followed by ligation, transformation, and DNA isolation. *Aae* Pub with flanking *AfeI/AvrII* sites was amplified using RU-O-22977 and RU-O-22978 and Phusion polymerase (NEB) from the plasmid pSL1180-HR-PUbECFP[15,32] (a gift from Leslie Vosshall; Addgene plasmid # 47917; http://n2t.net/addgene:47917; RRID:Addgene_47917). The resulting plasmid, pKRG2 (pKRG2-dU6-PUb-3xFLAG-hSpCas9), contains the *dme* U6-2 promoter (*Drosophila* Pol III promoter *U6:96Ab*[47,48]) and the *aae* PUb promoter driving expression of hSpCas9.

To replace the *dme* U6-2 promoter with the *Aae. aegypti* U6 promoter (*aae* U6; *AAEL017774*[57]), we had to alter the sgRNA cloning sites due to an internal *BbsI* site in the *aae* U6. We designed primers to add an overhang corresponding to the *aae* U6 to a modified sgRNA cloning site that relies on *BsmBI* to the pKRG2 sgRNA tracrRNA (trans-activating CRISPR RNA) scaffold and terminator sequence, with a downstream *Afe*I site (RU-O-22974 and RU-O-22975). The scaffold PCR and a gBlocks Gene Fragment containing the *aae* U6 sequence and an upstream *SacI* site were assembled by Gibson assembly. The assembled DNA was PCR amplified using primers RU-O-22975 and RU-O-22976. The *aae* U6 insert and pKRG2 were digested with *SacI/AfeI*, ligated, and DNA was isolated to obtain pKRG3-mU6-PUb-3xFLAG-hSpCas9, which contains the *aae* U6 promoter driving sgRNA expression and the *aae* PUb promoter driving expression of hSpCas9.

We additionally generated a version of this plasmid with the 3xFLAG at the beginning of the Cas9 removed. To remove the 3xFLAG, we introduced a *NcoI* site by site-directed mutagenesis using oligos RU-O-23100 and RU-O-23101. We then cloned this mutagenized insert into a clean pKRG3 background by restriction enzyme digest with *BglII/XhoI*. The pKRG3-NcoI plasmid was then digested with *NcoI* to remove the 3xFLAG and re-ligated to generate pKRG3-mU6-PUb-hSpCas9. We made another variation with the puromycin resistance cassette (pAc) added. We amplified the end of Cas9, the intervening T2A sequence[59], and pAc from pAc-sgRNA-Cas9[30] (a gift from Ji-Long Liu; Addgene plasmid #49330; http://n2t.net/addgene:49330; RRID:Addgene_49330) using primers RU-O-23782 and RU-O-23783. We then digested pKRG3 with *Eag1/BsrGI* and generated pKRG3-mU6-PUb-hSpCas9-pAc by Gibson assembly. For comparative purposes, we also removed the 3xFLAG and added the pAc to hSpCas9 in pKRG3 by the same method, generating pKRG2-dU6-PUb-hSpCas9-pAc.

3xFLAG-tagged AGO1 plasmid generation

To generate overexpression plasmids as positive controls for mosquito AGO1 immunoblotting, the pKRG3 plasmid was further modified. Aag2 N-terminal 3xFLAG-tagged short and long AGO1 isoforms and the U4.4 N-terminal 3xFLAG-tagged AGO1 with pKRG3 plasmid overhangs were PCR-amplified from an in-house plasmid containing experimentally validated AGO1 sequences in each cell line (unpublished data). The hSpCas9 sequence was removed from pKRG3-mU6-PUb-hSpCas9-pAc by digestion with *NcoI/BsrGI* and AGO1 sequences were inserted by Gibson assembly. Alternatively, to generate an empty pKRG3 plasmid, the ends of the *NcoI/BsrGI*-digested plasmid were filled with T4 DNA polymerase (NEB) according to the manufacturer's protocol and blunt ends were re-ligated. Finally, the *aae* U6 sequence was removed from pKRG3 Ago-containing or empty plasmids by digestion with *SapI/AfeII*; the ends were filled with T4 DNA polymerase and blunt ends were ligated. This generated an empty, all-purpose pKRG4-mPUb-pAc plasmid, as well as pKRG4-mPUb-3xFLAG-Aag2-AGO1-short-pAc, pKRG4-mPUb-3xFLAG-Aag2-AGO1-long-pAc, and pKRG4-mPUb-3xFLAG-U44-AGO1-pAc. To express Cre recombinase for excision of the fluorescent reporter between the loxP sites, Cre recombinase was amplified from pME66 (a gift from S. Sarbanes) using primers adding *SacI/AvrII* sites; the Cre insert and pKRG4-mPUb-pAc were digested with *SacI/AvrII* to generate pKRG4-mPUb-Cre-pAc.

CRISPR guide RNA design and cloning

We designed CRISPR RNAs (crRNAs) corresponding to *AGO1*. For Aag2 cells, we used the *Ae. aegypti* AaegL3 genome assembly, AaegL3.3 annotations, *AAEL012410* (at the time of the design this was the most updated

assembly; since that time the AaegL5 genome assembly has been released[23]; the *AGO1* gene ID remains the same and the updated AaegL5.2 gene annotation contains the correct *AGO1* transcriptional start site). For U4.4 cells, we used the *Ae. albopictus* AaloF1 assembly, AaloF1.2 annotations, *AALF020776.* We first confirmed the genomic sequence around the experimentally determined translational start site in each cell line (unpublished data from 5'RACE and cDNA sequencing). Aag2 and U4.4 cell genomic DNA was isolated using the DNeasy Blood & Tissue Kit (Qiagen) according to the manufacturer's protocol. Aag2 genomic DNA was amplified using primers RU-O-22776 and RU-O-22777, designed using the *Ae. aegypti* AaegL3 assembly, which has the correct annotated starting methionine. U4.4 genomic DNA was amplified using primers RU-O-22929 and RU-O-22931, designed using the *Ae. albopictus* AaloF1 assembly, where we could only identify a downstream methionine in 5'RACE experiments. Three guide oligos containing the *BsmBI* overhangs in pKRG3 plasmids were designed for each cell line based on protospacer adjacent motif (PAM) NGG sequences in close proximity to the starting methionine (RU-O-23427 to RU-O-23434, *Ae. aegypti*; RU-O-23456 to RU-O-23463, *Ae. albopictus*). The parent pKRG3-mU6-PUb-hSpCas9-pAc plasmid was digested with *BsmBI* and annealed oligos were ligated to generate 6 pKRG3 plasmids, one for each guide, according to protocols from Kistler et al., 2015 and Cornell's Stem Cell and Transgenic Core Facility (https://transgenics.vertebrategenomics.cornell.edu/genome-editing.html). These co-express the crRNA plus the tracrRNA as a single guide RNA (sgRNA), and hSpCas9.

Cloning of homology-directed repair (HDR) donor template

The pSL1180-HR-PUbECFP plasmid was used as the backbone for cloning an HDR donor template. A 2kb homology arm fragment around the translational start site of *AGO1* in Aag2 cells was amplified from Aag2 genomic DNA using oligos RU-O-24703 and RU-O-24704, to add homology with the pSL1180-HR-PUbECFP plasmid. We ordered a gBlocks Gene Fragment containing an inserted 3xFLAG-tag between the first methionine and the second amino acid of AGO1, with silent mutations to ablate the sgRNA PAM sites. The gBlock extended past *PpuMI/EagI* sites in the homology arm. Next, the homology arm was digested with *PpuMI/EagI* to drop out the central ~160 nt, generating 2 ~1kb homology fragments overlapping the gBlock. pSL1180-HR-PUbECFP was digested with *NotI/EcoRI*, dropping out the PUB-eCFP, and the fragments were assembled to generate the intermediate plasmid pSL1180-HR-Aag2-3xFLAG-AGO1. Next, pSL1180-HR-Aag2-3xFLAG-AGO1 was modified to add the loxp-PUb-RFP-loxP cassette, with overlaps corresponding to the upstream homology arm and downstream 3xFLAG-AGO1 sequence. We generated four PCRs: PCR1) 5'HA-loxP primers = RU-O-25019 and RU-O-25020, template = pSL1180-HR-Aag2-3xFLAG-AGO1 pSL1180-HR-Aag2-3xFLAG-AGO1); PCR2: loxP-PUB-RFP (primers= RU-O-25021 and RU-O-25022, template = pKRG3), PUb-RFP-loxP (primers = RU-O-25023 and RU-O-25024, template= pTRIPZ; Dharmacon), loxP-3xFLAG-3'HA (primers = RU-O-25027 and RU-O-25028, template = pSL1180-HR-Aag2-3xFLAG-AGO1). pSL1180-HR-Aag2-3xFLAG-AGO1 was digested with *KpnI/PpuMI* and the fragments were assembled by Gibson assembly to generate pSL1180-HR-Aag2-loxP-PUb-RFP-loxP-3xFLAG-AGO1.

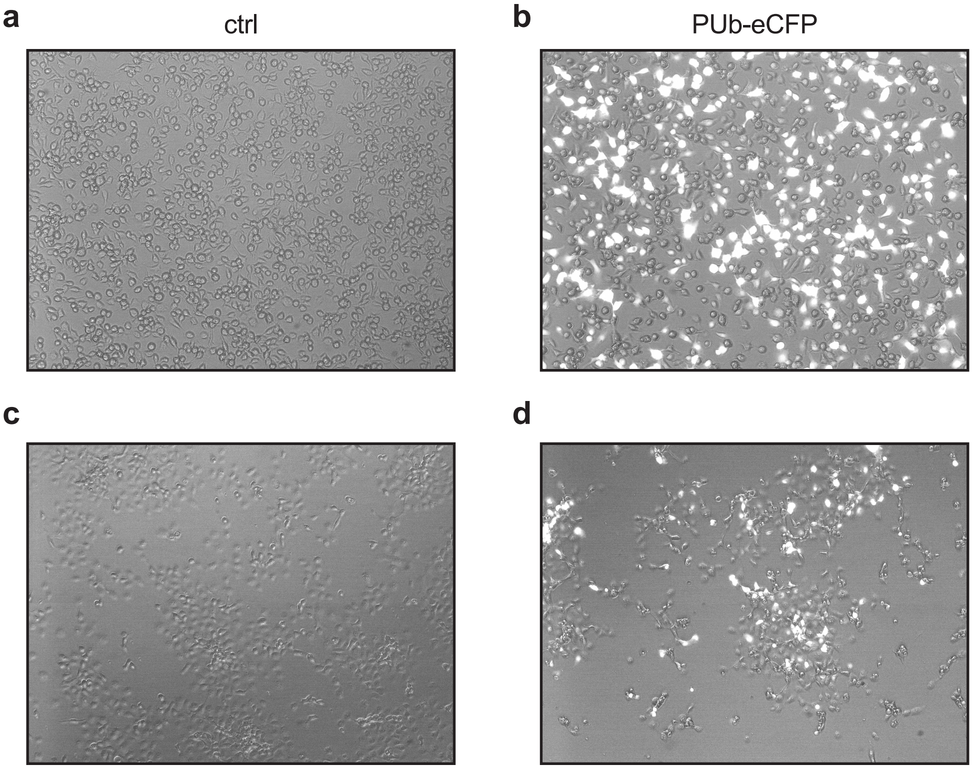

Supplementary Figure 1. Efficient transfection of mosquito cells.

(a) Representative merged brightfield and enhanced cyan flourescent protein (eCFP) image for control (ctrl) U4.4 cells treated with transfection reagent alone.

(b) As in (a), for U4.4 cells transfected with PUb-eCFP.

(c) As in (a), for Aag2 cells.

(d) As in (b), for Aag2 cells.

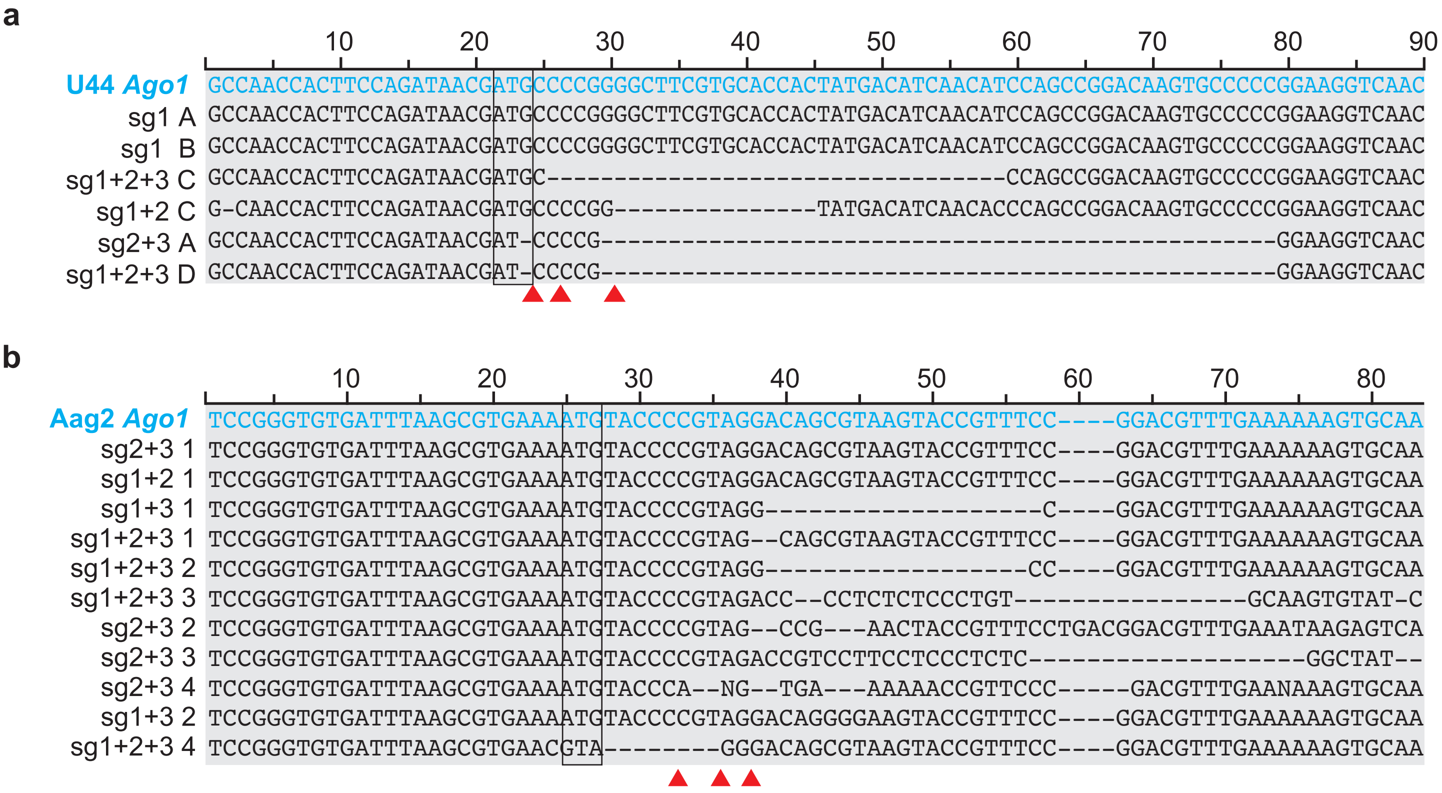

Supplementary Figure 2. *AGO1* sequences of single cell clones isolated after CRISPR/Cas9 transfection.

(a) Representative alignment of *AGO1* sequences from established U4.4 single cell clones. Clones were isolated and sequenced post-transfection with pKRG3 CRISPR/Cas9 plasmids containing guides targeting *AGO1*. sgRNA cleavage sites = red arrows; starting methionine = black box; reference sequence shown in blue.

(b) As in (a), for Aag2 cells.

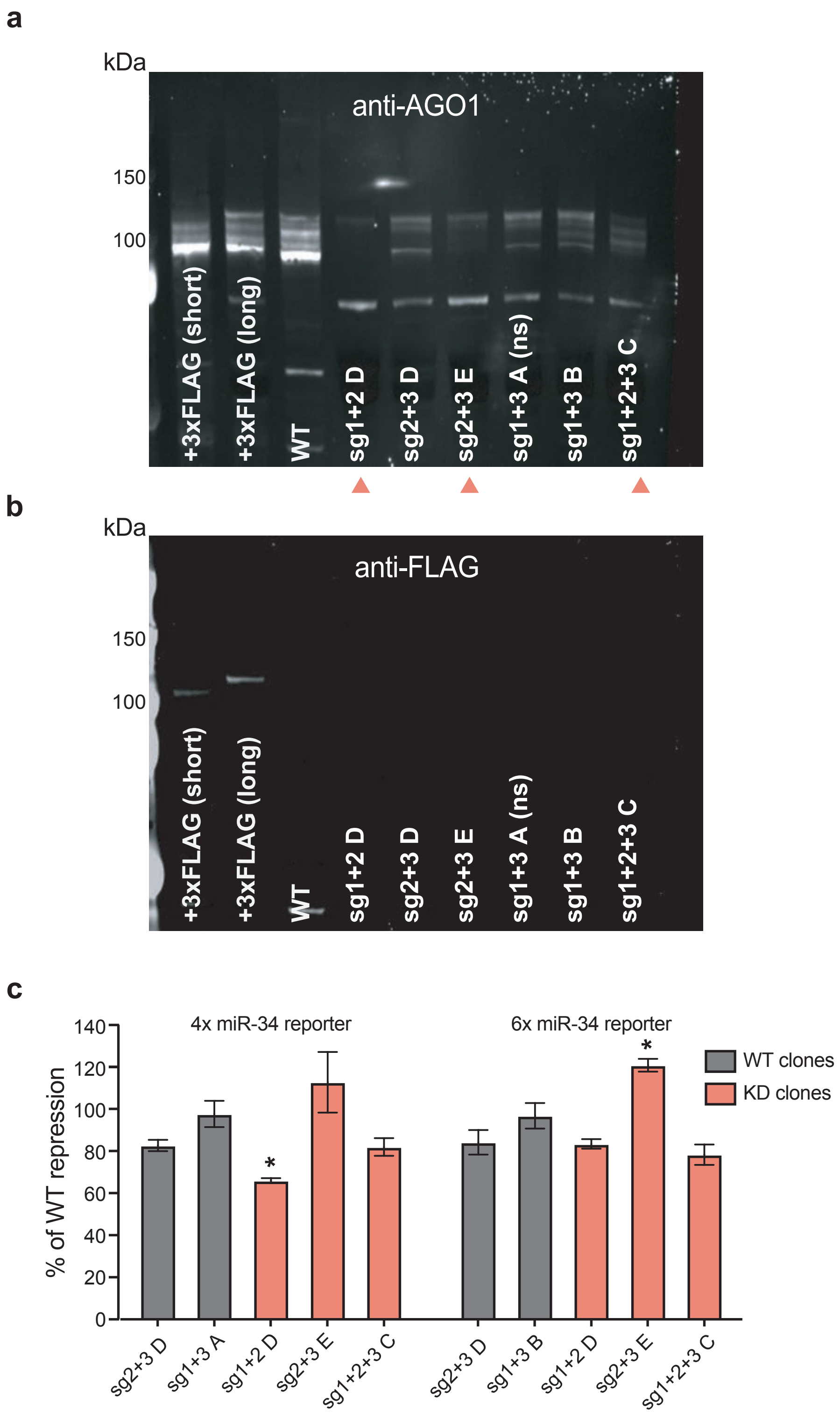

Supplementary Figure 3. AGO1 protein levels and function in Aag2 clones.

(a) Immunoblot of AGO1 showed Aag2 clones with wild-type (WT) and reduced (salmon arrows) AGO1 protein levels; A-E denote clones obtained from each sgRNA singly or in combination; ns = not shown (in reporter assay).

(b) 3xFLAG-tagged Aag2 AGO1 short and long isoforms were expressed and were detected by both the anti-AGO1 antibody in (a) and the anti-FLAG antibody.

(c) Luciferase reporter assay measuring miR-34 mediated repression of 4 or 6 repeated miR-34 sites (4x, 6x miR-34 reporter). Normalization was performed as in Fig. 3e. The percent (%) of repression compared to WT clones sg2+3 D and sg1+3 A is shown. $P < 0.0001$ (overall ANOVA comparing different clones); individual groups were compared using the Dunnett's *post hoc* test compared to the WT clone sg1+3 B; *$P < 0.05$.

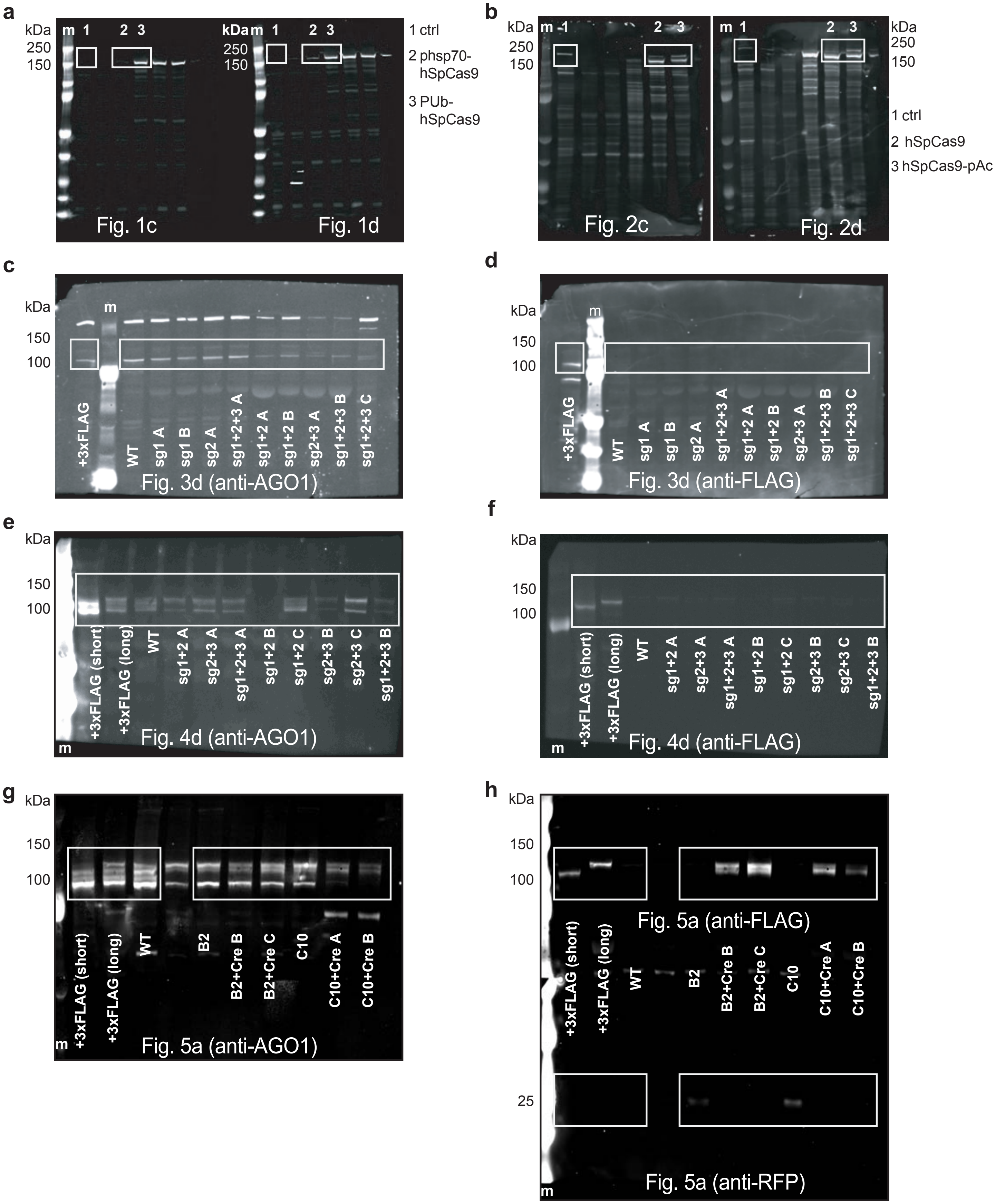

Supplementary Figure 4. Full-length blots.

(a-h) Full-length blots for all Figures. Regions included in Figures are indicated with boxes; m = marker, kDa = kilodaltons.

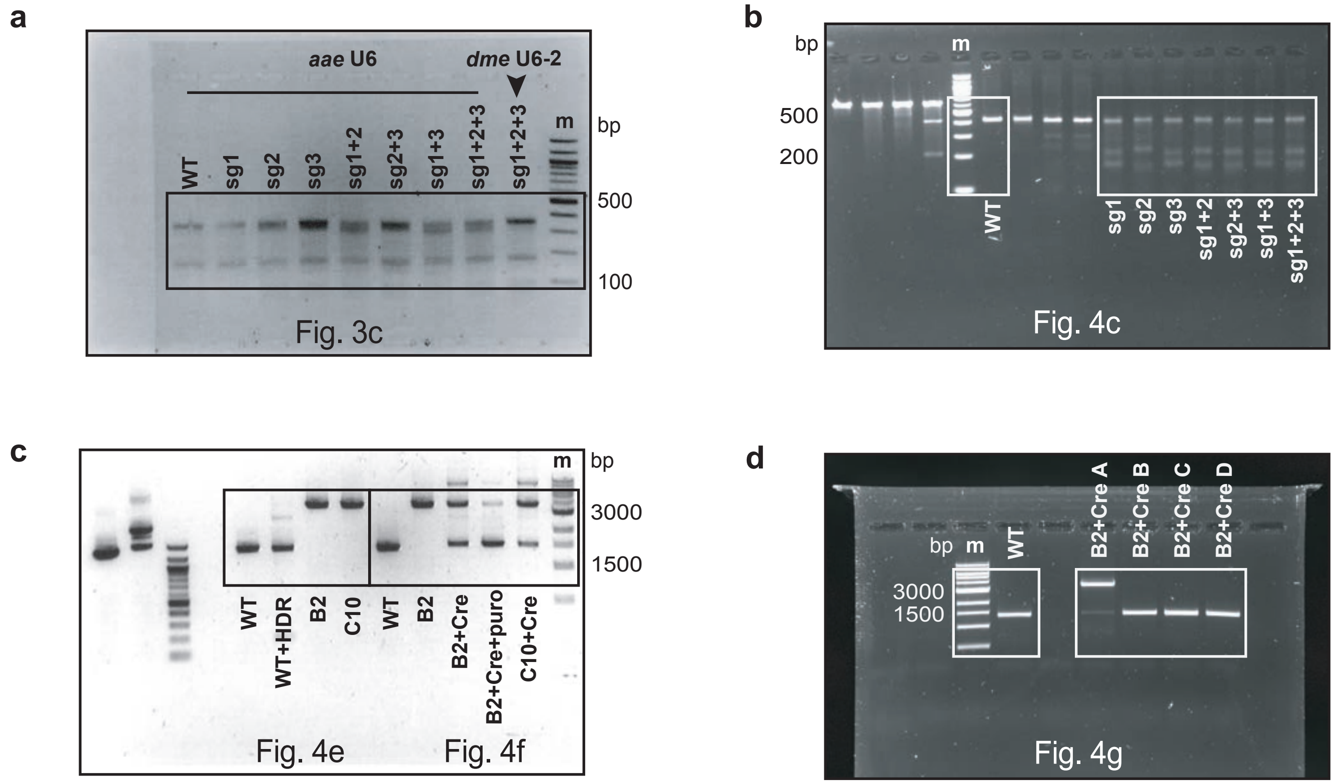

Supplementary Figure 5. Full-length gels.

(a-d) Full-length gels for all Figures. Regions included in Figures are indicated with boxes; m = marker, bp = base pairs.

Supplementary Tables

Supplementary Table 1. Sequences of all oligos used in this study.

Oligo ID	Sequence	Purpose
pKRG cloning		
RU-O-22971	CTGCAGAATTGGCGCAAGCGCTAAAAACGGACT	introduce *AfeI* pDCC6 forward; PAGE-purified
RU-O-22972	AGTCCGTTTTTAGCGCTTGCGCCAATTCTGCAG	introduce *AfeI* pDCC6 reverse; PAGE-purified
RU-O-22977	GACAGCGCTTGCGCCAATTCTGCAGACAAATGGCTATCTTTACATGTAGCTTGTGC ATTG	PUb promoter PCR forward
RU-O-22978	GTCCCTAGGTGTATACCTCCGGAAGCGCGCACTCGAGATTCGAACAAGCTTATCGA GCTTGGTGTTGAAATCTCTGTTGAGC	PUb promoter PCR reverse
RU-O-22974	GACGAAGACTATATAAGAGCAGAGGCAAGAGTAGTGAAATGGAGACGACGTCTCTG TTTTAGAGCTAGAAATAGC	tracr RNA scaffold PCR forward
RU-O-22975	GCAGAATTGGCGCAAGCGCTGTC	tracr RNA scaffold & assemble *aae* U6/scaffold PCR reverse
RU-O-22976	GACGAAGAGCGCCCAATACGCAA	assemble aae U6/scaffold PCR forward
gBLOCK *aae* U6	GAAGAGCGCCCAATACGCAAACCGCCTCTCCCCGCGGGTTGGCCGATTCATTAATG CAGGCAACTCGTGAAAGGTAGGCGGATCAGCGAATGAAATCGCCCATCGAGTTGAT ACGTCCATCCATCGCTAGAACCGCGTTCGCTGTAGAAGACTATATAAGAGCAGAGG CAAGAGTAGTGAAATGGAGACG	U6 geneblock
RU-O-23101	GATATTGATTACAAAGACGATGACGATACCATGGCCCCAAAGAAG	introduce *NcoI* remove 3xFLAG forward; PAGE-purified
RU-O-23100	CTTCTTTGGGGCCATGGTATCGTCATCGTCTTTGTAATCAATATC	introduce *NcoI* remove 3xFLAG reverse; PAGE-purified
RU-O-25485	GACCCTAGGATGGGCCCAAAGAAG	Cre into pKRG4 forward
RU-O-25486	CGGTAGAGCTCATCGCCATCTTCCAG	Cre into pKRG4 reverse
sgRNA oligos		
RU-O-23427	AAATGGTACTTACGCTGTCCTACG	pKRG Aag2 Ago1 sgRNA 1 forward
RU-O-23428	AAACCGTAGGACAGCGTAAGTACC	pKRG Aag2 Ago1 sgRNA 1 reverse
RU-O-23430	AAATGAGCGTGAAAATGTACCCCGT	pKRG Aag2 Ago1 sgRNA 2 forward
RU-O-23431	AAACACGGGGTACATTTTCACGCTC	pKRG Aag2 Ago1 sgRNA 2 reverse
RU-O-23433	AAATGACGGTACTTACGCTGTCCTA	pKRG Aag2 Ago1 sgRNA 3 forward
RU-O-23434	AAACTAGGACAGCGTAAGTACCGTC	pKRG Aag2 Ago1 sgRNA 3 reverse
RU-O-23456	AAATGTAGTGGTGCACGAAGCCCCG	pKRG U4.4 Ago1 sgRNA 1 forward
RU-O-23457	AAACCGGGGCTTCGTGCACCACTAC	pKRG U4.4 Ago1 sgRNA 1 reverse
RU-O-23459	AAATGTTCCAGATAACGATGCCCCG	pKRG U4.4 Ago1 sgRNA 2 forward
RU-O-23460	AAACCGGGGCATCGTTATCTGGAAC	pKRG U4.4 Ago1 sgRNA 2 reverse
RU-O-23462	AAATGACTTCCAGATAACGATGCCC	pKRG U4.4 Ago1 sgRNA 3 forward
RU-O-23463	AAACGGGCATCGTTATCTGGAAGTC	pKRG U4.4 Ago1 sgRNA 3 reverse
HDR donor template oligos		
RU-O-24703	GACCATGATTACGAATTCGACATGTAGGACATGTGGGG	Aag2 HA PCR forward
RU-O-24704	CCAGCTGCAGGCGGCCGCCACCGGATTTGCTTTTCGTC	Aag2 HA PCR reverse
gBLOCK Aag2 HDR donor template	CGAAAAGTGCAAAAATTCGGCCGTAATTAGTTCGCTTCCGTTTTTCCGAGTGCCTC CGATCGTTAGCGGACCGTTCCGGGTGTGATTTAAGCGTGAAAATGGATTACAAGGA TCACGATGGAGATTACAAGGATCACGATATCGATTACAAGGATGATGATAAGTATC CGGTGGGTCAACGTAAGTACCGTTTCCGGACGTTTGAAAAAGTGCAATTCACAAG AGAAGAAAAAAAAAGTGTTGACGAGGGGACCTTCCTTCCTCCCTC	
RU-O-25019	GGAGCGTCCGTAAAATGAAA	*Kpn1* HA overlap forward
RU-O-25020	ATAACTTCGTATAGCATACATTATACGAAGTTATTTTCACGCTTAAATC	5'HA add loxP reverse
RU-O-25021	GCTATACGAAGTTATTATCTTTACATGTAG	loxP-PUb forward
RU-O-25022	GATCAGCTCGCTCATGCGGCCGCGGTAGAGCTCCGAATTCAC	pUB-RFP reverse
RU-O-25023	ATGAGCGAGCTGATC	RFP forward
RU-O-25024	CATTATACGAAGTTATTAATTAATTATCTGTGCCCCAG	RFP-loxP reverse
RU-O-25027	ATAACTTCGTATAATGTATGCTATACGAAGTTATATGGATTACAAGGATC	loxP-HA forward
RU-O-25028	AGAGACGAGAGGGAGGAAGG	HA *PpuMI* overlap reverse
Sequencing, surveyor & integration PCRs		
RU-O-26075	ACTTGTCTCACTCGCCATCATACG	Aag2 HDR PCR F
RU-O-26076	CGATCAGATGCGCACGAAAACT	Aag2 HDR PCR R
RU-O-22776	ACTTGTCTCACTCGCCATCA	Aag2 surveyor/sequencing forward
RU-O-22777	GTCCGTACACAGGAAAAGCC	Aag2 surveyor/sequencing reverse
RU-O-22929	GCCAACCACTTCCAGATAACG	U4.4 surveyor forward
RU-O-24042	AGGATGGCATCGTACGGAAT	U4.4 surveyor reverse
RU-O-22930	TCTACCGGTGTTCACCTGTC	U4.4 sequencing forward
RU-O-22931	TGATCTCCCGGTTGACCTTC	U4.4 sequencing reverse
Reporter cloning oligos		
RU-O-24800	TCGAGCAACCAGCTAGGCCACTGCCACCGGCAACCAGCTAGGCCACTGCCACCGGC AACCAGCTAGGCCACTGCCACCGGCAACCAGCTAGGCCACTGCCAGC	Aag2 miR-34-5p 4x ideal psiCHECK2 forward
RU-O-24801	GGCCGCTGGCAGTGGCCTAGCTGGTTGCCGGTGGCAGTGGCCTAGCTGGTTGCCGG TGGCAGTGGCCTAGCTGGTTGCCGGTGGCAGTGGCCTAGCTGGTTGC	Aag2 miR-34-5p 4x ideal psiCHECK2 reverse
RU-O-24794	TCGAGCAACCAGCTAACCACACTGCCAGC	Aag2 miR-34-5p perfect psiCHECK2 forward
RU-O-24795	GGCCGCTGGCAGTGTGGTTAGCTGGTTGC	Aag2 miR-34-5p perfect psiCHECK2 reverse

HA = homology arm; HDR = homology-directed repair; tracr = trans-activating CRISPR; sgRNA = single-guide RNA.
All oligos were ordered in standard desalted format from IDT, unless indicated otherwise.

pKRG3-mU6-PUb-3xFLAG-hSpCas9

aae U6
BsmBI sites for sgRNA cloning
sgRNA tracrRNA and U6 terminator
aae PUb promoter
3xFLAG
NLS
hSpCas9

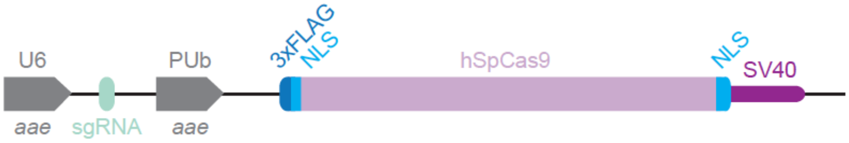

GCGCCCAATACGCAAACCGCCTCTCCCCGCGGGTTGGCCGATTCATTAATGCAGGCAACTCGTGAAAGGTAGGCGGATCAGCGAATGAA
ATCGCCCATCGAGTTGATACGTCCATCCATCGCTAGAACCGCGTTCGCTGTAGAAGACTATATAAGAGCAGAGGCAAGAGTAGTGAAAT
gGAGACGaCGTCTCtGTTTTAGAGCTAGAAATAGCAAGTTAAAATAAGGCTAGTCCGTTATCAACTTGAAAAAGTGGCACCGAGTCGGT
GCTTTTTTGTTTTAGAGCTAGAAATAGCAAGTTAAAATAAGGCTAGTCCGTTTTTAGCGCTTGCGCCAATTCTGCAGACAAATGGCTAT
CTTTACATGTAGCTTGTGCATTGAATCCAATTATAATTTGCCTTGGCACCAGCTGAGCCAGACAAGAAAGAAAGCTTCCCAGAAGTATA
TCGATTTAGAAGGGTTGACGTCACTTGCTGACTGCACTAATACAGCAAATGATGCAATTAGAATGATTCAAGTGAAATTCCCAAATTAC
TGATTTTTCTCTGGATTTGGTTATCAGATTACATTCGAAGCTAAGATTAGCTACCGAAATTGTCGATCAAATCAGGAAATCCTTTCTCT
ATCGAAAAAGGCATTCGCACATCTTCCTCTCTATGCCATATACACGAAGGGTAGGTACATTGACGTCTTTGCCAGAAGTTGAACTGCAT
CGTTCAAGGTACAGAATGAACGACTAACAGACACAAGCACGTTTTGCTGTCCATTCAGACACAGGGATGGTACCCATATTCGATCGATA
TAGAGCCATCCAACCGAACAGAGGTATATGTATGAATGTATTGCTGAAATTTTCTAGAAGTACAACCACCACTACGACAGTGTCTATAA
AACGCCCCTGCAAAGGCGAAACCAGCTCAATCGAATACGTTTCCTAGTGGAGTGAACATTACGCGGCCCAAGTAAGCAGTGCCAGTGCA
AGTGAAGTGAAGTCTCTAGTGAAAAAGAGTGATCCAATTAGCCAGAGGAGAAAATTTCAGAGTGAACAAAGCTTTATTCAAAGGACAAT
TACTATTAAATTGGTGAAAGTGCATTTCGGTGAAGGGAATCTTCTAGTGAAGGTAGGTAAATTAATTGATGAAATTATAGCTATGAGCG
AAAACTAGTTTGGTGAATGATTCCTTTGTCTTTGAATGAGCAAACTATTTTCCAAGATGGCGACTATTGAGCTTTGAGTGATTAGTGAA
AATTTGCAACGCAGTTTCATCATCATTGATAAAACCCAATTGTGATTCACAGCGATAATCATATTTCGTTGAATCATCGCTACTAATTG
AATTAAATTTCTAGAATAATAAGAATAACGTATTTGCTCCGTCACATATCTAAAATAAATATTTTGATGGTAATTACCCATTAAGGTAA
TATTAACACATATCGAGAAAAACCTTGAGGAAATCGTGAAAACTTGAAGATACGCAATTTCAAAACTACGTAGTTCAAAGTCGAAAACA
AGTTAATTTTTCACTTAAAAGTAGGGCGTTGTTGTGACGTCATCACCTTCAAGTGTATATTTTTCACTTGGCCTGCGACTGCAAACGCA
GACAAAGCAAAACAAGTTTAAAACCTGTCGTGTCGTGCTCGAAGCCAAAGGCAATGAATCAATATCAAATGAGAGTTTGCATTTCACAA
CCAATTACTCAAGCGTTTCCTCGTTTCTTTTTCTGCTCAACAGAGATTTCAACACCAAGCTCGATAAGCTTGTTCGAATCTCGAGTGCG
CGCTTCCGGAGGTATACACCTAGGCGGTACCACTGCAGTGAATTCGGAGCTCTACCGGTGCCACCATGGACTATAAGGACCACGACGGA
GACTACAAGGATCATGATATTGATTACAAAGACGATGACGATAAGATGGCCCCAAAGAAGAAGCGGAAGGTCGGTATCCACGGAGTCCC
AGCAGCCGACAAGAAGTACAGCATCGGCCTGGACATCGGCACCAACTCTGTGGGCTGGGCCGTGATCACCGACGAGTACAAGGTGCCCA
GCAAGAAATTCAAGGTGCTGGGCAACACCGACCGGCACAGCATCAAGAAGAACCTGATCGGAGCCCTGCTGTTCGACAGCGGCGAAACA
GCCGAGGCCACCCGGCTGAAGAGAACCGCCAGAAGAAGATACACCAGACGGAAGAACCGGATCTGCTATCTGCAAGAGATCTTCAGCAA
CGAGATGGCCAAGGTGGACGACAGCTTCTTCCACAGACTGGAAGAGTCCTTCCTGGTGGAAGAGGATAAGAAGCACGAGCGGCACCCCA
TCTTCGGCAACATCGTGGACGAGGTGGCCTACCACGAGAAGTACCCCACCATCTACCACCTGAGAAAGAAACTGGTGGACAGCACCGAC
AAGGCCGACCTGCGGCTGATCTATCTGGCCCTGGCCCACATGATCAAGTTCCGGGGCCACTTCCTGATCGAGGGCGACCTGAACCCCGA
CAACAGCGACGTGGACAAGCTGTTCATCCAGCTGGTGCAGACCTACAACCAGCTGTTCGAGGAAAACCCCATCAACGCCAGCGGCGTGG
ACGCCAAGGCCATCCTGTCTGCCAGACTGAGCAAGAGCAGACGGCTGGAAAATCTGATCGCCCAGCTGCCCGGCGAGAAGAAGAATGGC
CTGTTCGGAAACCTGATTGCCCTGAGCCTGGGCCTGACCCCCAACTTCAAGAGCAACTTCGACCTGGCCGAGGATGCCAAACTGCAGCT
GAGCAAGGACACCTACGACGACGACCTGGACAACCTGCTGGCCCAGATCGGCGACCAGTACGCCGACCTGTTTCTGGCCGCCAAGAACC
TGTCCGACGCCATCCTGCTGAGCGACATCCTGAGAGTGAACACCGAGATCACCAAGGCCCCCCTGAGCGCCTCTATGATCAAGAGATAC
GACGAGCACCACCAGGACCTGACCCTGCTGAAAGCTCTCGTGCGGCAGCAGCTGCCTGAGAAGTACAAAGAGATTTTCTTCGACCAGAG
CAAGAACGGCTACGCCGGCTACATTGACGGCGGAGCCAGCCAGGAAGAGTTCTACAAGTTCATCAAGCCCATCCTGGAAAAGATGGACG
GCACCGAGGAACTGCTCGTGAAGCTGAACAGAGAGGACCTGCTGCGGAAGCAGCGGACCTTCGACAACGGCAGCATCCCCCACCAGATC
CACCTGGGAGAGCTGCACGCCATTCTGCGGCGGCAGGAAGATTTTTACCCATTCCTGAAGGACAACCGGGAAAAGATCGAGAAGATCCT
GACCTTCCGCATCCCCTACTACGTGGGCCCTCTGGCCAGGGGAAACAGCAGATTCGCCTGGATGACCAGAAAGAGCGAGGAAACCATCA
CCCCCTGGAACTTCGAGGAAGTGGTGGACAAGGGCGCTTCCGCCCAGAGCTTCATCGAGCGGATGACCAACTTCGATAAGAACCTGCCC
AACGAGAAGGTGCTGCCCAAGCACAGCCTGCTGTACGAGTACTTCACCGTGTATAACGAGCTGACCAAAGTGAAATACGTGACCGAGGG
AATGAGAAAGCCCGCCTTCCTGAGCGGCGAGCAGAAAAAGGCCATCGTGGACCTGCTGTTCAAGACCAACCGGAAAGTGACCGTGAAGC
AGCTGAAAGAGGACTACTTCAAGAAAATCGAGTGCTTCGACTCCGTGGAAATCTCCGGCGTGGAAGATCGGTTCAACGCCTCCCTGGGC
ACATACCACGATCTGCTGAAAATTATCAAGGACAAGGACTTCCTGGACAATGAGGAAAACGAGGACATTCTGGAAGATATCGTGCTGAC
CCTGACACTGTTTGAGGACAGAGAGATGATCGAGGAACGGCTGAAAACCTATGCCCACCTGTTCGACGACAAAGTGATGAAGCAGCTGA
AGCGGCGGAGATACACCGGCTGGGGCAGGCTGAGCCGGAAGCTGATCAACGGCATCCGGGACAAGCAGTCCGGCAAGACAATCCTGGAT
TTCCTGAAGTCCGACGGCTTCGCCAACAGAAACTTCATGCAGCTGATCCACGACGACAGCCTGACCTTTAAAGAGGACATCCAGAAAGC
CCAGGTGTCCGGCCAGGGCGATAGCCTGCACGAGCACATTGCCAATCTGGCCGGCAGCCCCGCCATTAAGAAGGGCATCCTGCAGACAG
TGAAGGTGGTGGACGAGCTCGTGAAAGTGATGGGCCGGCACAAGCCCGAGAACATCGTGATCGAAATGGCCAGAGAGAACCAGACCACC
CAGAAGGGACAGAAGAACAGCCGCGAGAGAATGAAGCGGATCGAAGAGGGCATCAAAGAGCTGGGCAGCCAGATCCTGAAAGAACACCC
CGTGGAAAACACCCAGCTGCAGAACGAGAAGCTGTACCTGTACTACCTGCAGAATGGGCGGGATATGTACGTGGACCAGGAACTGGACA
TCAACCGGCTGTCCGACTACGATGTGGACCATATCGTGCCTCAGAGCTTTCTGAAGGACGACTCCATCGACAACAAGGTGCTGACCAGA
AGCGACAAGAACCGGGGCAAGAGCGACAACGTGCCCTCCGAAGAGGTCGTGAAGAAGATGAAGAACTACTGGCGGCAGCTGCTGAACGC
CAAGCTGATTACCCAGAGAAAGTTCGACAATCTGACCAAGGCCGAGAGAGGCGGCCTGAGCGAACTGGATAAGGCCGGCTTCATCAAGA
GACAGCTGGTGGAAACCCGGCAGATCACAAAGCACGTGGCACAGATCCTGGACTCCCGGATGAACACTAAGTACGACGAGAATGACAAG

CTGATCCGGGAAGTGAAAGTGATCACCCTGAAGTCCAAGCTGGTGTCCGATTTCCGGAAGGATTTCCAGTTTTACAAAGTGCGCGAGAT
CAACAACTACCACCACGCCCACGACGCCTACCTGAACGCCGTCGTGGGAACCGCCCTGATCAAAAAGTACCCTAAGCTGGAAAGCGAGT
TCGTGTACGGCGACTACAAGGTGTACGACGTGCGGAAGATGATCGCCAAGAGCGAGCAGGAAATCGGCAAGGCTACCGCCAAGTACTTC
TTCTACAGCAACATCATGAACTTTTTCAAGACCGAGATTACCCTGGCCAACGGCGAGATCCGGAAGCGGCCTCTGATCGAGACAAACGG
CGAAACCGGGGAGATCGTGTGGGATAAGGGCCGGGATTTTGCCACCGTGCGGAAAGTGCTGAGCATGCCCCAAGTGAATATCGTGAAAA
AGACCGAGGTGCAGACAGGCGGCTTCAGCAAAGAGTCTATCCTGCCCAAGAGGAACAGCGATAAGCTGATCGCCAGAAAGAAGGACTGG
GACCCTAAGAAGTACGGCGGCTTCGACAGCCCCACCGTGGCCTATTCTGTGCTGGTGGTGGCCAAAGTGGAAAAGGGCAAGTCCAAGAA
ACTGAAGAGTGTGAAAGAGCTGCTGGGGATCACCATCATGGAAAGAAGCAGCTTCGAGAAGAATCCCATCGACTTTCTGGAAGCCAAGG
GCTACAAAGAAGTGAAAAAGGACCTGATCATCAAGCTGCCTAAGTACTCCCTGTTCGAGCTGGAAAACGGCCGGAAGAGAATGCTGGCC
TCTGCCGGCGAACTGCAGAAGGGAAACGAACTGGCCCTGCCCTCCAAATATGTGAACTTCCTGTACCTGGCCAGCCACTATGAGAAGCT
GAAGGGCTCCCCCGAGGATAATGAGCAGAAACAGCTGTTTGTGGAACAGCACAAGCACTACCTGGACGAGATCATCGAGCAGATCAGCG
AGTTCTCCAAGAGAGTGATCCTGGCCGACGCTAATCTGGACAAAGTGCTGTCCGCCTACAACAAGCACCGGGATAAGCCCATCAGAGAG
CAGGCCaAGAATATCATCCACCTGTTTACCCTGACCAATCTGGGAGCCCCTGCCGCCTTCAAGTACTTTGACACCACCATCGACCGGAA
GAGGTACACCAGCACCAAAGAGGTGCTGGACGCCACCCTGATCCACCAGAGCATCACCGGCCTGTACGAGACACGGATCGACCTGTCTC
AGCTGGGAGGCGACAGCCCCAAGAAGAAGAGAAAGGTGGAGGCCAGCTAATAGGACCCAGCTTTCTTGTACAAAGTGGTGACGTAAGCT
AGCAGGATCTTTGTGAAGGAACCTTACTTCTGTGGTGTGACATAATTGGACAAACTACCTACAGAGATTTAAAGCTCTAAGGTAAATAT
AAAATTTTTAAGTGTATAATGTGTTAAACTACTGATTCTAATTGTTTGTGTATTTTAGATTCCAACCTATGGAACTGATGAATGGGAGC
AGTGGTGGAATGCCTTTAATGAGGAAAACCTGTTTTGCTCAGAAGAAATGCCATCTAGTGATGATGAGGCTACTGCTGACTCTCAACAT
TCTACTCCTCCAAAAAAGAAGAGAAAGGTAGttGACCCCAAGGACTTTCCTTCAGAATTGCTAAGTTTTTTGAGTCATGCTGTGTTTAG
TAATAGAACTCTTGCTTGCTTTGCTATTTACACCACAAAGGAAAAAGCTGCACTGCTATACAAGAAAATTATGGAAAAATATTCTGTAA
CCTTTATAAGTAGGCATAACAGTTATAATCATAACATACTGTTTTTTCTTACTCCACACAGGCATAGAGTGTCTGCTATTAATAACTAT
GCTCAAAAATTGTGTACCTTTAGCTTTTTAATTTGTAAAGGGGTTAATAAGGAATATTTGATGTATAGTGCCTTGACTAGAGATCATAA
TCAGCCATACCACATTTGTAGAGGTTTTACTTGCTTTAAAAAACCTCCCACACCTCCCCCTGAACCTGAAACATAAAATGAATGCAATT
GTTGTTGTTAACTTGTTTATTGCAGCTTATAATGGTTACAAATAAAGCAATAGCATCACAAATTTCACAAATAAAGCATTTTTTTCACT
GCATTCTAGTTGTGGTTTGTCCAAACTCATCAATGTATCTTATCATGTCTGGATCCCGTTTAAACTACGCGTAATTCAAACAGGGTTCT
GGCGTCGTTCTCGTACTGTTTTCCCCAGGCCAGTGCTTTAGCGTTATTGAAAAAGGAAGAGTATGAGTATTCAACATTTCCGTGTCGCC
CTTATTCCCTTTTTTGCGGCATTTTGCCTTCCTGTTTTTGCTCACCCAGAAACGCTGGTGAAAGTAAAAGATGCTGAAGATCAGTTGGG
TGCACGAGTGGGTTACATCGAACTGGATCTCAACAGCGGTAAGATCCTTGAGAGTTTTCGCCCCGAAGAACGTTTTCCAATGATGAGCA
CTTTTAAAGTTCTGCTATGTGGCGCGGTATTATCCCGTATTGACGCCGGGCAAGAGCAACTCGGTCGCCGCATACACTATTCTCAGAAT
GACTTGGTTGAGTACTCACCAGTCACAGAAAAGCATCTTACGGATGGCATGACAGTAAGAGAATTATGCAGTGCTGCCATAACCATGAG
TGATAACACTGCGGCCAACTTACTTCTGACAACGATCGGAGGACCGAAGGAGCTAACCGCTTTTTTGCACAACATGGGGGATCATGTAA
CTCGCCTTGATCGTTGGGAACCGGAGCTGAATGAAGCCATACCAAACGACGAGCGTGACACCACGATGCCTGTAGCAATGGCAACAACG
TTGCGCAAACTATTAACTGGCGAACTACTTACTCTAGCTTCCCGGCAACAATTAATAGACTGGATGGAGGCGGATAAAGTTGCAGGACC
ACTTCTGCGCTCGGCCCTTCCGGCTGGCTGGTTTATTGCTGATAAATCTGGAGCCGGTGAGCGTGGGTCTCGCGGTATCATTGCAGCAC
TGGGGCCAGATGGTAAGCCCTCCCGTATCGTAGTTATCTACACGACGGGGAGTCAGGCAACTATGGATGAACGAAATAGACAGATCGCT
GAGATAGGTGCCTCACTGATTAAGCATTGGTAACTGTCAGACCAAGTTTACTCATATATACTTTAGATTGATTTAAAACTTCATTTTTA
ATTTAAAAGGATCTAGGTGAAGATCCTTTTTGATAATCTCATGACCAAAATCCCTTAACGTGAGTTTTCGTTCCACTGAGCGTCAGACC
CCGTAGAAAAGATCAAAGGATCTTCTTGAGATCCTTTTTTTCTGCGCGTAATCTGCTGCTTGCAAACAAAAAAACCACCGCTACCAGCG
GTGGTTTGTTTGCCGGATCAAGAGCTACCAACTCTTTTTCCGAAGGTAACTGGCTTCAGCAGAGCGCAGATACCAAATACTGTTCTTCT
AGTGTAGCCGTAGTTAGGCCACCACTTCAAGAACTCTGTAGCACCGCCTACATACCTCGCTCTGCTAATCCTGTTACCAGTGGCTGCTG
CCAGTGGCGATAAGTCGTGTCTTACCGGGTTGGACTCAAGACGATAGTTACCGGATAAGGCGCAGCGGTCGGGCTGAACGGGGGGTTCG
TGCACACAGCCCAGCTTGGAGCGAACGACCTACACCGAACTGAGATACCTACAGCGTGAGCTATGAGAAAGCGCCACGCTTCCCGAAGG
GAGAAAGGCGGACAGGTATCCGGTAAGCGGCAGGGTCGGAACAGGAGAGCGCACGAGGGAGCTTCCAGGGGGAAACGCCTGGTATCTTT
ATAGTCCTGTCGGGTTTCGCCACCTCTGACTTGAGCGTCGATTTTTGTGATGCTCGTCAGGGGGGCGGAGCCTATGGAAAAACGCCAGC
AACGCGGCCTTTTTACGGTTCCTGGCCTTTTGCTGGCCTTTTGCTCACATGTTCTTTCCTGCGTTATCCCCTGATTCTGTGGATAACCG
TATTACCGCCTTTGAGTGAGCTGATACCGCTCGCCGCAGCCGAACGACCGAGCGCAGCGAGTCAGTGAGCGAGGAAGCGGAAGA

pKRG3-mU6-PUb-hSpCas9

aae U6
BsmBI sites for sgRNA cloning
sgRNA tracrRNA and U6 terminator
aae PUb promoter
NLS
hSpCas9

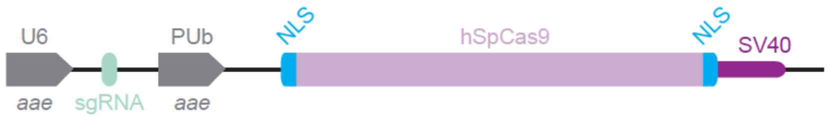

GCGCCCAATACGCAAACCGCCTCTCCCCGCGGGTTGGCCGATTCATTAATGCAGGCAACTCGTGAAAGGTAGGCGGATCAGCGAATGAA
ATCGCCCATCGAGTTGATACGTCCATCCATCGCTAGAACCGCGTTCGCTGTAGAAGACTATATAAGAGCAGAGGCAAGAGTAGTGAAAT
gGAGACGaCGTCTCtGTTTTAGAGCTAGAAATAGCAAGTTAAAATAAGGCTAGTCCGTTATCAACTTGAAAAAGTGGCACCGAGTCGGT
GCTTTTTTGTTTTAGAGCTAGAAATAGCAAGTTAAAATAAGGCTAGTCCGTTTTTAGCGCTTGCGCCAATTCTGCAGACAAATGGCTAT
CTTTACATGTAGCTTGTGCATTGAATCCAATTATAATTTGCCTTGGCACCAGCTGAGCCAGACAAGAAAGAAAGCTTCCCAGAAGTATA
TCGATTTAGAAGGGTTGACGTCACTTGCTGACTGCACTAATACAGCAAATGATGCAATTAGAATGATTCAAGTGAAATTCCCAAATTAC
TGATTTTTCTCTGGATTTGGTTATCAGATTACATTCGAAGCTAAGATTAGCTACCGAAATTGTCGATCAAATCAGGAAATCCTTTCTCT
ATCGAAAAAGGCATTCGCACATCTTCCTCTCTATGCCATATACACGAAGGGTAGGTACATTGACGTCTTTGCCAGAAGTTGAACTGCAT
CGTTCAAGGTACAGAATGAACGACTAACAGACACAAGCACGTTTTGCTGTCCATTCAGACACAGGGATGGTACCCATATTCGATCGATA
TAGAGCCATCCAACCGAACAGAGGTATATGTATGAATGTATTGCTGAAATTTTCTAGAAGTACAACCACCACTACGACAGTGTCTATAA
AACGCCCCTGCAAAGGCGAAACCAGCTCAATCGAATACGTTTCCTAGTGGAGTGAACATTACGCGGCCCAAGTAAGCAGTGCCAGTGCA
AGTGAAGTGAAGTCTCTAGTGAAAAAGAGTGATCCAATTAGCCAGAGGAGAAAATTTCAGAGTGAACAAAGCTTTATTCAAAGGACAAT
TACTATTAAATTGGTGAAAGTGCATTTCGGTGAAGGGAATCTTCTAGTGAAGGTAGGTAAATTAATTGATGAAATTATAGCTATGAGCG
AAAACTAGTTTGGTGAATGATTCCTTTGTCTTTGAATGAGCAAACTATTTTCCAAGATGGCGACTATTGAGCTTTGAGTGATTAGTGAA
AATTTGCAACGCAGTTTCATCATCATTGATAAAACCCAATTGTGATTCACAGCGATAATCATATTTCGTTGAATCATCGCTACTAATTG
AATTAAATTTCTAGAATAATAAGAATAACGTATTTGCTCCGTCACATATCTAAAATAAATATTTTGATGGTAATTACCCATTAAGGTAA
TATTAACACATATCGAGAAAAACCTTGAGGAAATCGTGAAAACTTGAAGATACGCAATTTCAAAACTACGTAGTTCAAAGTCGAAAACA
AGTTAATTTTTCACTTAAAAGTAGGGCGTTGTTGTGACGTCATCACCTTCAAGTGTATATTTTTCACTTGGCCTGCGACTGCAAACGCA
GACAAAGCAAAACAAGTTTAAAACCTGTCGTGTCGTGCTCGAAGCCAAAGGCAATGAATCAATATCAAATGAGAGTTTGCATTTCACAA
CCAATTACTCAAGCGTTTCCTCGTTTCTTTTTCTGCTCAACAGAGATTTCAACACCAAGCTCGATAAGCTTGTTCGAATCTCGAGTGCG
CGCTTCCGGAGGTATACACCTAGGCGGTACCACTGCAGTGAATTCGGAGCTCTACCGGTGCCACCATGGCCCCAAAGAAGAAGCGGAAG
GTCGGTATCCACGGAGTCCCAGCAGCCGACAAGAAGTACAGCATCGGCCTGGACATCGGCACCAACTCTGTGGGCTGGGCCGTGATCAC
CGACGAGTACAAGGTGCCCAGCAAGAAATTCAAGGTGCTGGGCAACACCGACCGGCACAGCATCAAGAAGAACCTGATCGGAGCCCTGC
TGTTCGACAGCGGCGAAACAGCCGAGGCCACCCGGCTGAAGAGAACCGCCAGAAGAAGATACACCAGACGGAAGAACCGGATCTGCTAT
CTGCAAGAGATCTTCAGCAACGAGATGGCCAAGGTGGACGACAGCTTCTTCCACAGACTGGAAGAGTCCTTCCTGGTGGAAGAGGATAA
GAAGCACGAGCGGCACCCCATCTTCGGCAACATCGTGGACGAGGTGGCCTACCACGAGAAGTACCCCACCATCTACCACCTGAGAAAGA
AACTGGTGGACAGCACCGACAAGGCCGACCTGCGGCTGATCTATCTGGCCCTGGCCCACATGATCAAGTTCCGGGGCCACTTCCTGATC
GAGGGCGACCTGAACCCCGACAACAGCGACGTGGACAAGCTGTTCATCCAGCTGGTGCAGACCTACAACCAGCTGTTCGAGGAAAACCC
CATCAACGCCAGCGGCGTGGACGCCAAGGCCATCCTGTCTGCCAGACTGAGCAAGAGCAGACGGCTGGAAAATCTGATCGCCCAGCTGC
CCGGCGAGAAGAAGAATGGCCTGTTCGGAAACCTGATTGCCCTGAGCCTGGGCCTGACCCCCAACTTCAAGAGCAACTTCGACCTGGCC
GAGGATGCCAAACTGCAGCTGAGCAAGGACACCTACGACGACGACCTGGACAACCTGCTGGCCCAGATCGGCGACCAGTACGCCGACCT
GTTTCTGGCCGCCAAGAACCTGTCCGACGCCATCCTGCTGAGCGACATCCTGAGAGTGAACACCGAGATCACCAAGGCCCCCCTGAGCG
CCTCTATGATCAAGAGATACGACGAGCACCACCAGGACCTGACCCTGCTGAAAGCTCTCGTGCGGCAGCAGCTGCCTGAGAAGTACAAA
GAGATTTTCTTCGACCAGAGCAAGAACGGCTACGCCGGCTACATTGACGGCGGAGCCAGCCAGGAAGAGTTCTACAAGTTCATCAAGCC
CATCCTGGAAAAGATGGACGGCACCGAGGAACTGCTCGTGAAGCTGAACAGAGAGGACCTGCTGCGGAAGCAGCGGACCTTCGACAACG
GCAGCATCCCCCACCAGATCCACCTGGGAGAGCTGCACGCCATTCTGCGGCGGCAGGAAGATTTTTACCCATTCCTGAAGGACAACCGG
GAAAAGATCGAGAAGATCCTGACCTTCCGCATCCCCTACTACGTGGGCCCTCTGGCCAGGGGAAACAGCAGATTCGCCTGGATGACCAG
AAAGAGCGAGGAAACCATCACCCCCTGGAACTTCGAGGAAGTGGTGGACAAGGGCGCTTCCGCCCAGAGCTTCATCGAGCGGATGACCA
ACTTCGATAAGAACCTGCCCAACGAGAAGGTGCTGCCCAAGCACAGCCTGCTGTACGAGTACTTCACCGTGTATAACGAGCTGACCAAA
GTGAAATACGTGACCGAGGGAATGAGAAAGCCCGCCTTCCTGAGCGGCGAGCAGAAAAAGGCCATCGTGGACCTGCTGTTCAAGACCAA
CCGGAAAGTGACCGTGAAGCAGCTGAAAGAGGACTACTTCAAGAAAATCGAGTGCTTCGACTCCGTGGAAATCTCCGGCGTGGAAGATC
GGTTCAACGCCTCCCTGGGCACATACCACGATCTGCTGAAAATTATCAAGGACAAGGACTTCCTGGACAATGAGGAAAACGAGGACATT
CTGGAAGATATCGTGCTGACCCTGACACTGTTTGAGGACAGAGAGATGATCGAGGAACGGCTGAAAACCTATGCCCACCTGTTCGACGA
CAAAGTGATGAAGCAGCTGAAGCGGCGGAGATACACCGGCTGGGGCAGGCTGAGCCGGAAGCTGATCAACGGCATCCGGGACAAGCAGT
CCGGCAAGACAATCCTGGATTTCCTGAAGTCCGACGGCTTCGCCAACAGAAACTTCATGCAGCTGATCCACGACGACAGCCTGACCTTT
AAAGAGGACATCCAGAAAGCCCAGGTGTCCGGCCAGGGCGATAGCCTGCACGAGCACATTGCCAATCTGGCCGGCAGCCCCGCCATTAA
GAAGGGCATCCTGCAGACAGTGAAGGTGGTGGACGAGCTCGTGAAAGTGATGGGCCGGCACAAGCCCGAGAACATCGTGATCGAAATGG
CCAGAGAGAACCAGACCACCCAGAAGGGACAGAAGAACAGCCGCGAGAGAATGAAGCGGATCGAAGAGGGCATCAAAGAGCTGGGCAGC
CAGATCCTGAAAGAACACCCCGTGGAAAACACCCAGCTGCAGAACGAGAAGCTGTACCTGTACTACCTGCAGAATGGGCGGGATATGTA
CGTGGACCAGGAACTGGACATCAACCGGCTGTCCGACTACGATGTGGACCATATCGTGCCTCAGAGCTTTCTGAAGGACGACTCCATCG
ACAACAAGGTGCTGACCAGAAGCGACAAGAACCGGGGCAAGAGCGACAACGTGCCCTCCGAAGAGGTCGTGAAGAAGATGAAGAACTAC
TGGCGGCAGCTGCTGAACGCCAAGCTGATTACCCAGAGAAAGTTCGACAATCTGACCAAGGCCGAGAGAGGCGGCCTGAGCGAACTGGA
TAAGGCCGGCTTCATCAAGAGACAGCTGGTGGAAACCCGGCAGATCACAAAGCACGTGGCACAGATCCTGGACTCCCGGATGAACACTA
AGTACGACGAGAATGACAAGCTGATCCGGGAAGTGAAAGTGATCACCCTGAAGTCCAAGCTGGTGTCCGATTTCCGGAAGGATTTCCAG
TTTTACAAAGTGCGCGAGATCAACAACTACCACCACGCCCACGACGCCTACCTGAACGCCGTCGTGGGAACCGCCCTGATCAAAAAGTA

CCCTAAGCTGGAAAGCGAGTTCGTGTACGGCGACTACAAGGTGTACGACGTGCGGAAGATGATCGCCAAGAGCGAGCAGGAAATCGGCA
AGGCTACCGCCAAGTACTTCTTCTACAGCAACATCATGAACTTTTTCAAGACCGAGATTACCCTGGCCAACGGCGAGATCCGGAAGCGG
CCTCTGATCGAGACAAACGGCGAAACCGGGGAGATCGTGTGGGATAAGGGCCGGGATTTTGCCACCGTGCGGAAAGTGCTGAGCATGCC
CCAAGTGAATATCGTGAAAAAGACCGAGGTGCAGACAGGCGGCTTCAGCAAAGAGTCTATCCTGCCCAAGAGGAACAGCGATAAGCTGA
TCGCCAGAAAGAAGGACTGGGACCCTAAGAAGTACGGCGGCTTCGACAGCCCCACCGTGGCCTATTCTGTGCTGGTGGTGGCCAAAGTG
GAAAAGGGCAAGTCCAAGAAACTGAAGAGTGTGAAAGAGCTGCTGGGGATCACCATCATGGAAAGAAGCAGCTTCGAGAAGAATCCCAT
CGACTTTCTGGAAGCCAAGGGCTACAAAGAAGTGAAAAAGGACCTGATCATCAAGCTGCCTAAGTACTCCCTGTTCGAGCTGGAAAACG
GCCGGAAGAGAATGCTGGCCTCTGCCGGCGAACTGCAGAAGGGAAACGAACTGGCCCTGCCCTCCAAATATGTGAACTTCCTGTACCTG
GCCAGCCACTATGAGAAGCTGAAGGGCTCCCCCGAGGATAATGAGCAGAAACAGCTGTTTGTGGAACAGCACAAGCACTACCTGGACGA
GATCATCGAGCAGATCAGCGAGTTCTCCAAGAGAGTGATCCTGGCCGACGCTAATCTGGACAAAGTGCTGTCCGCCTACAACAAGCACC
GGGATAAGCCCATCAGAGAGCAGGCCaAGAATATCATCCACCTGTTTACCCTGACCAATCTGGGAGCCCCTGCCGCCTTCAAGTACTTT
GACACCACCATCGACCGGAAGAGGTACACCAGCACCAAAGAGGTGCTGGACGCCACCCTGATCCACCAGAGCATCACCGGCCTGTACGA
GACACGGATCGACCTGTCTCAGCTGGGAGGCGACAGCCCCAAGAAGAAGAGAAAGGTGGAGGCCAGCTAATAGGACCCAGCTTTCTTGT
ACAAAGTGGTGACGTAAGCTAGCAGGATCTTTGTGAAGGAACCTTACTTCTGTGGTGTGACATAATTGGACAAACTACCTACAGAGATT
TAAAGCTCTAAGGTAAATATAAAATTTTTAAGTGTATAATGTGTTAAACTACTGATTCTAATTGTTTGTGTATTTTAGATTCCAACCTA
TGGAACTGATGAATGGGAGCAGTGGTGGAATGCCTTTAATGAGGAAAACCTGTTTTGCTCAGAAGAAATGCCATCTAGTGATGATGAGG
CTACTGCTGACTCTCAACATTCTACTCCTCCAAAAAAGAAGAGAAAGGTAGttGACCCCAAGGACTTTCCTTCAGAATTGCTAAGTTTT
TTGAGTCATGCTGTGTTTAGTAATAGAACTCTTGCTTGCTTTGCTATTTACACCACAAAGGAAAAAGCTGCACTGCTATACAAGAAAAT
TATGGAAAAATATTCTGTAACCTTTATAAGTAGGCATAACAGTTATAATCATAACATACTGTTTTTTCTTACTCCACACAGGCATAGAG
TGTCTGCTATTAATAACTATGCTCAAAAATTGTGTACCTTTAGCTTTTTAATTTGTAAAGGGGTTAATAAGGAATATTTGATGTATAGT
GCCTTGACTAGAGATCATAATCAGCCATACCACATTTGTAGAGGTTTTACTTGCTTTAAAAAACCTCCCACACCTCCCCCTGAACCTGA
AACATAAAATGAATGCAATTGTTGTTGTTAACTTGTTTATTGCAGCTTATAATGGTTACAAATAAAGCAATAGCATCACAAATTTCACA
AATAAAGCATTTTTTTCACTGCATTCTAGTTGTGGTTTGTCCAAACTCATCAATGTATCTTATCATGTCTGGATCCCGTTTAAACTACG
CGTAATTCAAACAGGGTTCTGGCGTCGTTCTCGTACTGTTTTCCCCAGGCCAGTGCTTTAGCGTTATTGAAAAAGGAAGAGTATGAGTA
TTCAACATTTCCGTGTCGCCCTTATTCCCTTTTTTGCGGCATTTTGCCTTCCTGTTTTTGCTCACCCAGAAACGCTGGTGAAAGTAAAA
GATGCTGAAGATCAGTTGGGTGCACGAGTGGGTTACATCGAACTGGATCTCAACAGCGGTAAGATCCTTGAGAGTTTTCGCCCCGAAGA
ACGTTTTCCAATGATGAGCACTTTTAAAGTTCTGCTATGTGGCGCGGTATTATCCCGTATTGACGCCGGGCAAGAGCAACTCGGTCGCC
GCATACACTATTCTCAGAATGACTTGGTTGAGTACTCACCAGTCACAGAAAAGCATCTTACGGATGGCATGACAGTAAGAGAATTATGC
AGTGCTGCCATAACCATGAGTGATAACACTGCGGCCAACTTACTTCTGACAACGATCGGAGGACCGAAGGAGCTAACCGCTTTTTTGCA
CAACATGGGGGATCATGTAACTCGCCTTGATCGTTGGGAACCGGAGCTGAATGAAGCCATACCAAACGACGAGCGTGACACCACGATGC
CTGTAGCAATGGCAACAACGTTGCGCAAACTATTAACTGGCGAACTACTTACTCTAGCTTCCCGGCAACAATTAATAGACTGGATGGAG
GCGGATAAAGTTGCAGGACCACTTCTGCGCTCGGCCCTTCCGGCTGGCTGGTTTATTGCTGATAAATCTGGAGCCGGTGAGCGTGGGTC
TCGCGGTATCATTGCAGCACTGGGGCCAGATGGTAAGCCCTCCCGTATCGTAGTTATCTACACGACGGGGAGTCAGGCAACTATGGATG
AACGAAATAGACAGATCGCTGAGATAGGTGCCTCACTGATTAAGCATTGGTAACTGTCAGACCAAGTTTACTCATATATACTTTAGATT
GATTTAAAACTTCATTTTTAATTTAAAAGGATCTAGGTGAAGATCCTTTTTGATAATCTCATGACCAAAATCCCTTAACGTGAGTTTTC
GTTCCACTGAGCGTCAGACCCCGTAGAAAAGATCAAAGGATCTTCTTGAGATCCTTTTTTTCTGCGCGTAATCTGCTGCTTGCAAACAA
AAAAACCACCGCTACCAGCGGTGGTTTGTTTGCCGGATCAAGAGCTACCAACTCTTTTTCCGAAGGTAACTGGCTTCAGCAGAGCGCAG
ATACCAAATACTGTTCTTCTAGTGTAGCCGTAGTTAGGCCACCACTTCAAGAACTCTGTAGCACCGCCTACATACCTCGCTCTGCTAAT
CCTGTTACCAGTGGCTGCTGCCAGTGGCGATAAGTCGTGTCTTACCGGGTTGGACTCAAGACGATAGTTACCGGATAAGGCGCAGCGGT
CGGGCTGAACGGGGGGTTCGTGCACACAGCCCAGCTTGGAGCGAACGACCTACACCGAACTGAGATACCTACAGCGTGAGCTATGAGAA
AGCGCCACGCTTCCCGAAGGGAGAAAGGCGGACAGGTATCCGGTAAGCGGCAGGGTCGGAACAGGAGAGCGCACGAGGGAGCTTCCAGG
GGGAAACGCCTGGTATCTTTATAGTCCTGTCGGGTTTCGCCACCTCTGACTTGAGCGTCGATTTTTGTGATGCTCGTCAGGGGGGCGGA
GCCTATGGAAAAACGCCAGCAACGCGGCCTTTTTACGGTTCCTGGCCTTTTGCTGGCCTTTTGCTCACATGTTCTTTCCTGCGTTATCC
CCTGATTCTGTGGATAACCGTATTACCGCCTTTGAGTGAGCTGATACCGCTCGCCGCAGCCGAACGACCGAGCGCAGCGAGTCAGTGAG
CGAGGAAGCGGAAGA

pKRG3-mU6-PUb-hSpCas9-pAc

aae U6
BsmBI sites for sgRNA cloning
sgRNA tracrRNA and U6 terminator
aae PUb promoter
NLS
hSpCas9
T2A
pAc

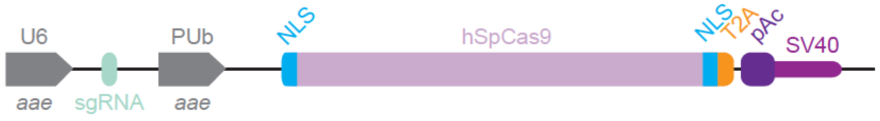

GCGCCCAATACGCAAACCGCCTCTCCCCGCGGGTTGGCCGATTCATTAATGCAGGCAACTCGTGAAAGGTAGGCGGATCAGCGAATGAA
ATCGCCCATCGAGTTGATACGTCCATCCATCGCTAGAACCGCGTTCGCTGTAGAAGACTATATAAGAGCAGAGGCAAGAGTAGTGAAAT
gGAGACGaCGTCTCtGTTTTAGAGCTAGAAATAGCAAGTTAAAATAAGGCTAGTCCGTTATCAACTTGAAAAAGTGGCACCGAGTCGGT
GCTTTTTTGTTTTAGAGCTAGAAATAGCAAGTTAAAATAAGGCTAGTCCGTTTTTAGCGCTTGCGCCAATTCTGCAGACAAATGGCTAT
CTTTACATGTAGCTTGTGCATTGAATCCAATTATAATTTGCCTTGGCACCAGCTGAGCCAGACAAGAAAGAAAGCTTCCCAGAAGTATA
TCGATTTAGAAGGGTTGACGTCACTTGCTGACTGCACTAATACAGCAAATGATGCAATTAGAATGATTCAAGTGAAATTCCCAAATTAC
TGATTTTTCTCTGGATTTGGTTATCAGATTACATTCGAAGCTAAGATTAGCTACCGAAATTGTCGATCAAATCAGGAAATCCTTTCTCT
ATCGAAAAAGGCATTCGCACATCTTCCTCTCTATGCCATATACACGAAGGGTAGGTACATTGACGTCTTTGCCAGAAGTTGAACTGCAT
CGTTCAAGGTACAGAATGAACGACTAACAGACACAAGCACGTTTTGCTGTCCATTCAGACACAGGGATGGTACCCATATTCGATCGATA
TAGAGCCATCCAACCGAACAGAGGTATATGTATGAATGTATTGCTGAAATTTTCTAGAAGTACAACCACCACTACGACAGTGTCTATAA
AACGCCCCTGCAAAGGCGAAACCAGCTCAATCGAATACGTTTCCTAGTGGAGTGAACATTACGCGGCCCAAGTAAGCAGTGCCAGTGCA
AGTGAAGTGAAGTCTCTAGTGAAAAAGAGTGATCCAATTAGCCAGAGGAGAAAATTTCAGAGTGAACAAAGCTTTATTCAAAGGACAAT
TACTATTAAATTGGTGAAAGTGCATTTCGGTGAAGGGAATCTTCTAGTGAAGGTAGGTAAATTAATTGATGAAATTATAGCTATGAGCG
AAAACTAGTTTGGTGAATGATTCCTTTGTCTTTGAATGAGCAAACTATTTTCCAAGATGGCGACTATTGAGCTTTGAGTGATTAGTGAA
AATTTGCAACGCAGTTTCATCATCATTGATAAAACCCAATTGTGATTCACAGCGATAATCATATTTCGTTGAATCATCGCTACTAATTG
AATTAAATTTCTAGAATAATAAGAATAACGTATTTGCTCCGTCACATATCTAAAATAAATATTTTGATGGTAATTACCCATTAAGGTAA
TATTAACACATATCGAGAAAAACCTTGAGGAAATCGTGAAAACTTGAAGATACGCAATTTCAAAACTACGTAGTTCAAAGTCGAAAACA
AGTTAATTTTTCACTTAAAAGTAGGGCGTTGTTGTGACGTCATCACCTTCAAGTGTATATTTTTCACTTGGCCTGCGACTGCAAACGCA
GACAAAGCAAAACAAGTTTAAAACCTGTCGTGTCGTGCTCGAAGCCAAAGGCAATGAATCAATATCAAATGAGAGTTTGCATTTCACAA
CCAATTACTCAAGCGTTTCCTCGTTTCTTTTTCTGCTCAACAGAGATTTCAACACCAAGCTCGATAAGCTTGTTCGAATCTCGAGTGCG
CGCTTCCGGAGGTATACACCTAGGCGGTACCACTGCAGTGAATTCGGAGCTCTACCGGTGCCACCATGGCCCCAAAGAAGAAGCGGAAG
GTCGGTATCCACGGAGTCCCAGCAGCCGACAAGAAGTACAGCATCGGCCTGGACATCGGCACCAACTCTGTGGGCTGGGCCGTGATCAC
CGACGAGTACAAGGTGCCCAGCAAGAAATTCAAGGTGCTGGGCAACACCGACCGGCACAGCATCAAGAAGAACCTGATCGGAGCCCTGC
TGTTCGACAGCGGCGAAACAGCCGAGGCCACCCGGCTGAAGAGAACCGCCAGAAGAAGATACACCAGACGGAAGAACCGGATCTGCTAT
CTGCAAGAGATCTTCAGCAACGAGATGGCCAAGGTGGACGACAGCTTCTTCCACAGACTGGAAGAGTCCTTCCTGGTGGAAGAGGATAA
GAAGCACGAGCGGCACCCCATCTTCGGCAACATCGTGGACGAGGTGGCCTACCACGAGAAGTACCCCACCATCTACCACCTGAGAAAGA
AACTGGTGGACAGCACCGACAAGGCCGACCTGCGGCTGATCTATCTGGCCCTGGCCCACATGATCAAGTTCCGGGGCCACTTCCTGATC
GAGGGCGACCTGAACCCCGACAACAGCGACGTGGACAAGCTGTTCATCCAGCTGGTGCAGACCTACAACCAGCTGTTCGAGGAAAACCC
CATCAACGCCAGCGGCGTGGACGCCAAGGCCATCCTGTCTGCCAGACTGAGCAAGAGCAGACGGCTGGAAAATCTGATCGCCCAGCTGC
CCGGCGAGAAGAAGAATGGCCTGTTCGGAAACCTGATTGCCCTGAGCCTGGGCCTGACCCCCAACTTCAAGAGCAACTTCGACCTGGCC
GAGGATGCCAAACTGCAGCTGAGCAAGGACACCTACGACGACGACCTGGACAACCTGCTGGCCCAGATCGGCGACCAGTACGCCGACCT
GTTTCTGGCCGCCAAGAACCTGTCCGACGCCATCCTGCTGAGCGACATCCTGAGAGTGAACACCGAGATCACCAAGGCCCCCCTGAGCG
CCTCTATGATCAAGAGATACGACGAGCACCACCAGGACCTGACCCTGCTGAAAGCTCTCGTGCGGCAGCAGCTGCCTGAGAAGTACAAA
GAGATTTTCTTCGACCAGAGCAAGAACGGCTACGCCGGCTACATTGACGGCGGAGCCAGCCAGGAAGAGTTCTACAAGTTCATCAAGCC
CATCCTGGAAAAGATGGACGGCACCGAGGAACTGCTCGTGAAGCTGAACAGAGAGGACCTGCTGCGGAAGCAGCGGACCTTCGACAACG
GCAGCATCCCCCACCAGATCCACCTGGGAGAGCTGCACGCCATTCTGCGGCGGCAGGAAGATTTTTACCCATTCCTGAAGGACAACCGG
GAAAAGATCGAGAAGATCCTGACCTTCCGCATCCCCTACTACGTGGGCCCTCTGGCCAGGGGAAACAGCAGATTCGCCTGGATGACCAG
AAAGAGCGAGGAAACCATCACCCCCTGGAACTTCGAGGAAGTGGTGGACAAGGGCGCTTCCGCCCAGAGCTTCATCGAGCGGATGACCA
ACTTCGATAAGAACCTGCCCAACGAGAAGGTGCTGCCCAAGCACAGCCTGCTGTACGAGTACTTCACCGTGTATAACGAGCTGACCAAA
GTGAAATACGTGACCGAGGGAATGAGAAAGCCCGCCTTCCTGAGCGGCGAGCAGAAAAAGGCCATCGTGGACCTGCTGTTCAAGACCAA
CCGGAAAGTGACCGTGAAGCAGCTGAAAGAGGACTACTTCAAGAAAATCGAGTGCTTCGACTCCGTGGAAATCTCCGGCGTGGAAGATC
GGTTCAACGCCTCCCTGGGCACATACCACGATCTGCTGAAAATTATCAAGGACAAGGACTTCCTGGACAATGAGGAAAACGAGGACATT
CTGGAAGATATCGTGCTGACCCTGACACTGTTTGAGGACAGAGAGATGATCGAGGAACGGCTGAAAACCTATGCCCACCTGTTCGACGA
CAAAGTGATGAAGCAGCTGAAGCGGCGGAGATACACCGGCTGGGGCAGGCTGAGCCGGAAGCTGATCAACGGCATCCGGGACAAGCAGT
CCGGCAAGACAATCCTGGATTTCCTGAAGTCCGACGGCTTCGCCAACAGAAACTTCATGCAGCTGATCCACGACGACAGCCTGACCTTT
AAAGAGGACATCCAGAAAGCCCAGGTGTCCGGCCAGGGCGATAGCCTGCACGAGCACATTGCCAATCTGGCCGGCAGCCCCGCCATTAA
GAAGGGCATCCTGCAGACAGTGAAGGTGGTGGACGAGCTCGTGAAAGTGATGGGCCGGCACAAGCCCGAGAACATCGTGATCGAAATGG
CCAGAGAGAACCAGACCACCCAGAAGGGACAGAAGAACAGCCGCGAGAGAATGAAGCGGATCGAAGAGGGCATCAAAGAGCTGGGCAGC
CAGATCCTGAAAGAACACCCCGTGGAAAACACCCAGCTGCAGAACGAGAAGCTGTACCTGTACTACCTGCAGAATGGGCGGGATATGTA
CGTGGACCAGGAACTGGACATCAACCGGCTGTCCGACTACGATGTGGACCATATCGTGCCTCAGAGCTTTCTGAAGGACGACTCCATCG
ACAACAAGGTGCTGACCAGAAGCGACAAGAACCGGGGCAAGAGCGACAACGTGCCCTCCGAAGAGGTCGTGAAGAAGATGAAGAACTAC
TGGCGGCAGCTGCTGAACGCCAAGCTGATTACCCAGAGAAAGTTCGACAATCTGACCAAGGCCGAGAGAGGCGGCCTGAGCGAACTGGA
TAAGGCCGGCTTCATCAAGAGACAGCTGGTGGAAACCCGGCAGATCACAAAGCACGTGGCACAGATCCTGGACTCCCGGATGAACACTA

AGTACGACGAGAATGACAAGCTGATCCGGGAAGTGAAAGTGATCACCCTGAAGTCCAAGCTGGTGTCCGATTTCCGGAAGGATTTCCAG
TTTTACAAAGTGCGCGAGATCAACAACTACCACCACGCCCACGACGCCTACCTGAACGCCGTCGTGGGAACCGCCCTGATCAAAAAGTA
CCCTAAGCTGGAAAGCGAGTTCGTGTACGGCGACTACAAGGTGTACGACGTGCGGAAGATGATCGCCAAGAGCGAGCAGGAAATCGGCA
AGGCTACCGCCAAGTACTTCTTCTACAGCAACATCATGAACTTTTTCAAGACCGAGATTACCCTGGCCAACGGCGAGATCCGGAAGCGG
CCTCTGATCGAGACAAACGGCGAAACCGGGGAGATCGTGTGGGATAAGGGCCGGGATTTTGCCACCGTGCGGAAAGTGCTGAGCATGCC
CCAAGTGAATATCGTGAAAAAGACCGAGGTGCAGACAGGCGGCTTCAGCAAAGAGTCTATCCTGCCCAAGAGGAACAGCGATAAGCTGA
TCGCCAGAAAGAAGGACTGGGACCCTAAGAAGTACGGCGGCTTCGACAGCCCCACCGTGGCCTATTCTGTGCTGGTGGTGGCCAAAGTG
GAAAAGGGCAAGTCCAAGAAACTGAAGAGTGTGAAAGAGCTGCTGGGGATCACCATCATGGAAAGAAGCAGCTTCGAGAAGAATCCCAT
CGACTTTCTGGAAGCCAAGGGCTACAAAGAAGTGAAAAAGGACCTGATCATCAAGCTGCCTAAGTACTCCCTGTTCGAGCTGGAAAACG
GCCGGAAGAGAATGCTGGCCTCTGCCGGCGAACTGCAGAAGGGAAACGAACTGGCCCTGCCCTCCAAATATGTGAACTTCCTGTACCTG
GCCAGCCACTATGAGAAGCTGAAGGGCTCCCCCGAGGATAATGAGCAGAAACAGCTGTTTGTGGAACAGCACAAGCACTACCTGGACGA
GATCATCGAGCAGATCAGCGAGTTCTCCAAGAGAGTGATCCTGGCCGACGCTAATCTGGACAAAGTGCTGTCCGCCTACAACAAGCACC
GGGATAAGCCCATCAGAGAGCAGGCCaAGAATATCATCCACCTGTTTACCCTGACCAATCTGGGAGCCCCTGCCGCCTTCAAGTACTTT
GACACCACCATCGACCGGAAGAGGTACACCAGCACCAAAGAGGTGCTGGACGCCACCCTGATCCACCAGAGCATCACCGGCCTGTACGA
GACACGGATCGACCTGTCTCAGCTGGGAGGCGACAAAAGGCCGGCGGCCACGAAAAAGGCCGGCCAGGCAAAAAAGAAAAAGGGATCAG
GCAAGCTTGAGGGCAGAGGAAGTCTTCTAACATGCGGTGACGTGGAGGAGAATCCCGGCCCTGCTAGCGGTAGCGGCAGCGGTAGCATG
ACCGAGTACAAGCCCACGGTGCGCCTCGCCACCCGCGACGACGTCCCCCGGGCCGTACGCACCCTCGCCGCCGCGTTCGCCGACTACCC
CGCCACGCGCCACACCGTCGACCCGGACCGCCACATCGAGCGGGTCACCGAGCTGCAAGAACTCTTCCTCACGCGCGTCGGGCTCGACA
TCGGCAAGGTGTGGGTCGCGGACGACGGCGCCGCGGTGGCGGTCTGGACCACGCCGGAGAGCGTCGAAGCGGGGGCGGTGTTCGCCGAG
ATCGGCCCGCGCATGGCCGAGTTGAGCGGTTCCCGGCTGGCCGCGCAGCAACAGATGGAAGGCCTCCTGGCGCCGCACCGGCCCAAGGA
GCCCGCGTGGTTCCTGGCCACCGTCGGCGTCTCGCCCGACCACCAGGGCAAGGGTCTGGGCAGCGCCGTCGTGCTCCCCGGAGTGGAGG
CGGCCGAGCGCGCCGGGGTGCCCGCCTTCCTGGAGACCTCCGCGCCCCGCAACCTCCCCTTCTACGAGCGGCTCGGCTTCACCGTCACC
GCCGACGTCGAGGTGCCCGAAGGACCGCGCACCTGGTGCATGACCCGCAAGCCCGGTGCCTAATAGGACCCAGCTTTCTTGTACAAAGT
GGTGACGTAAGCTAGCAGGATCTTTGTGAAGGAACCTTACTTCTGTGGTGTGACATAATTGGACAAACTACCTACAGAGATTTAAAGCT
CTAAGGTAAATATAAAATTTTTAAGTGTATAATGTGTTAAACTACTGATTCTAATTGTTTGTGTATTTTAGATTCCAACCTATGGAACT
GATGAATGGGAGCAGTGGTGGAATGCCTTTAATGAGGAAAACCTGTTTTGCTCAGAAGAAATGCCATCTAGTGATGATGAGGCTACTGC
TGACTCTCAACATTCTACTCCTCCAAAAAAGAAGAGAAAGGTAGttGACCCCAAGGACTTTCCTTCAGAATTGCTAAGTTTTTTGAGTC
ATGCTGTGTTTAGTAATAGAACTCTTGCTTGCTTTGCTATTTACACCACAAAGGAAAAAGCTGCACTGCTATACAAGAAAATTATGGAA
AAATATTCTGTAACCTTTATAAGTAGGCATAACAGTTATAATCATAACATACTGTTTTTTCTTACTCCACACAGGCATAGAGTGTCTGC
TATTAATAACTATGCTCAAAAATTGTGTACCTTTAGCTTTTTAATTTGTAAAGGGGTTAATAAGGAATATTTGATGTATAGTGCCTTGA
CTAGAGATCATAATCAGCCATACCACATTTGTAGAGGTTTTACTTGCTTTAAAAAACCTCCCACACCTCCCCCTGAACCTGAAACATAA
AATGAATGCAATTGTTGTTGTTAACTTGTTTATTGCAGCTTATAATGGTTACAAATAAAGCAATAGCATCACAAATTTCACAAATAAAG
CATTTTTTTCACTGCATTCTAGTTGTGGTTTGTCCAAACTCATCAATGTATCTTATCATGTCTGGATCCCGTTTAAACTACGCGTAATT
CAAACAGGGTTCTGGCGTCGTTCTCGTACTGTTTTCCCCAGGCCAGTGCTTTAGCGTTATTGAAAAAGGAAGAGTATGAGTATTCAACA
TTTCCGTGTCGCCCTTATTCCCTTTTTTGCGGCATTTTGCCTTCCTGTTTTTGCTCACCCAGAAACGCTGGTGAAAGTAAAAGATGCTG
AAGATCAGTTGGGTGCACGAGTGGGTTACATCGAACTGGATCTCAACAGCGGTAAGATCCTTGAGAGTTTTCGCCCCGAAGAACGTTTT
CCAATGATGAGCACTTTTAAAGTTCTGCTATGTGGCGCGGTATTATCCCGTATTGACGCCGGGCAAGAGCAACTCGGTCGCCGCATACA
CTATTCTCAGAATGACTTGGTTGAGTACTCACCAGTCACAGAAAAGCATCTTACGGATGGCATGACAGTAAGAGAATTATGCAGTGCTG
CCATAACCATGAGTGATAACACTGCGGCCAACTTACTTCTGACAACGATCGGAGGACCGAAGGAGCTAACCGCTTTTTTGCACAACATG
GGGGATCATGTAACTCGCCTTGATCGTTGGGAACCGGAGCTGAATGAAGCCATACCAAACGACGAGCGTGACACCACGATGCCTGTAGC
AATGGCAACAACGTTGCGCAAACTATTAACTGGCGAACTACTTACTCTAGCTTCCCGGCAACAATTAATAGACTGGATGGAGGCGGATA
AAGTTGCAGGACCACTTCTGCGCTCGGCCCTTCCGGCTGGCTGGTTTATTGCTGATAAATCTGGAGCCGGTGAGCGTGGGTCTCGCGGT
ATCATTGCAGCACTGGGGCCAGATGGTAAGCCCTCCCGTATCGTAGTTATCTACACGACGGGGAGTCAGGCAACTATGGATGAACGAAA
TAGACAGATCGCTGAGATAGGTGCCTCACTGATTAAGCATTGGTAACTGTCAGACCAAGTTTACTCATATATACTTTAGATTGATTTAA
AACTTCATTTTTAATTTAAAAGGATCTAGGTGAAGATCCTTTTTGATAATCTCATGACCAAAATCCCTTAACGTGAGTTTTCGTTCCAC
TGAGCGTCAGACCCCGTAGAAAAGATCAAAGGATCTTCTTGAGATCCTTTTTTTCTGCGCGTAATCTGCTGCTTGCAAACAAAAAAACC
ACCGCTACCAGCGGTGGTTTGTTTGCCGGATCAAGAGCTACCAACTCTTTTTCCGAAGGTAACTGGCTTCAGCAGAGCGCAGATACCAA
ATACTGTTCTTCTAGTGTAGCCGTAGTTAGGCCACCACTTCAAGAACTCTGTAGCACCGCCTACATACCTCGCTCTGCTAATCCTGTTA
CCAGTGGCTGCTGCCAGTGGCGATAAGTCGTGTCTTACCGGGTTGGACTCAAGACGATAGTTACCGGATAAGGCGCAGCGGTCGGGCTG
AACGGGGGGTTCGTGCACACAGCCCAGCTTGGAGCGAACGACCTACACCGAACTGAGATACCTACAGCGTGAGCTATGAGAAAGCGCCA
CGCTTCCCGAAGGGAGAAAGGCGGACAGGTATCCGGTAAGCGGCAGGGTCGGAACAGGAGAGCGCACGAGGGAGCTTCCAGGGGGAAAC
GCCTGGTATCTTTATAGTCCTGTCGGGTTTCGCCACCTCTGACTTGAGCGTCGATTTTTGTGATGCTCGTCAGGGGGGCGGAGCCTATG
GAAAAACGCCAGCAACGCGGCCTTTTTACGGTTCCTGGCCTTTTGCTGGCCTTTTGCTCACATGTTCTTTCCTGCGTTATCCCCTGATT
CTGTGGATAACCGTATTACCGCCTTTGAGTGAGCTGATACCGCTCGCCGCAGCCGAACGACCGAGCGCAGCGAGTCAGTGAGCGAGGAA
GCGGAAGA

pSL1180-HR-Aag2-loxP-PUb-RFP-loxP-3XFLAG-AGO1

AGO1 homology with silent mutations at sgRNA cleavage sites

loxP sites

aae PUb promoter

RFP

3xFLAG

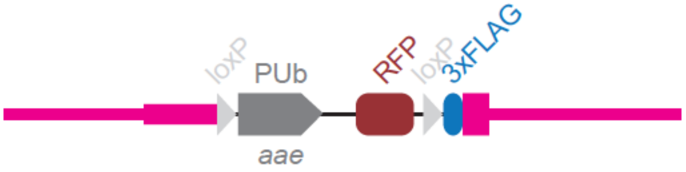

GCGCCCAATACGCAAACCGCCTCTCCCCGCGCGTTGGCCGATTCATTAATGCAGCTGGCACGACAGGTTTCCCGACTGGAAAGCGGGCA
GTGAGCGCAACGCAATTAATGTGAGTTAGCTCACTCATTAGGCACCCCAGGCTTTACACTTTATGCTTCCGGCTCGTATGTTGTGTGGA
ATTGTGAGCGGATAACAATTTCACACAGGAAACAGCTATGACCATGATTACGAATTCGACATGTAGGACATGTGGGGTAGTTCCAGAAA
TCCAGATAAACGATGGACATTTGAATTATCCCCAAACCTCCATCATGTTCTGTTACTTGATATCAACTCAAGGAACCCCGCACTAAAAT
TGAATTTCATGGCTACTATGATAACCGTGTTAAACCGTTTTCTAAAAGTTTCTGTCGCATGACTCGTTACAAATGACGTTACAAATCTA
TAATTTCTATAAATCCCTTCAAAATTGACTCTTGGAAATTTATCTGTATTAAAATTATATAATTATTTTAAAAGCTGGTAAATCACCAA
CATTTTGACATCCATTTCATCCAGAATCGTTCTATTGTTTATGCGGTGAATGCCGAAAAAAATATACCTTCAATGATTCATTGCCGATG
TTTCAACTGAACTCAAACTCAGAAATATGAAATTTCACGCATTTAGATAGATGACTACTCTTTTTTCAGAGTTTTAGTTCACAGCAAAG
ATAAAAGGTAATTCAAATGAGAGCTGCTGTAGAAAGTTTCTCGCTCAATGTATGATCTCGCCTTAAAGCCATTGGTAACCAAGTGTGTA
GCTTGTTGTTACTACGCAAACGAATGGATTTACACGTTACTTAAAAATGCTACAATGAAGATTCTCTATCTCCCAGTGGCGATAGTTAT
ATTTTGGTCCATTCATTTGCTTAAAAACAACGAGCTACACATCAATGGCTTTTAGATCGAGATCACAATTTATGCGAGATTTCCTCAAG
TAATTTATTGCCAAGAAATTTCGTGAATAATAATAATTATAATGATTATTCTGTTTCATGAGCGCATCCATTTTGGAGCGTCCGTAAAA
TGAAACAAAACGGTAAACCGCAACCTGGCTGTTTACTTGCAGCGGTTTTTCGGTACCTAAACTTGCGTACAAGTCGAACTCGTCTAAAA
CATAACGCCTACCCAGGCTGCTGGTAGCGAAACGTTGGGGGGCGATGAAGAAAACTTGTCTCACTCGCCATCATACGAGAGTTTCGCGA
CGAGTTCAGCAGACGTGCGTGTTTAGCGCGACGACGAAAAGTGCAAAAATTCGGCCGTAATTAGTTCGCTTCCGTTTTTCCGAGTGCCT
CCGATCGTTAGCGGACCGTTCCGGGTGTGATTTAAGCGTGAAAATAACTTCGTATAATGTATGCTATACGAAGTTATTATCTTTACATG
TAGCTTGTGCATTGAATCCAATTATAATTTGCCTTGGCACCAGCTGAGCCAGACAAGAAAGAAGCTTCCCAGAAGTATATCGATTTAG
AAGGGTTGACGTCACTTGCTGACTGCACTAATACAGCAAATGATGCAATTAGAATGATTCAAGTGAAATTCCCAAATTACTGATTTTTC
TCTGGATTTGGTTATCAGATTACATTCGAAGCTAAGATTAGCTACCGAAATTGTCGATCAAATCAGGAAATCCTTTCTCTATCGAAAAA
GGCATTCGCACATCTTCCTCTCTATGCCATATACACGAAGGGTAGGTACATTGACGTCTTTGCCAGAAGTTGAACTGCATCGTTCAAGG
TACAGAATGAACGACTAACAGACACAAGCACGTTTTGCTGTCCATTCAGACACAGGGATGGTACCCATATTCGATCGATATAGAGCCAT
CCAACCGAACAGAGGTATATGTATGAATGTATTGCTGAAATTTTCTAGAAGTACAACCACCACTACGACAGTGTCTATAAAACGCCCCT
GCAAAGGCGAAACCAGCTCAATCGAATACGTTTCCTAGTGGAGTGAACATTACGCGGCCCAAGTAAGCAGTGCCAGTGCAAGTGAAGTG
AAGTCTCTAGTGAAAAAGAGTGATCCAATTAGCCAGAGGAGAAAATTTCAGAGTGAACAAAGCTTTATTCAAAGGACAATTACTATTAA
ATTGGTGAAAGTGCATTTCGGTGAAGGGAATCTTCTAGTGAAGGTAGGTAAATTAATTGATGAAATTATAGCTATGAGCGAAAACTAGT
TTGGTGAATGATTCCTTTGTCTTTGAATGAGCAAACTATTTTCCAAGATGGCGACTATTGAGCTTTGAGTGATTAGTGAAAATTTGCAA
CGCAGTTTCATCATCATTGATAAAACCCAATTGTGATTCACAGCGATAATCATATTTCGTTGAATCATCGCTACTAATTGAATTAAATT
TCTAGAATAATAAGAATAACGTATTTGCTCCGTCACATATCTAAAATAAATATTTTGATGGTAATTACCCATTAAGGTAATATTAACAC
ATATCGAGAAAAACCTTGAGGAAATCGTGAAAACTTGAAGATACGCAATTTCAAAACTACGTAGTTCAAAGTCGAAAACAAGTTAATTT
TTCACTTAAAAGTAGGGCGTTGTTGTGACGTCATCACCTTCAAGTGTATATTTTTCACTTGGCCTGCGACTGCAAACGCAGACAAAGCA
AAACAAGTTTAAAACCTGTCGTGTCGTGCTCGAAGCCAAAGGCAATGAATCAATATCAAATGAGAGTTTGCATTTCACAACCAATTACT
CAAGCGTTTCCTCGTTTCTTTTTCTGCTCAACAGAGATTTCAACACCAAGCTCGATAAGCTTGTTCGAATCTCGAGTGCGCGCTTCCGG
AGGTATACACCTAGGCGGTACCACTGCAGTGAATTCGGAGCTCTACCGCGGCCGCATGAGCGAGCTGATCAAGGAGAACATGCACATGA
AGCTGTACATGGAGGGCACCGTGAACAACCACCACTTCAAGTGCACATCCGAGGGCGAAGGCAAGCCCTACGAGGGCACCCAGACCATG
AAGATCAAGGTGGTCGAGGGCGGCCCTCTCCCCTTCGCCTTCGACATCCTGGCTACCAGCTTCATGTACGGCAGCAAAGCCTTCATCAA
CCACACCCAGGGCATCCCCGACTTCTTTAAGCAGTCCTTCCCTGAGGGCTTCACATGGGAGAGAATCACCACATACGAAGACGGGGGCG
TGCTGACCGCTACCCAGGACACCAGCTTCCAGAACGGCTGCATCATCTACAACGTCAAGATCAACGGGGTGAACTTCCCATCCAACGGC
CCTGTGATGCAGAAGAAAACACGCGGCTGGGAGGCCAACACCGAGATGCTGTACCCCGCTGACGGCGGCCTGAGAGGCCACAGCCAGAT
GGCCCTGAAGCTCGTGGGCGGGGGCTACCTGCACTGCTCCTTCAAGACCACATACAGATCCAAGAAACCCGCTAAGAACCTCAAGATGC
CCGGCTTCCACTTCGTGGACCACAGACTGGAAAGAATCAAGGAGGCCGACAAAGAGACCTACGTCGAGCAGCACGAGATGGCTGTGGCC
AAGTACTGCGACCTCCCTAGCAAACTGGGGCACAGATAATTAATTAATAACTTCGTATAATGTATGCTATACGAAGTTATATGGATTAC
AAGGATCACGATGGAGATTACAAGGATCACGATATCGATTACAAGGATGATGATAAGTATCCGGTGGGTCAACGTAAGTACCGTTTCCG
GACGTTTGAAAAAAGTGCAATTCACAAGAGAAGAAAAAAAAAaGTGTTGgCGAGGGGACCTTCCTTCCTCCCTCTCGTCTCTCTCGCTG
TGTAAATATTTCTTCCCCCATGTGTGTAATTTTCCTGGCCTGCTGCTCTGTTTGGTGCTTCTCTGGACGTTTTTGTGCGTGTGcGTTGG
TGTGATGGGGGGCTTTTCCTGTGTACGGACGAAGAACTTTGTGTCGGTGTGCGTGTGAGATGAGGCCCTCGCCAGAATGAAAATAAACC
TCATCAACAATGAAAAGTGTCACCACTTTATGGAATAAAAAACGTTTTTATGCAGAAGAGCATGTGAATTATGTATCACTTGGCGATGG
AATTGGGAACATTGATGCTTCCGGACCGATGGAATAATGGAAAACCAGATGTTTCTGCACGTTCTTTGGCACCTTGGCAATGTGTACTA
ATCAACACCTCCCTCGAAAAATATACTTGGTGATCAGAATGTTTGTGTTTGTGAATAGTAGGTAGTTTCCGTGTGTGGGTGTGTGTCTT
TGCTAATCATCCTGCTCCAGAAAAAAAGGAAAACCACCAATTTTAATCATCATTTTCTGCGGCTTCGGAGACTGATAAGAGGAATACAA
CTGTTTGCAAACAGTGGATTGAAAACAATGGATAGAAGTTTTATTGGTTCACTTCAGTTCTAAACAGCGGATCCATCTGAAAAGTTATA
TGAAATTGTACCCCCCTTTTCAGTGCTGGGAAATTATTGCCTTTTGTGTCGCTCTTTGCTCACAACTACATACGGAGATGCAGACAGAGC
TCGCATTATGTTGGTGGTGCTCTGTCGCTGGCCATCCGAGAATCGGAAACATCGTGCGGATGGAACATGGAAGAAGAGGACGTTGTAGG
GTTTACCATTATGTATATGCGTTCAGGCGACATCAGACGAGGTGGGTCTTCTCGAAATGATGGGTGGTTGACTGAGTAGCACCTGCTGT
TCATGTGCGGAAATGGAATAACGTGGAGATGCAATGTTCTTCAACGCTAGAAGGATTCGGGGAGACGAAAAGCAAATCCGGTGGCGGCC
GCCTGCAGCTGGCGCCATCGATACGCGTACGTCGCGACCGCGGACATGTACAGAGCTCGAGAAGTACTAGTGGCCACGTGGGCCGTGCA

CCTTAAGCTTGGCACTGGCCGTCGTTTTACAACGTCGTGACTGGGAAAACCCTGGCGTTACCCAACTTAATCGCCTTGCAGCACATCCC
CCTTTCGCCAGCTGGCGTAATAGCGAAGAGGCCCGCACCGATCGCCCTTCCCAACAGTTGCGCAGCCTGAATGGCGAATGGCGCCTGAT
GCGGTATTTTCTCCTTACGCATCTGTGCGGTATTTCACACCGCATACGTCAAAGCAACCATAGTACGCGCCCTGTAGCGGCGCATTAAG
CGCGGCGGGTGTGGTGGTTACGCGCAGCGTGACCGCTACACTTGCCAGCGCCCTAGCGCCCGCTCCTTTCGCTTTCTTCCCTTCCTTTC
TCGCCACGTTCGCCGGCTTTCCCCGTCAAGCTCTAAATCGGGGGCTCCCTTTAGGGTTCCGATTTAGTGCTTTACGGCACCTCGACCCC
AAAAAACTTGATTTGGGTGATGGTTCACGTAGTGGGCCATCGCCCTGATAGACGGTTTTTCGCCCTTTGACGTTGGAGTCCACGTTCTT
TAATAGTGGACTCTTGTTCCAAACTGGAACAACACTCAACCCTATCTCGGGCTATTCTTTTGATTTATAAGGGATTTTGCCGATTTCGG
CCTATTGGTTAAAAAATGAGCTGATTTAACAAAAATTTAACGCGAATTTTAACAAAATATTAACGTTTACAATTTTATGGTGCACTCTC
AGTACAATCTGCTCTGATGCCGCATAGTTAAGCCAGCCCCGACACCCGCCAACACCCGCTGACGCGCCCTGACGGGCTTGTCTGCTCCC
GGCATCCGCTTACAGACAAGCTGTGACCGTCTCCGGGAGCTGCATGTGTCAGAGGTTTTCACCGTCATCACCGAAACGCGCGAGACGAA
AGGGCCTCGTGATACGCCTATTTTTATAGGTTAATGTCATGATAATAATGGTTTCTTAGACGTCAGGTGGCACTTTTCGGGGAAATGTG
CGCGGAACCCCTATTTGTTTATTTTTCTAAATACATTCAAATATGTATCCGCTCATGAGACAATAACCCTGATAAATGCTTCAATAATA
TTGAAAAAGGAAGAGTATGAGTATTCAACATTTCCGTGTCGCCCTTATTCCCTTTTTTGCGGCATTTTGCCTTCCTGTTTTTGCTCACC
CAGAAACGCTGGTGAAAGTAAAAGATGCTGAAGATCAGTTGGGTGCACGAGTGGGTTACATCGAACTGGATCTCAACAGCGGTAAGATC
CTTGAGAGTTTTCGCCCCGAAGAACGTTTTCCAATGATGAGCACTTTTAAAGTTCTGCTATGTGGCGCGGTATTATCCCGTATTGACGC
CGGGCAAGAGCAACTCGGTCGCCGCATACACTATTCTCAGAATGACTTGGTTGAGTACTCACCAGTCACAGAAAAGCATCTTACGGATG
GCATGACAGTAAGAGAATTATGCAGTGCTGCCATAACCATGAGTGATAACACTGCGGCCAACTTACTTCTGACAACGATCGGAGGACCG
AAGGAGCTAACCGCTTTTTTGCACAACATGGGGGATCATGTAACTCGCCTTGATCGTTGGGAACCGGAGCTGAATGAAGCCATACCAAA
CGACGAGCGTGACACCACGATGCCTGTAGCAATGGCAACAACGTTGCGCAAACTATTAACTGGCGAACTACTTACTCTAGCTTCCCGGC
AACAATTAATAGACTGGATGGAGGCGGATAAAGTTGCAGGACCACTTCTGCGCTCGGCCCTTCCGGCTGGCTGGTTTATTGCTGATAAA
TCTGGAGCCGGTGAGCGTGGGTCTCGCGGTATCATTGCAGCACTGGGGCCAGATGGTAAGCCCTCCCGTATCGTAGTTATCTACACGAC
GGGGAGTCAGGCAACTATGGATGAACGAAATAGACAGATCGCTGAGATAGGTGCCTCACTGATTAAGCATTGGTAACTGTCAGACCAAG
TTTACTCATATATACTTTAGATTGATTTAAAACTTCATTTTTAATTTAAAAGGATCTAGGTGAAGATCCTTTTTGATAATCTCATGACC
AAAATCCCTTAACGTGAGTTTTCGTTCCACTGAGCGTCAGACCCCGTAGAAAAGATCAAAGGATCTTCTTGAGATCCTTTTTTTCTGCG
CGTAATCTGCTGCTTGCAAACAAAAAAACCACCGCTACCAGCGGTGGTTTGTTTGCCGGATCAAGAGCTACCAACTCTTTTTCCGAAGG
TAACTGGCTTCAGCAGAGCGCAGATACCAAATACTGTTCTTCTAGTGTAGCCGTAGTTAGGCCACCACTTCAAGAACTCTGTAGCACCG
CCTACATACCTCGCTCTGCTAATCCTGTTACCAGTGGCTGCTGCCAGTGGCGATAAGTCGTGTCTTACCGGGTTGGACTCAAGACGATA
GTTACCGGATAAGGCGCAGCGGTCGGGCTGAACGGGGGGTTCGTGCACACAGCCCAGCTTGGAGCGAACGACCTACACCGAACTGAGAT
ACCTACAGCGTGAGCTATGAGAAAGCGCCACGCTTCCCGAAGGGAGAAAGGCGGACAGGTATCCGGTAAGCGGCAGGGTCGGAACAGGA
GAGCGCACGAGGGAGCTTCCAGGGGGAAACGCCTGGTATCTTTATAGTCCTGTCGGGTTTCGCCACCTCTGACTTGAGCGTCGATTTTT
GTGATGCTCGTCAGGGGGGCGGAGCCTATGGAAAAACGCCAGCAACGCGGCCTTTTTACGGTTCCTGGCCTTTTGCTGGCCTTTTGCTC
ACATGTTCTTTCCTGCGTTATCCCCTGATTCTGTGGATAACCGTATTACCGCCTTTGAGTGAGCTGATACCGCTCGCCGCAGCCGAACG
ACCGAGCGCAGCGAGTCAGTGAGCGAGGAAGCGGAAGA
